# Joint trajectories of brain atrophy, white matter hyperintensities and cognition quantify brain maintenance

**DOI:** 10.1038/s41467-026-74957-2

**Published:** 2026-07-04

**Authors:** Inga Menze, Jose Bernal, Rogier A. Kievit, Kumar Parijat Tripathi, Timo Kaleck, Renat Yakupov, Slawek Altenstein, Claudia Bartels, Katharina Buerger, Michaela Butryn, Peter Dechent, Michael Ewers, Klaus Fliessbach, Ingo Frommann, Maria Gemenetzi, Wenzel Glanz, Daria Gref, Julian Hellmann-Regen, Stefan Hetzer, Enise I. Incesoy, Daniel Janowitz, Ingo Kiliman, Luca Kleineidam, Marie Theres Kronmüller, Christoph Laske, Debora Melo van Lent, Falk Lüsebrink, Robert Perneczky, Oliver Peters, Lukas Preis, Josef Priller, Boris-Stephan Rauchmann, Ayda Rostamzadeh, Sandra Roeske, Klaus Scheffler, Björn-Hendrik Schott, Anja Schneider, Sebastian Sodenkamp, Annika Spottke, Eike Jakob Spruth, Melina Stark, Stefan Teipel, Michael Wagner, Jens Wiltfang, Frank Jessen, Alfredo Ramirez, Stefanie Schreiber, Emrah Düzel, Gabriel Ziegler

**Affiliations:** 1 German Centre for Neurodegenerative Diseases (DZNE), Magdeburg, Germany; 2 Institute of Cognitive Neurology and Dementia Research, Otto von Guericke University Magdeburg, Magdeburg, Germany; 3 Department Artificial Intelligence in Biomedical Engineering (AIBE), Friedrich-Alexander-Universität Erlangen-Nürnberg (FAU), Erlangen, Germany; 4 Institute for Neuroscience and Cardiovascular Research, Row Fogo Centre for Research into Ageing and The Brain, Department of Neuroimaging Sciences, The University of Edinburgh, Edinburgh, UK; 5 Faculty of Medicine, Friedrich-Alexander-Universität Erlangen-Nürnberg (FAU), Erlangen, Germany; 6 Donders Institute for Brain, Cognition and Behavior, Radboud University Medical Centre, Nijmegen, The Netherlands; 7 Division of Neurogenetics and Molecular Psychiatry, Department of Psychiatry and Psychotherapy, Faculty of Medicine and University Hospital Cologne, University of Cologne, Cologne, Germany; 8 German Centre for Neurodegenerative Diseases (DZNE), Berlin, Germany; 9 Department of Psychiatry and Psychotherapy, Charité, Berlin, Germany; 10 Department of Psychiatry and Psychotherapy, University Medical Centre Goettingen, Georg August University of Goettingen, Goettingen, Germany; 11 German Centre for Neurodegenerative Diseases (DZNE), Munich, Germany; 12 Institute for Stroke and Dementia Research (ISD), University Hospital, LMU Munich, Munich, Germany; 13 MR-Research in Neurosciences, Department of Cognitive Neurology, University Medical Centre Goettingen, Goettingen, Germany; 14 German Centre for Neurodegenerative Diseases (DZNE), Bonn, Germany; 15 Department of Old Age Psychiatry and Cognitive Disorders, University of Bonn, University Hospital Bonn, Bonn, Germany; 16 Department of Psychiatry and Neurosciences, Charité Universitätsmedizin Berlin, Berlin, Germany; 17 Charité Universitätsmedizin Berlin, ECRC Experimental and Clinical Research Center, Berlin, Germany; 18 Berlin Centre for Advanced Neuroimaging, Charité – Universitätsmedizin Berlin, Berlin, Germany; 19 Vivantes Klinikum Am Urban, Department of Psychiatry, Psychotherapy, and Psychosomatics, Vivantes Urban Hospital, Berlin, Germany; 20 German Centre for Neurodegenerative Diseases (DZNE), Rostock, Germany; 21 Department of Psychosomatic Medicine, Rostock University Medical Center, Rostock, Germany; 22 German Centre for Neurodegenerative Diseases (DZNE), Tübingen, Germany; 23 Section for Dementia Research, Hertie Institute for Clinical Brain Research and Department of Psychiatry and Psychotherapy, University of Tübingen, Tübingen, Germany; 24 Glenn Biggs Institute for Alzheimer’s and Neurodegenerative Diseases, UT Health San Antonio, San Antonio, TX USA; 25 Department of Psychiatry and Psychotherapy, University Hospital, LMU Munich, Munich, Germany; 26 Munich Cluster for Systems Neurology (SyNergy), Munich, Germany; 27 Ageing Epidemiology Research Unit (AGE), School of Public Health, Imperial College London, Charing Cross Hospital, London, UK; 28 Department of Psychiatry and Psychotherapy, School of Medicine and Health, Technical University of Munich, and German Centre for Mental Health (DZPG), Munich, Germany; 29 Sheffield Institute for Translational Neuroscience (SITraN), University of Sheffield, Sheffield, UK; 30 Department of Neuroradiology, University Hospital LMU, Munich, Germany; 31 Department of Psychiatry, University of Cologne, Medical Faculty, Cologne, Germany; 32 Department for Biomedical Magnetic Resonance, University of Tübingen, Tübingen, Germany; 33 German Centre for Neurodegenerative Diseases (DZNE), Goettingen, Germany; 34 Leibniz Institute for Neurobiology, Magdeburg, Germany; 35 Department of Psychiatry and Psychotherapy, University of Tübingen, Tübingen, Germany; 36 Department of Neurology, University of Bonn, Bonn, Germany; 37 Neurosciences and Signaling Group, Institute of Biomedicine (iBiMED), Department of Medical Sciences, University of Aveiro, Campus Universitario de Santiago, Aveiro, Portugal; 38 Excellence Cluster on Cellular Stress Responses in Aging-Associated Diseases (CECAD), University of Cologne, Cologne, Germany; 39 Department of Neurology, Otto von Guericke University Magdeburg, Magdeburg, Germany; 40 Institute of Cognitive Neuroscience, University College London, London, UK

**Keywords:** Neurology, Cognitive ageing, Neural ageing, Risk factors

## Abstract

Brain maintenance – the preservation of brain structure or function relevant to cognitive performance – remains challenging to quantify. Here, we propose a domain-general brain maintenance index derived by jointly modelling the longitudinal co-evolution of ageing-related atrophy (via medial temporal lobe to ventricle ratio, MTLV-ratio), white matter hyperintensities (WMH), and global cognition assessed by the preclinical Alzheimer’s cognitive composite (PACC5) using latent growth curve modelling. We demonstrate its utility in 543 cognitively unimpaired older adults from the DELCODE cohort, followed annually over four years. We show that changes in MTLV-ratio and WMH additively predict cognitive change. We further show that higher neuroticism, depressive symptoms, lower openness, and faster biological ageing are related to unfavourable domain-specific trajectories and poorer brain maintenance. Our findings highlight the combined relevance of WMH and ageing-related atrophy dynamics for brain maintenance. Maintaining cerebrovascular and mental health alongside cognitive engagement could promote brain maintenance, delay cognitive decline and dementia.

## Abstract (web summary)

Joint longitudinal modelling of brain atrophy, white matter damage, and cognition in 543 older adults yielded a brain maintenance index. Poorer mental health, lower openness, and faster biological ageing predicted reduced maintenance.

## Introduction

Although declines in cognitive abilities and brain integrity are expected with ageing^1,2^, some individuals preserve brain structure relevant to cognition for years, owing to their inherent or acquired capacity to attenuate ageing- and pathology-related changes^3–7^. This phenomenon, known as brain maintenance^8,9^, remains incompletely understood but represents a major research priority in neurocognitive ageing, given its implications for successful ageing and its relevance for preventive and therapeutic strategies.

From a modelling perspective, characterising brain maintenance requires capturing coupled changes between brain integrity and cognitive function over time, i.e. the extent to which cognition is preserved because the brain itself remains structurally and functionally intact. Meeting this theoretical requirement entails^9^: (i) identifying neural alterations that accompany ageing and contribute to cognitive decline; (ii) adopting modelling frameworks capable of capturing system-level dynamics, in which longitudinal interrelationships between cognitive and brain processes are jointly modelled rather than treated as independent trajectories; (iii) isolating the specific component of cognitive ageing attributable to preserved brain structure; (iv) and identifying factors that contribute to brain changes in order to maintain brain health and consequently cognitive outcomes. These may include modifiable lifestyle factors, including cognitive, social, and physical activity, cardiovascular risk factors, dietary patterns, as well as psychological risk factors like depression, which all were linked to cognitive and brain health outcomes^10–12^.

The first requirement—identifying cognitively relevant brain changes—has been extensively studied. Established changes include ageing-related atrophy in frontal regions and the medial temporal lobe (MTL)^13–15^, as well as cerebrovascular alterations linked to cerebral small vessel disease (CSVD)^7,15,16^, most notably white matter hyperintensities (WMH)^17,18^. In contrast, the second requirement—modelling system-level dynamics—remains actively debated^19,20^. Reported findings range from no significant associations between WMH and brain atrophy^21^ to their co-occurrence being attributed to shared risk factors such as Alzheimer’s disease (AD)^22^, to evidence that WMH directly contribute to brain atrophy^23,24^, particularly in MTL structures^20,23^, and to bidirectional models in which they exacerbate one another over time^4,25,26^.

Likewise, evidence regarding their unique versus synergistic contributions to cognitive decline remains inconclusive, especially in longitudinal studies. Some prospective studies in older adults have demonstrated independent^21^ or interactive effects^27,28^ of baseline markers of ageing-related atrophy and cerebrovascular abnormalities on cognitive trajectories, with outcomes depending on age group and cognitive domain. Studies examining simultaneous changes in these neurocognitive domains yielded ambiguous results. Some suggest interactive effects of hippocampal atrophy and WMH progression on episodic and working memory changes over nine years^29^, while others associate episodic memory changes over 15 years primarily with four-year hippocampal atrophy^3^. In a study examining simultaneous change over three years, changes in WMH, total brain-, grey- and white matter volume, and cognition were significantly interrelated^24^.

To our knowledge, the third requirement of the brain maintenance framework—isolating the component of cognitive ageing attributable specifically to structural preservation^9^—has not yet been explicitly addressed. We therefore set out to achieve three complementary objectives. First, we modelled coupled longitudinal changes across cerebrovascular pathology, brain atrophy, and cognitive performance within a unified multivariate latent growth curve modelling (LGCM) framework. We thus extend prior work on pairwise associations by a parsimonious model able to validate global fit of the coupled system. Second, we derived individual cognitive trajectories as a function of joint structural change, enabling explicit quantification of the proportion of cognitive ageing explained by preserved brain integrity. This provides a principled operationalisation of a brain maintenance index as a longitudinal, multi-domain, system-level construct. Third, we identified modifiable lifestyle factors and personality traits that contribute to inter-individual differences in baseline levels and longitudinal change across neurocognitive domains and our proposed domain-general brain maintenance index.

We demonstrate the utility of this framework in a large cohort of cognitively unimpaired older adults followed annually over four years, yielding mechanistic insight into the neurobiological basis of brain maintenance and identifying potential targets for preventive strategies against cognitive decline.

## Results

### Descriptive statistics and sample characteristics

Among the 722 cognitively unimpaired DELCODE participants, 543 attended a minimum of two annual visits (52.85% female; mean age 69.99 ± 5.87 years; mean years of education: 14.82 ± 2.92 years; 27.88 % APOE4 carriers; Supplementary Fig. 1A). The average number of visits attended per participant was approximately four (3.79, 95%-CI [3.70, 3.88]; Supplementary Fig. 1B). Detailed descriptions of the self-report questionnaires used to assess multiple potentially modifiable lifestyle factors and personality traits and their respective descriptive statistics are provided in Supplementary Tables 1 and 2.

### Longitudinal interrelations across neurocognitive domains of ageing-related atrophy, WMH, and cognition

In order to characterize latent-level interrelated changes across the three neurocognitive domains on a global level, we specified a global trivariate LGCM model including total WMH volumes, ageing-related atrophy, and cognition, accounting for retest-related practice effects (see methods Latent Growth Curve Modelling). Ageing-related atrophy was operationalised as the MTL-to-ventricle ratio (MTLV-ratio), a measure previously shown to be sensitive to ageing and cognitive decline^30,31^. Cognitive performance was assessed using the preclinical Alzheimer’s cognitive composite score (PACC5)^32^. We additionally specified two supplemental regional models including frontal WMH and cortical volumes, and posterior WMH and parietal cortical volumes, respectively (see methods Latent Growth Curve Modelling). Before specifying each trivariate model, we characterized mean change processes within each domain (see methods Latent Growth Curve Modelling and Supplementary Table 3). While linear latent change adequately captured trajectories of total and regional WMH, frontal and parietal cortical volumes, as well as cognition, decline in MTLV-ratio required both linear and nonlinear latent change components.

**Fig. 1:**
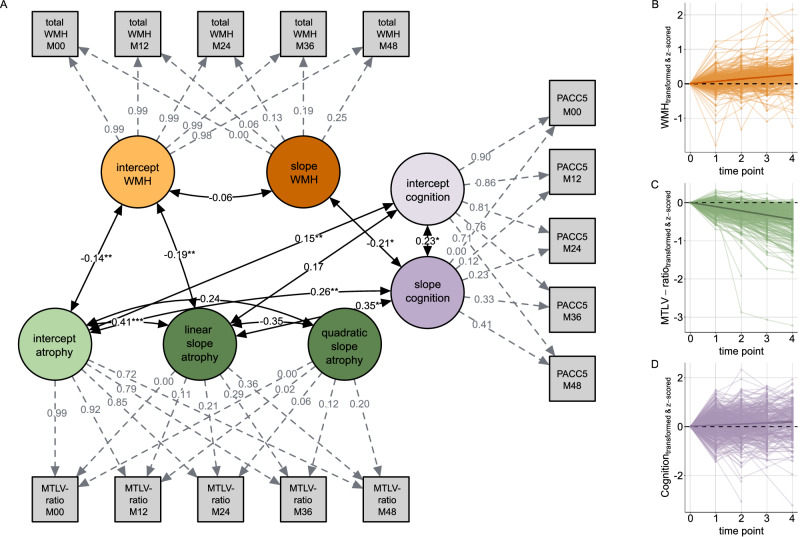
Illustration of the trivariate latent growth curve model linking changes in three neurocognitive domains. **A** We examined the interrelationship between three processes: cerebrovascular abnormalities, ageing-related atrophy, and cognition, operationalised here as white matter hyperintensities (WMH), medial temporal lobe to ventricle ratio (MTLV-ratio), and PACC5 performance, respectively, using latent growth curve modelling over five annual assessment time points (M00 through M48). We adjusted the model for age, sex, years of education, and in the case of WMH and MTLV-ratio for total intracranial volume (TICV). For readability, regressions of the covariates on latent intercepts and latent slopes (for information see Supplementary Table 4), intercepts and error variances, cross-domain residual coupling, and the retest-related manifest offset in PACC5 are omitted. Standardized path coefficients are reported with two-sided Z (Wald) tests from the lavaan-based trivariate LGCM with robust maximum likelihood estimation (MLR); cross-domain *p* values are not corrected for multiple comparisons. Fixed paths are depicted via dotted lines. For readability, only significant or trend-wise associations in cross-domain relations are depicted. * *p* < 0.05, ** *p* < 0.01, *** *p* < 0.001. **B**–**D** Change from baseline in the three neurocognitive domains of interest, operationalised by subtracting baseline estimates from all follow-up measurements defining baseline estimates to be zero. **B** WMH volumes increase over follow-ups. Total WMH volumes were log10-transformed and *z*-scored. **C** MTLV-ratio decreases over follow-ups. MTLV-ratio was Box-Cox-transformed and *z*-scored. **D** PACC5 performance weakly but nonsignificantly increases across all participants and follow-ups. PACC5 performance was Yeo-Johnson transformed and z-scored. Source data are provided as a Source Data file.

The global trivariate LGCM model showed good model fit (*χ²*(135) = 155.06, *p* = 0.114; *CFI* = 0.998; *RMSEA* = 0.022 [0.000, 0.038]; *SRMR* = 0.018; *Yuan-Bentler scaling factor* = 1.081; Fig. 1A; see Supplementary Table 4 for detailed model information). Results of regional-level analyses are reported in Supplementary Tables 5, 6 and Supplementary Fig. 2.

We tested for effects of assumed shared risk factors by including cardiovascular risk, APOE-ε4 and plasma Aβ42/40, plasma pTau181, and plasma NfL into the global model separately (Supplementary Fig. 3; Supplementary Table 7-10). Cardiovascular risk was weakly related to higher baseline WMH volumes (*β* = 0.078, *Z* = 1.968, *p* = 0.049, 95%-CI [0.00, 0.15]). While plasma Aβ42/40 did not relate to any of the three neurocognitive domains, APOE-ε4 carriership was related to stronger linear MTLV-ratio decline (*β* = −0.208, *Z* = −2.996, *p* = 0.003, 95%-CI [−0.03, −0.01]). Higher levels of plasma pTau181 and plasma NfL were related to smaller baseline MTLV-ratio (plasma pTau181: *β* = −0.073, *Z* = −2.228, *p* = 0.026, 95%-CI [−0.10, −0.01]; NfL: *β* = −0.123, *Z* = −3.00, *p* = 0.003, 95%-CI [−0.15, −0.03]). Higher plasma pTau181 was also linked to stronger linear MTLV-ratio decline (*β* = −0.173, *Z* = −2.674, *p* = 0.007, 95%-CI [−0.03, −0.00]). Worse baseline PACC5 performance was related to higher plasma pTau181 levels (*β* = −0.092, *Z* = −2.603, *p* = 0.09, 95%-CI [−0.13, −0.02]). Moreover, both plasma pTau181 and plasma NfL related to stronger cognitive decline as assessed via the PACC5 (plasma pTau181: *β* = −0.249, *Z* = −3.239, *p* = 0.001, 95%-CI [−0.05, −0.01]; NfL: *β* = −0.269, *Z* = −3.055, *p* = 0.002, 95%-CI [−0.05, −0.01]). NfL was additionally associated with baseline WMH volumes (*β* = 0.080, *Z* = 2.266, *p* = 0.023, 95%-CI [0.01, 0.14]). Yet, accounting for these additional covariates neither significantly improved model fit nor altered the interpretation of the associations of interest regarding relations across latent variables (Supplementary Table 7-10). Therefore, we present and interpret the results of the parsimonious model, including only the covariates age, sex, years of education, and total intracranial volume (TICV).

#### Neurocognitive changes over time

On average, WMH volumes increased, MTLV-ratios decreased, and PACC5 performance remained stable (intercept of WMH slope: *B* = 1.087, *Z* = 16.85, *p* < 2.2 × 10^−16^, 95%-CI [0.06, 0.08], Fig. 1B; intercept of MTVL-ratio linear slope*: B* = −0.990, *Z* = −14.54*, p* < 2.2 × 10^−16^, 95%-CI [−0.10, −0.08], intercept of MTLV-ratio quadratic slope: *B* = −0.460, *Z* = −3.97*, p* = 7.35 × 10^−5^, 95%-CI [−0.01, −0.00], Fig. 1C; intercept of cognition slope*: B* = 0.124, *Z* = 1.217, *p* = 0.224, 95%-CI [−0.01, 0.04], Fig. 1D). However, both baseline levels and rates of change across domains showed substantial inter-individual variability (Table 1). At the individual level, 89.87% of participants had WMH progression, 88.21% a linear MTLV-ratio decline, and 59.12% positive changes in PACC5. Differences in baseline levels and longitudinal trajectories between cognitively stable and converting individuals are presented in Supplementary Fig. 4A-G.

**Table 1:** Variance of latent intercepts (baseline levels) and slopes (rates of change) of WMH, MTLV-ratio, and cognition as assessed by PACC5

		Est	SE	95% CI	*B*	*Z*	*p*	*R*²
Baseline levels	WMH	0.753	0.044	0.667, 0.838	0.822	17.240	<2.2 × 10^−16^	0.178
	MTLV-ratio	0.308	0.024	0.262, 0.354	0.606	13.069	<2.2 × 10^−16^	0.394
	Cognition	0.383	0.029	0.327, 0.440	0.612	13.285	<2.2 × 10^−16^	0.388
Rates of change	WMH	0.004	0.001	0.002, 0.005	0.980	4.252	2.12 × 10^−5^	0.020
	linear MTLV-ratio	0.006	0.001	0.003, 0.009	0.790	4.138	3.50 × 10^−5^	0.210
	Quadratic MTLV-ratio	0.000	0.000	−0.000, 0.000	0.909	1.757	0.079	0.091
	Cognition	0.011	0.003	0.005, 0.017	0.842	3.640	2.73 × 10^−4^	0.158

*P* values below machine precision are reported as <2.2 × 10^−16^.

Annotations. *WMH* white matter hyperintensities, *MTLV-ratio* medial temporal lobe to ventricle ratio, *PACC5* preclinical Alzheimer’s cognitive composite score, *Est* estimate, *SE* standard error, 95% CI 95% confidence interval, *B* standardized estimate, *Z* *z*-value, *R*² explained variance in latent variables by covariates included in the model, i.e. age, sex, years of education, total intracranial volume.

#### Trajectories of neurocognitive domains are associated to age, sex, and years of education

Supplementary Table 4 provides the full information on all associations regarding age, sex, and years of education and latent intercepts and slopes of the three neurocognitive domains.

##### Age

At baseline, older individuals had lower MTLV-ratios (age → intercept MTLV-ratio: *β* = −0.495, *Z* = −12.609, *p* < 2.2 × 10^−16^, 95%-CI [−0.41, −0.30]), higher WMH volumes (age → intercept WMH: *β* = 0.371, *Z* = 9.250, *p* < 2.2 × 10^−16^, 95%-CI [0.28, 0.43]), and performed worse in PACC5 (age → intercept cognition: *β* = −0.398, *Z* = −9.646, *p* < 2.2 × 10^−16^, 95%-CI [−0.38, −0.25]). Over time, they tended to undergo stronger declines in MTLV-ratios (age → linear slope MTLV-ratio: *β* = −0.434, *Z* = −6.241, *p* = 4.35 × 10^−10^, 95%-CI [−0.05, −0.03]), and showed lower PACC5 performance changes (age → slope cognition: *β* = −0.367, *Z* = −4.783, *p* = 1.73 × 10^−6^, 95%-CI [−0.06, −0.02]).

##### Female sex

Females yielded better baseline PACC5 performance (sex → intercept cognition: *β* = 0.356, *Z* = 9.013, *p* < 2.2 × 10^−16^, 95%-CI [0.22, 0.34]), higher initial MTLV-ratio (sex → intercept MTLV-ratio: *β* = 0.146, *Z* = 3.076, *p* = 0.002, 95%-CI [0.04, 0.17]), but also higher initial WMH volumes (sex → intercept WMH: *β* = 0.166, *Z* = 2.973, *p* = 0.003, 95%-CI [0.05, 0.26]).

##### Education

Individuals with more years of education had better initial PACC5 performance (years of education → intercept cognition: *β* = 0.257, *Z* = 6.337, *p* = 2.34×10^−10^, 95%-CI [0.14, 0.27]), and higher initial MTLV-ratios (years of education → intercept MTLV-ratio: *β* = 0.103, *Z* = 2.720, *p* = 0.007, 95%-CI [0.02, 0.13]). However, years of education did not relate to differential PACC5 performance changes. We observed an education effect on quadratic but not linear MTLV-ratio change, indicating decelerated MTLV-ratio decline with more years of education (years of education → quadratic MTLV-ratio slope: *β* = 0.272, *Z* = 2.291, *p* = 0.022, 95%-CI [0.00, 0.01]). As quadratic MTLV-ratio slope variance was not significant, we advise careful interpretation of this relation. Education did not relate to baseline levels or changes of WMH.

#### Relationships among growth factors of WMH, ageing-related atrophy, and cognition

Individuals with higher baseline WMH volumes had lower baseline MTLV-ratios (intercept WMH ~ intercept MTLV-ratio: cov_Standardized_ = −0.136, *Z* = −3.195, *p* = 0.001, 95%-CI [−0.11, −0.03]) and experienced steeper declines in MTLV-ratios over time (intercept WMH ~ linear slope MTLV-ratio: cov_Standardized_ = −0.186, *Z* = −2.734, *p* = 0.006, 95%-CI [−0.02, −0.00]; Fig. 1A). We did not observe significant associations between faster WMH progression and linear or quadratic MTLV-ratio change.

Individuals with better initial PACC5 performance had higher baseline MTLV-ratios (intercept cognition ~ intercept MTLV-ratio: cov_Standardized_ = 0.151, *Z* = 2.877, *p* = 0.004, 95%-CI [0.02, 0.09]).

**Fig. 2:**
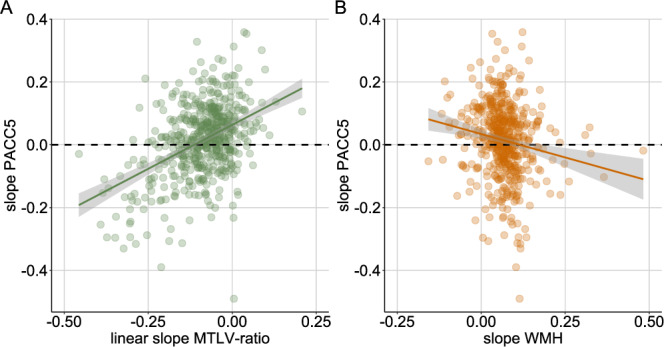
Independent effects of MTLV-ratio and WMH on cognitive changes in ageing. Scatterplot of factor scores of linear slopes of **A** medial temporal lobe to ventricle ratio (MTLV-ratio) and **B** white matter hyperintensities (WMH) on cognition (preclinical Alzheimer’s cognitive composite score; PACC5). Factor scores were extracted from the trivariate latent growth curve model (LGCM) adjusted for effects of age, sex, years of education, and, in the case of WMH and MTLV-ratio, for total intracranial volume (TICV) via regression-based method. Solid lines indicate fitted values. Shaded bands represent 95% confidence intervals for the estimated mean function. We additionally included an interactive HTML-based 3D graphic that is accessible via the supplements (Supplementary Fig. 5) to retain the common representation between the three neurocognitive domains of interest in one space. Source data are provided as a Source Data file.

PACC5 performance changes were more pronounced in individuals with higher MTLV-ratios at baseline, and with slower declines in MTLV-ratios (slope cognition ~ intercept MTLV-ratio: cov_Standardized_ = 0.259, *Z* = 2.838, *p* = 0.005, 95%-CI [0.00, 0.03]; slope cognition ~ linear slope MTLV-ratio: cov_Standardized_ = 0.354, *Z* = 2.439, *p* = 0.015, 95%-CI [0.00, 0.01]; Fig. 1A, Table 2 and Fig. 2A). Additionally, higher progression of WMH was linked to lower PACC5 performance changes (slope cognition ~ slope WMH: cov_Standardized_ = −0.208, *Z* = −2.010, *p* = 0.044, 95%-CI [−0.00, −0.00]; Fig. 1A, Table 2 and Fig. 2B). Collectively, these associations indicate that stronger increase of WMH and MTLV-ratio decline could both impede positive PACC5 performance changes.

**Table 2:** Robust multiple linear regression of independent effects of WMH and MTLV-ratio on cognitive changes in ageing

	*b*	SE	95% CI	*B*	*p*
Intercept	0.12	0.01	0.098, 0.134	0.02	<2.2 × 10^−16^
Slope WMH	−0.23	0.06	−0.343, −0.107	−0.12	1.99 × 10^−4^
Linear Slope MTLV-ratio	0.67	0.07	0.539, 0.804	0.52	<2.2 × 10^−16^
Quadratic Slope MTLV-ratio	3.38	0.36	2.662, 4.092	0.36	<2.2 × 10^−16^
Intercept PACC5	0.03	0.01	0.019, 0.042	0.21	3.42 × 10^−7^
Intercept WMH	−0.00	0.00	−0.012, 0.006	−0.03	0.480
Intercept MTLV-ratio	−0.02	0.01	−0.030, −0.002	−0.10	0.028

Additive robust multiple linear regression of latent PACC5 slopes regressing on latent WMH and MTLV-ratio slopes, while controlling for latent intercepts of all neurocognitive domains. Factor scores were extracted from the trivariate LGCM adjusted for effects of age, sex, years of education, and, in the case of WMH and MTLV-ratio for total intracranial volume (TICV) via regression-based method. *P* values below machine precision are reported as <2.2 × 10^−16^.

Annotations. *WMH* white matter hyperintensities, *MTLV-ratio* medial temporal lobe to ventricle ratio, *PACC5* preclinical Alzheimer’s cognitive composite score, *b* regression coefficient, *SE* standard error, 95% CI 95% confidence interval, *B* standardized regression coefficient.

#### Unique contributions of ageing-related atrophy and WMH to cognitive ageing

To assess the specific contributions of changes in WMH and MTLV-ratio to cognitive change, we conducted a robust multiple linear regression using the extracted regression-based factor score estimates for each individual, while controlling for effects of latent intercepts (*R²*_*adjusted*_ = 0.392; Table 2). Latent rates of changes of PACC5 were uniquely associated with each brain-level domain slope, i.e. linear (*B* = 0.52, *p* < 2.2 × 10^−16^, 95%-CI [0.54, 0.80]) and quadratic MTLV-ratio decline (*B* = 0.36, *p* < 2.2 × 10^−16^, 95%-CI [2.66, 4.09]), and WMH (*B* = −0.12, *p* = 1.99 × 10^−4^, 95%-CI [−0.34, −0.11]) (Table 2 and Fig. 2). We did not observe indications for a detrimental interaction of these processes (*F*_1, 536_ = 0.622, *p* = 0.430).

### Modifiable lifestyle factors and personality traits associate to changes in neurocognitive domains and brain maintenance

After characterising individuals’ neurocognitive ageing trajectories in the three domains and their interrelations, we studied which modifiable lifestyle factors and personality traits related to the progression of WMH, MTLV-ratio decline, or PACC5 changes specifically and generally.

**Fig. 3:**
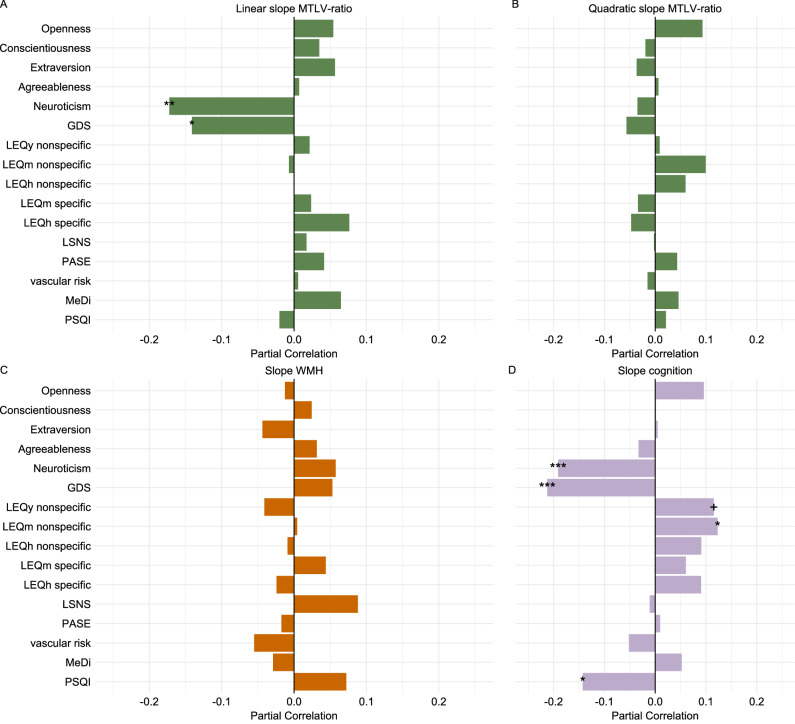
Associations of latent changes in neurocognitive domains with personality and modifiable lifestyle factors. Factor scores for latent slopes were derived from the trivariate latent growth curve model (LGCM) via regression-based method. We used two-sided partial Spearman’s correlations to account for the effects of age, sex, years of education, and total intracranial volume (TICV). All correlations were FDR-corrected. Panels show relations between personality and lifestyle factors and **A** linear and **B** quadratic slopes of medial temporal lobe to ventricle ratio (MTLV-ratio), linear slopes of **C** total white matter hyperintensities (WMH), and **D** cognition as assessed with the preclinical Alzheimer’s cognitive composite score (PACC5). *** *p* < 0.001, ** *p* < 0.01, * *p* < 0.05, +*p* < 0.1. Personality traits were acquired via the Big Five Inventory BFI-10. GDS = geriatric depression scale. LEQ = lifetime experiences questionnaire assessed for three life periods: y = young adulthood (13–30 years), m = midlife (30–65 years), h = late life (≥65 years or from retirement onward). LSNS = Lubben social network scale. PASE = Physical Activity Scale for the elderly. MeDi = Mediterranean diet. PSQI = Pittsburgh Sleep Quality Index (CAVE: by convention, higher values denote lower sleep quality). Source data are provided as a Source Data file.

First, we used the domain-specific extracted factor scores of latent slopes and correlated them against the lifestyle and personality factors (Fig. 3; correlation with latent intercepts and lifestyle factors in Supplementary Fig. 6). We used partial Spearman’s correlations to adjust for the effects of age, sex, years of education, and TICV (Supplementary Fig. 7 shows the full correlation matrix). We show unadjusted correlations in Supplementary Figs.  8 and 9.

We did not observe any lifestyle factors to be associated with WMH progression after correction for multiple comparisons. Yet, we found personality and multiple modifiable lifestyle factors to be associated with changes in MTLV-ratio and PACC5. First, higher late-life depressive symptoms, assessed via the geriatric depression scale (GDS), and higher levels of neuroticism were linked to steeper MTLV-ratio decline (GDS: *ρ* = −0.141, *p*_*FDR*_ = 0.018, 95%-CI [−0.22, −0.06]; neuroticism: *ρ* = −0.172, *p*_*FDR*_ = 0.002, 95%-CI [−0.25, −0.09]) and lower PACC5 performance changes (GDS: *ρ* = −0.213, *p*_*FDR*_ = 7.91×10^−5^, 95%-CI [−0.29, −0.13]; neuroticism: *ρ* = −0.191, *p*_*FDR*_ = 4.37×10^−4^, 95%-CI [−0.27, −0.11]). Additionally, PACC5 performance changes were associated with poorer sleep quality, assessed via the Pittsburgh sleep quality index (PSQI: *ρ* = −0.143, *p*_*FDR*_ = 0.023, 95%-CI [−0.23, −0.05]), and weakly related to leisure time activities in midlife (LEQm nonspecific: *ρ* = 0.123, *p*_*FDR*_ = 0.043, 95%-CI [0.04, 0.21]) and by trend in young adulthood (LEQy nonspecific: *ρ* = 0.115, *p*_*FDR*_ = 0.063, 95%-CI [0.04, 0.20]). Leveraging robust multiple linear regression, we found that beyond age, sex, and years of education, the personality and modifiable lifestyle factors of interest contributed low to the variance in WMH changes (*R²* = 0.109, *R²*_*adjusted*_ = 0.047), and MTLV-ratio decline (linear: *R²* = 0.071, *R²*_*adjusted*_ = 0.005; quadratic: *R²* = 0.098, *R²*_*adjusted*_ = 0.034). Personality and  modifiable lifestyle factors explained approximately 9.17% of variance in PACC5 changes (*R²* = 0.152, *R²*_*adjusted*_ = 0.092). Supplementary Fig. 10 shows education-related differences in associations between lifestyle factors and personality traits and neurocognitive domains. We show associations between personality and lifestyle factors and regional WMH and cortical volumes in Supplementary Figs. 11 and 12.

**Fig. 4:**
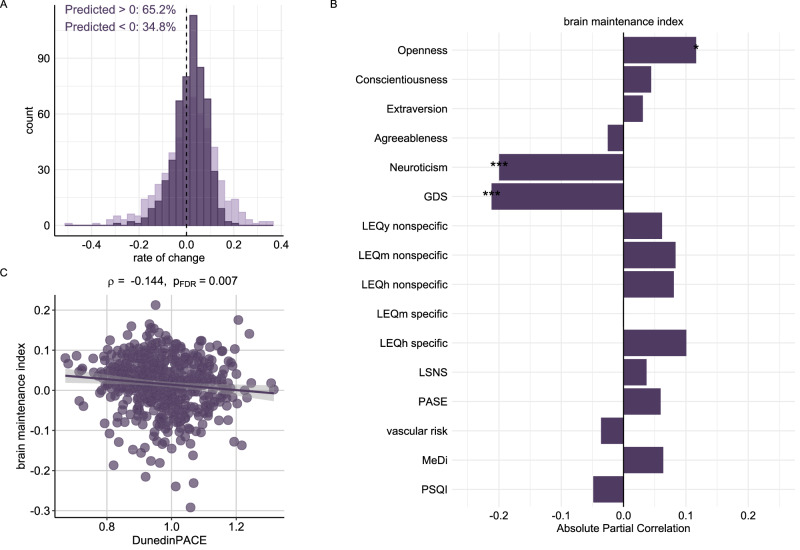
Individual maintenance as brain-structure–related cognitive ageing. Brain maintenance index was derived as predicted cognitive slope from a robust multiple linear regression including brain-domain changes and baseline levels of all neurocognitive domains (see Unique contributions of ageing-related atrophy and WMH to cognitive ageing), reflecting the brain-structure–related component of cognitive ageing. Higher individual predicted values hence indicate preserved cognitive functioning in proportion to preserved brain integrity, consistent with successful brain maintenance. **A** Distribution of the brain-structure–related component of cognitive ageing (darker purple) against factor scores of cognitive rates of change derived from the trivariate latent growth curve model (LGCM) via regression-based method (lighter purple). **B** FDR-corrected two-sided partial Spearman’s correlations (accounting for the effects of age, sex, years of education, and total intracranial volume) show relations between lifestyle factors and personality traits and the brain-structure–related component of cognitive ageing. *** *p* < 0.001, ** *p* < 0.01, * *p* < 0.05, +*p* < 0.1. Personality traits were acquired via the Big Five Inventory BFI-10. GDS = geriatric depression scale. LEQ = lifetime experiences questionnaire assessed for three life periods: y = young adulthood (13–30 years), m = midlife (30–65 years), *h* = late life (≥65 years or from retirement onward). LSNS = Lubben social network scale. PASE = Physical Activity Scale for the elderly. MeDi = Mediterranean diet. PSQI = Pittsburgh Sleep Quality Index (CAVE: by convention, higher values denote lower sleep quality). **C** FDR-corrected two-sided partial Spearman’s correlations between brain maintenance index and DunedinPACE (molecular marker of biological ageing pace; *n* = 502) as biological validation of the brain maintenance index. Solid lines indicate fitted values. Shaded bands represent 95% confidence intervals for the estimated mean function. Source data are provided as a Source Data file.

Next, we assessed the contributions of modifiable lifestyle factors and personality traits to individual differences in brain maintenance, defined as the brain-structure–related component of cognitive ageing (Fig. 4). This domain-general brain maintenance index was derived as the predicted cognitive slope from the robust multiple regression model including longitudinal brain-domain changes and baseline levels of all neurocognitive domains (see Unique contributions of ageing-related atrophy and WMH to cognitive ageing). Significant associations emerged for late-life depressive symptoms (*ρ* = −0.212, *p*_*FDR*_ = 1.46 × 10^−5^, 95%-CI [−0.29, −0.13]), neuroticism (*ρ* = −0.200, *p*_*FDR*_ = 2.80 × 10^−5^, 95%-CI [−0.28, −0.12]), and openness (*ρ* = 0.116, *p*_*FDR*_ = 0.036, 95%-CI [0.03, 0.20]; Fig. 4B). Importantly, lower DunedinPACE, indicating slower biological ageing, was significantly associated with higher brain maintenance index (*ρ* = −0.144, *p*_*FDR*_ = 0.007, 95%-CI [−0.23, −0.06]; Fig. 4C). Individuals who remained cognitively stable over time exhibited higher brain maintenance than those who subsequently converted to mild cognitive impairment or dementia (Supplementary Fig. 4H).

## Discussion

In this longitudinal study, we investigated brain maintenance at the system-level. Using LGCM, we assessed coupled changes in WMH, ageing-related brain atrophy, and cognitive performance over four years in a relatively large cognitively unimpaired cohort. Building on this framework, we quantified the proportion of cognitive ageing explained by preserved brain integrity, providing an empirical, longitudinal brain maintenance index operationalised as brain structure–related cognitive ageing. Finally, we demonstrate that modifiable lifestyle factors and personality traits are associated with both domain-specific trajectories and our domain-general brain maintenance index.

We observed significant global and regional WMH progression, and declining regional cortical volumes and MTLV-ratio, with the latter accelerating non-linearly over time^33,34^. Global WMH baseline levels notably contributed to ageing-related atrophy, reflected in MTLV-ratio decline. After accounting for population-level retest-related practice effects, mean cognitive performance (PACC5) did not significantly change over time, while interindividual heterogeneity in cognitive changes was substantial. Cognitive decline was faster with steeper MTLV-ratio decline and total WMH progression, highlighting their unique and joint impact on cognitive ageing and relevance for brain maintenance^9^. Openness, neuroticism, and depressive symptoms were particularly key contributors to domain-specific trajectories and domain-general interindividual differences in brain maintenance. Our findings underscore the interdependence of cerebrovascular and ageing-related atrophic changes in shaping cognitive trajectories and highlight promising intervention targets to preserve brain health and cognitive function.

Although WMH generally progressed in our cognitively unimpaired sample, interindividual variability in trajectories implied some individuals experienced greater progression^4,35^, while others may show stable or regressing WMH^36^. Female sex and higher age were related to increased total and frontal WMH baseline burden, but not WMH progression^37^. Regional cortical volume loss and MTLV-ratio decline were faster with increasing age, consistent with lifespan studies showing accelerated volume loss in these structures alongside ventricular enlargement in older age^1,14^. Cognitive performance changes over time were expectedly smaller in older individual^5,6^. We note that the extent of cognitive change, particularly positive change, likely reflected intraindividual practice effects beyond the population-level practice effects controlled for here. Importantly, the extent of such practice effects has been linked to cognitive decline and risk of dementia^38^. We note that while we observed that blood-based biomarkers pTau181 and NfL were related to smaller MTLV-ratios, steeper decline thereof, higher WMH volumes at baseline, and to more pronounced cognitive decline^39,40^, they did not alter the latent associations across neurocognitive domains. Further discussion regarding hidden pathology is provided in the supplements (following Supplementary Tables 7-10)

With regard to the temporal associations of the neurocognitive domains of interest, we note that there were no prominent regional-specific associations. Therefore, we mainly focus on the implications of the trivariate associations identified by our global model.

While we observed significant associations between baseline total WMH and MTLV-ratio, even when controlling for shared risk factors^23,25,41^, their rates of change were not related, in contrast to previous reports^4,25,42–44^. Yet, the absence of such associations has also been observed in relatively healthy ageing cohorts^45^, comparable to the present sample. Nonetheless, higher baseline levels of WMH were associated with steeper MTLV-ratio decline, reinforcing the assumption that this region is vulnerable to cerebrovascular abnormalities^20,23,46^. Our findings partially align with the notion that cerebrovascular changes may play an early and exacerbating role in ageing-related or neuropathological cascades^43,47,48^, and might therefore serve as a proxy for brain maintenance themselves^9,48^. We emphasise, however, that the relationship between WMH and ageing-related atrophy may vary by sample characteristics^18,29,35,42^, and that latent pathology—e.g. in later AD stages—may overshadow the impact of early cerebrovascular abnormalities^23^.

Moreover, we provide evidence for and clarify mechanisms of brain maintenance, demonstrating that changes in MTLV-ratio and total WMH were linked to and exerted additive, independent effects on cognitive performance change^9,24^. While bivariate approaches might obscure this distinction, potentially attributing cognitive decline to a single marker when it is actually driven by both, the present approach highlights the coupled evolution of brain measures and suggests that cerebrovascular pathology compromises cognition not only by accelerating neurodegeneration but also via distinct, direct pathways. WMH progression—rather than baseline WMH^4^—was linked to reduced cognitive performance changes, emphasising the relevance of monitoring and managing WMH progression for mitigating cognitive decline. Conversely, regional levels and progression of WMH did not relate to cognitive changes, suggesting that the total volume of WMH in this sample with relatively low cardiovascular risk might better reflect the cerebrovascular as well as general pathological risk burden. With regard to MTLV-ratio, both baseline levels and decline were associated with cognitive performance changes, underlining the importance of MTL integrity in maintaining cognitive function in ageing^3^. In contrast, regional atrophy did not relate to cognitive changes, as reported elsewhere^24^. Consistent with studies in healthy elderly^35^ and cerebral amyloid angiopathy patients^49^, MTLV-ratio decline explained more variance in cognitive outcomes than WMH. However, the association between baseline total WMH and MTLV-ratio decline over time suggests that cerebrovascular abnormalities may contribute to downstream structural brain changes, reinforcing the idea that early cerebrovascular interventions could promote brain maintenance^43,47,48^. Indeed, longitudinal studies^26,42^ support indirect pathways linking WMH to cognitive decline via structural brain changes. Exploring the consequences and underlying causes of interindividual variability in trajectories of WMH and ageing-related atrophy—and scenarios in which cognitive function remains stable despite structural brain changes—will ultimately inform our understanding of cognitive reserve and brain maintenance^9^. They might require more sophisticated longitudinal analysis approaches in large samples in a lead-lag fashion to explore possible mediation effects.

Notably, several lifestyle factors showed domain-specific and domain-general associations with neurocognitive ageing, offering insight into potential mechanisms of brain maintenance^9^. In line with the concept of differential preservation^6^, the extent to which cognitive functions and structural brain integrity are maintained during ageing may depend on the interindividual expression of specific lifestyle factors. To clarify how different lifestyle characteristics relate to neurocognitive ageing, we examined their associations with domain-specific trajectories and with our proposed domain-general brain maintenance index.

First, we observed selective associations with regard to domain-specific contributions of modifiable lifestyle factors. In this way, cognitively stimulating leisure activity during young adulthood and midlife was related to more favourable cognitive trajectories^11,50^. Education only contributed to baseline levels of cognition and MTLV-ratio^51–53^. Although we found that education was associated with the quadratic MTLV-ratio change, indicating less acceleration of MTLV-ratio decline with more years of education, we advise careful interpretation of this relation since quadratic slope variance was not significant. Consistent with preserved differentiation^6^, education might hence rather contribute to interindividual differences in baseline cognitive functioning or brain reserve, not their rates of change. We note that openness and cognitively stimulating leisure activity during all life periods were related to higher baseline levels of MTLV-ratio and cognitive performance^11^, respectively, highlighting that (seeking for) environmental enrichment may bolster functional integrity of memory networks related to better memory performance in ageing^50^. Additionally, poor sleep quality compromised cognitive performance over time^54^, aligning with evidence on its role as an early marker and potential contributor to neuropathological changes^55^. Compromised sleep quality may also reflect mental health issues, including depression^56^, potentially mediating its impact on cognition^57^.

Indeed, late-life depressive symptoms were prominently and consistently associated with more negative trajectories across the two domain-specific trajectories of cognitive performance^12,58^ and MTLV-ratio decline^1,12,58^, and the domain-general brain maintenance index, even though depressive symptoms were expressed mildly in the present cohort^58^. These findings align with the well-established role of depression as a major dementia risk factor^10,12^. Neuroticism showed a similar pattern of domain-specific and domain-general associations. Neuroticism has been related to depressive symptoms^59,60^—particularly under chronic stress^59^—and appears to contribute to cognitive impairment and dementia conversion risk^60^. Lastly, openness was linked to the brain maintenance index. Openness may promote behaviours supporting cognitive performance and underlying memory networks^61,62^. Importantly, we provide evidence for the validity and relevance of our proposed brain maintenance index by identifying specific predictors that explain interindividual heterogeneity and illustrating its higher expression in cognitively stable compared to converting individuals (Supplementary Fig. 4H). Additionally, we demonstrated its association with DunedinPACE, suggesting that individuals who exhibit slower systemic molecular ageing also show preserved neurocognitive ageing trajectories, providing external validation of the brain maintenance index.

Together, our results hence emphasise the importance of mental health, stress-coping across the lifespan, engaging in a cognitively enriched lifestyle, and promoting personality traits linked to mental health resiliency to promote brain maintenance^9,63–65^. Further research is needed to clarify the underlying mechanisms of these effects.

Several limitations of this study warrant consideration. First, although LGCMs can determine the co-evolution of constructs, they cannot assess the delayed effect of change in one construct on change in another at a later time point. While mediation and moderation hypotheses regarding the temporal sequencing of the three neurocognitive domains are compelling, their evaluation requires longitudinal frameworks that explicitly model lead-lag relationships. Other frameworks, such as latent change score models^66–68^, could enable more nuanced insights into such causal sequences. Second, although we accounted for a population-level practice effect in PACC5 performance, interindividual practice effects could not be reliably estimated, but they may vary in more diverse samples. Future studies are encouraged to explore both ageing and practice. They could inform about meaningful differences in ageing- vs. retest-related effects that could relate to other neurocognitive ageing processes or lifestyle factors^69–71^. Third, given the exclusion criteria, DELCODE participants had low vascular risk, which may have underestimated certain associations, particularly involving WMH. Similar studies in various cohorts are therefore needed to generalize these findings. Fourth, we note that automated segmentation approaches used here may be susceptible to segmentation inaccuracies. However, given the longitudinal multi-centre design of this study, manual correction of all segmentations was not feasible, and a visual quality control procedure was thus applied. Future studies with smaller or targeted samples may adopt alternative quality control strategies. Such approaches must carefully balance potential gains in local anatomical precision against losses in reproducibility, statistical power, and the risk of rater-dependent bias. Fifth, while our analysis was based on a specific operationalisation of the three neurocognitive domains, future research may benefit from further examining lesion load within specific hubs of cognitive networks^46^, clarifying the differential relevance of various CSVD markers^16,17,35,42^, integrating more recently established blood biomarkers alongside biological staging of AD^72^, and expanding the cognitive domains studied. For instance, composite scores targeting executive functions may be additionally informative in the contexts of healthy ageing or vascular cognitive impairment^73,74^. Future research into the combined impact of these factors will further elucidate mechanisms of brain maintenance. Importantly, the proposed operationalisation of brain maintenance requires sufficiently rich multimodal longitudinal data, which may limit its immediate applicability in some research and clinical settings. Large-scale longitudinal studies are needed to operationalise the construct in the first place and to enable its replication and further refinement. While incorporating additional dimensions may allow for more comprehensive modelling approaches, such efforts also increase data demands for achieving robust and generalizable estimates of neurocognitive health outcomes. Sixth, the assessment of modifiable lifestyle factors was non-exhaustive, relying mainly on self-report questionnaires prone to retrospective biases (e.g., LEQ; PASE), and some measures (e.g., cardiovascular risk score) may have lacked precision. Missing data (e.g., MeDi, LEQ late life) may have limited statistical power. These limitations may have reduced our ability to detect stronger effects. Additionally, the relatively short follow-up period may have been insufficient to capture long-term effects, particularly for midlife or earlier lifestyle factors related to brain reserve^64^. Moreover, the role of modifiable lifestyle factors in cognitive reserve in this context remains unaddressed, as does whether their effects are directional or bidirectional^10,11^. Future studies could benefit from more comprehensive and objective lifestyle measures and longitudinal investigations—ideally spanning the full lifespan—to elucidate the mechanisms underlying preserved differentiation and differential preservation^6^, the coupled dynamics among brain structure, cognition, and lifestyle^35^, and their directionality.

In summary, we showed that total WMH may accelerate ageing-related MTL atrophy. Preventing cerebrovascular changes might therefore reduce vulnerability to ageing-related atrophy or pathology-related neurodegeneration—key drivers of cognitive decline and dementia. Together, WMH progression and ageing-related atrophy contribute to cognitive decline, underscoring their relevance for brain maintenance. Importantly, modifiable lifestyle factors influenced ageing-related atrophy and late-life cognition in domain-specific and domain-general ways, offering insight into potential mechanisms of brain maintenance. Our findings highlight the importance of managing cerebrovascular and mental health while fostering cognitive engagement and considering personality traits linked to mental health vulnerability to promote brain maintenance. This approach could not only mitigate the impact of ageing-related processes but also lower the risk of distinct pathological changes and their synergistic interactions during preclinical dementia stages, potentially delaying the onset of overt clinical symptoms and functional impairments.

## Methods

### Study design and participants

This study focussed on baseline and annual follow-up data up to 48 months of 543 cognitively unimpaired individuals from DELCODE (DZNE Longitudinal Cognitive Impairment and Dementia Study^75^)—an observational multi-centre study from the German Centre for Neurodegenerative Diseases (DZNE). We restricted our analysis to individuals who successfully completed at least two visits within the period of 2014 to 2023. Sex was self-reported by participants (287 females, 256 males in the analysis sample). Disaggregated sex and gender information was not collected.

Subjects in DELCODE were ≥60 years old and considered cognitively unimpaired if they performed above −1.5 SD of normal performance on all subtests of the Consortium to Establish a Registry for Alzheimer’s Disease (CERAD) plus test battery adjusting for age, sex, and education. Additional exclusion and inclusion criteria have been previously described in more detail^75^.

All participants gave written informed consent in accordance with the Declaration of Helsinki prior to joining the study. Participants did not receive financial compensation for participation in the DELCODE study. DELCODE is retrospectively registered at the German Clinical Trials Register (DRKS00007966, 04/05/2015). Ethics committees of the medical faculties of all participating sites, Berlin (Charité, University Medicine), Bonn, Cologne, Göttingen, Magdeburg, Munich (Ludwig-Maximilians-University), Rostock, and Tübingen, gave ethical approval for this work. The ethics committee of the medical faculty of the University of Bonn led and coordinated the process.

### Brain imaging data and domains of brain structure

MRI acquisition took place at nine DZNE neuroimaging sites using 3 T Siemens MR scanners. We used structural scans in terms of T1w MPRAGE (full head coverage; 3D acquisition, GRAPPA factor 2, 1 mm^3^ isotropic, 256 × 256 px, 192 sagittal slices, TR/TE/TI 2500/4.33/1100 ms, FA 7°) and T2w FLAIR (full head coverage; 1 mm^3^ isotropic, 256 × 256 px, 192 sagittal slices, TR/TE/TI 5000/394/1800 ms). The DZNE imaging network oversaw operating procedures, quality assurance, and assessment (iNET, Magdeburg)^75^.

#### White matter hyperintensities segmentation

We used WMH of all participants and time points as a proxy for cerebrovascular abnormalities. The WMH quantification procedure was as follows. First, we co-registered T1w and T2w FLAIR images for each individual at each session. Second, we processed the co-registered images with LST-AI^76^. Third, we post-processed the resulting segmentations to reduce false-positive segmentation of WMH. Post-processing also involved excluding lesions that were located outside the supratentorial white matter, specifically those identified in cortical and subcortical grey matter, the brainstem, the choroid plexus, or the septum pellucidum. We derived supratentorial white matter masks based on FreeSurfer’s parcellation as well as on the USCLobes Atlas^77^. We summarised WMH volumes from FreeSurfer’s parcellation masks to retrieve total WMH volume as a global measure for cerebrovascular abnormalities. For regional analyses, we used frontal and parietooccipital WMH from the USCLobes Atlas. We here refer to parietooccipital WMH as posterior WMH. We decided on an anterior-posterior differentiation, in line with previous findings of associating posterior WMH with AD pathology related changes, and frontal WMH with vascular-related conditions^78,79^.

#### Quantification of ageing-related atrophy

We used FreeSurfer’s longitudinal pipeline for segmentation of T1-weighted MPRAGE images. Automatically generated segmentation and parcellation maps were overlaid on the corresponding MPRAGE images and visually inspected by trained raters to identify and discard gross errors or abnormalities (e.g., topological defects, mislabelled cortical regions, or failed skull stripping).

On a global level, we focused on the aggregated volumes of the hippocampus, entorhinal cortex, parahippocampal cortex, and amygdala, which are broadly considered key structures of the MTL, as well as volumes of the inferior lateral ventricles. As a measure of ageing-related brain atrophy and MTL integrity, we computed the MTL-to-ventricle ratio (MTLV-ratio):

$$\left(\frac{{volumes}\,{of}\,{MTL}-{related}\,{regions}}{{volumes}\,{of}\,{MTL}-{related}\,{regions}+{inferior}\,{lateral}\,{ventricle}\,{volume}}\right)\times 100.$$

Similar measures, such as the previously proposed hippocampal-to-ventricle ratio, have been shown to be more sensitive to ageing and cognitive decline than absolute volume measures of brain tissue^30,31^.

Moreover, we estimated frontal and parietal cortical volume, corresponding to frontal and posterior WMH. Frontal and parietal cortical volumes were defined as aggregated volumes across hemispheres based on Freesurfer’s automatic volumetric cortical parcellation, in accordance with the original suggestions by Desikan et al.^80^ (Table 3).

**Table 3:** Definition of frontal and parietal cortical regions with cortical parcellations from Freesurfer in accordance with Desikan et al. (2006)

Frontal cortical volume	Parietal cortical volume
• Superior frontal • Rostral middle frontal • Caudal middle frontal • Pars opercularis • Pars triangularis • Pars orbitalis • Lateral orbitofrontal • Medial orbitofrontal • Precentral • Paracentral • Frontal pole	• Superior parietal • Inferior parietal • Supramarginal • Postcentral • Precuneus

We estimated segmentation-based total intracranial volume (TICV) from baseline, which relies on Freesurfer’s samseg-based structure segmentation^81^, including CSF and other intracranial non-brain structures (https://surfer.nmr.mgh.harvard.edu/fswiki/sbTIV).

### Cognitive domain

We assessed cognitive performance annually using the established preclinical Alzheimer’s cognitive composite score (PACC5)^32^, a measure for early cognitive change specific to AD. PACC5 was chosen to enhance comparability across studies, since composite scores have been shown to be more sensitive to ageing-related cognitive change than individual test measures^82,83^. The PACC5 comprises measures of processing speed, global cognition, and particularly memory—cognitive domains that are particularly vulnerable to AD pathology but also show ageing-related decline in cognitively healthy individuals. The PACC5 is the averaged z-standardised performance on the Mini Mental State Examination (MMSE), Wechsler Memory Scale Revised (WMS-R) logical memory delayed recall, Symbol-Digit-Modalities-Test (SDMT), free and total recall of the Free and Cued Selective Reminding Test (FCSRT), and semantic fluency. Three parallel test versions were available for SDMT and FCSRT. Individual-level z-scores were derived from the baseline mean and SD of the cognitively unimpaired individuals.

### Modifiable lifestyle factors and personality traits

To examine contributors to trajectories of each neurocognitive domain (domain-specific) or across them (domain-general) in the context of brain maintenance, we assessed multiple potentially modifiable lifestyle factors via self-report questionnaires, including cardiovascular risk, late-life depressive symptoms, Mediterranean diet (MeDi), physical activity, sleep quality, social network, and lifetime experiences. Additionally, we assessed personality traits. Some of these factors were collected once during the baseline visit and some on a continuous basis during each annual visit (overview in Supplementary Table 1). We here used personality traits and modifiable lifestyle factors reported at baseline to examine how the manifestation at baseline relates to neurocognitive domains at baseline and their change.

### Statistical analysis

The goals of our model-based analysis of joint neurocognitive changes to study brain maintenance were three-fold: First, we established linked changes across the three neurocognitive domains. Second, we explored unique contributions of changes in brain-structure domains to cognitive ageing in order to operationalise and validate an index for brain maintenance. Third, we sought to identify modifiable lifestyle factors and personality traits that contributed domain-specific or domain-general to the preservation of these linked neurocognitive domains in ageing.

#### Data transformation

To account for undesired effects of potential skewness, we log10-transformed total and regional WMH volumes, Box-Cox transformed MTLV-ratio and regional cortical volumes, and Yeo-Johnson transformed PACC5. All variables were z-scored (pooled across time points) before entering the model. Original distributions of all variables across time are provided in Supplementary Fig. 13.

#### Latent growth curve modelling

For multi-domain trajectory modelling and maintenance analysis we leveraged latent growth curve modelling (LGCM)^84^, a flexible and powerful class of structural equation models (SEM). This approach allows for the estimation of both latent baseline levels (intercepts) and longitudinal change (slopes) in constructs of interest, while accounting for covariate effects. In the context of this study, we use the term domain to refer to the latent constructs representing WMH, brain morphometric measures, and cognitive performance. We employed a trivariate LGCM to obtain a parsimonious, internally coherent estimation of measurement parameters, growth factors, and covariate effects within one joint likelihood. This unified estimation avoids multiple-comparison inflation, prevents redundant parameter estimation, ensures appropriate standard errors and is essential for identifying a latent brain maintenance index based on simultaneously estimated brain-related slopes.

Specifically, the LGCM allowed us to examine the following:

1. How are the baseline levels of WMH, brain morphometric measures, and cognitive performance associated with one another (covariance between latent intercepts)?
2. How do WMH, brain morphometric measures, and cognitive performance change over the course of four years, and is there interindividual variability in latent change (slopes)?
3. Are the latent intercepts and latent slopes of each domain associated to covariates?
4. Do baseline levels in one domain contribute to changes in the other two (covariance between latent intercepts and latent slopes across domains). We were particularly interested if baseline levels of WMH related to brain morphometric measures decline rates and cognitive decline, as well as if baseline levels of brain morphometric measures related to cognitive decline.
5. Are changes in one domain associated with changes in another (covariance between latent slopes)?

For our main objective, we employed trivariate LGCM to jointly analyse longitudinal trajectories and interrelations on a global level between total WMH, MTLV-ratio, and cognitive performance. Additionally, we employed two regional-level trivariate LGCMs involving frontal WMH and cortical volume, or posterior WMH and parietal cortical volume, respectively. We opted for complementary analysis of features in these analogous regions for the following reasons: First, to ensure comparability, avoiding a mixture of conceptually different regions. Second, similar to potential insights into underlying pathology by the anterior-posterior WMH distribution, atrophy in both frontal and parietal regions is driven by ageing, but parietal regions were also shown to be vulnerable to, e.g. AD-pathological change^14,15,85^. Third, by distinguishing anterior-posterior processes, we sought to capture a frontal, more vascular-dependent, and a posterior, more AD-pathology-dependent pathway to accelerated neurocognitive ageing. While frontal regions were linked more closely to executive functions, parietal regions appear to be more relevant for memory performance. We hence assumed that associations with PACC5—primarily reflecting memory, but also including executive components—may be stronger for parietal cortical volume changes and posterior WMH progression than for the same processes in frontal regions. We accounted for multiple comparisons by FDR correction for 12 cross-domain parameters of interest per model.

All latent intercepts and latent slopes were adjusted for the effects of age, sex, and years of education. Moreover, latent intercepts and latent slopes of WMH and brain morphometric measures were corrected for TICV.

Before fitting the trivariate models, we fitted separate univariate LGCMs to examine the possibility of nonlinear slopes for each feature per domain. For each domain, we compared a model with a linear slope only against a model including both linear and quadratic slopes using the Satorra-Bentler scaled χ² difference test. Linear slopes denoted rates of change per year, while quadratic slopes would indicate acceleration of changes. Only for MTLV-ratio, including a quadratic slope increased model fit significantly (*Δχ2* = 27.503, *Δdf* = 8, *p* = 5.79 × 10^−4^; Supplementary Table 3). In all other dimensions, linear slopes sufficed. Therefore, the global model encompassing total WMH, MTLV-ratio, and PACC5 included a quadratic slope alongside the linear slope for MTLV-ratio, while regional models were fitted with linear slopes only on all dimensions.

Given the annual cognitive assessment, we additionally accounted for retest-related practice effects in the cognitive domain. We considered two conceptually equivalent approaches by comparing modelling of manifest-level and latent-level practice effects^86,87^. Although latent-level modelling would theoretically allow estimation of interindividual variability in practice effects, no such variability was observed here. Therefore, to adjust for population-level practice effects parsimoniously, practice was modelled as a fixed population-level offset at all manifest follow-ups.

Models were fitted using the robust maximum likelihood estimator (MLR), and missing data were handled using Full Information Maximum Likelihood Estimation (FIML) with Yuan-Bentler scaled test statistics. We ascertained the assumption of data missing at random via Little’s missing completely at random test (*χ2*(1466) = 1544.30, *p* = 0.076).

Before model fitting, we identified and removed outliers for WMH, brain morphometric measures, and PACC5 performance, separately for each assessment time point. Values exceeding the interquartile range by more than Q3 + 1.5 × IQR or below Q1 − 1.5 × IQR of the median were considered outliers and set to missing in the respective time point. The IQR-based criterion follows distribution-free outlier detection^88^, which has been shown to provide a robust compromise between sensitivity and false-positive control across a wide range of non-Gaussian distributions^89,90^. The number of detected outliers per variable and time point for each model is reported in the corresponding supplementary tables detailing model specifications.

Model fit was evaluated by the *χ*² test, robust Comparative Fit Index (CFI), robust root mean square error of approximation (RMSEA) alongside its 90% confidence interval, and standardized root mean square residuals (SRMR). Good model fit was defined as CFI ≥ 0.97, RMSEA ≤ 0.05, SRMR ≤ 0.05^91^. We reported estimates from the fully standardized solution to present comparable, unit-independent effect estimates. We report full model information including the raw estimates in supplements. The threshold for *p* values was set to *p* ≤ 0.05.

To facilitate the assessment of the number of individuals progressing or regressing in either domain, we extracted regression-based factor score estimates of latent slopes for each individual and report the percentages of individuals with negative or positive factor scores.

##### Unique contributions of WMH and MTLV-ratio rates of change to cognitive ageing

We aimed to examine the independent contribution of changes in WMH and MTLV-ratio to cognitive change, as the covariance of two latent variables within the LGCM does not account for the effect of the other latent variables. To facilitate interpretation, we divided our analysis into two parts. First, we estimated latent intercepts and latent slopes (see Latent Growth Curve Modelling) and extracted regression-based factor score estimates for each individual. Second, we used robust multiple linear regression to examine the effect of WMH and MTLV-ratio latent slopes on the latent slopes of PACC5 performance, adjusting also for all latent intercepts. We tested a model with only additive effects against a model including an interaction effect of the linear latent slopes of WMH and MTLV-ratio to examine possible synergistic effects of both pathological processes on cognitive changes. For model comparison, we used *F*-test.

##### Supplementary analysis I: Assessing contributions of shared risk factors

Relationships between the neurocognitive domains of interest were proposed to possibly stem from shared risk factors, such as cardiovascular risks^19^. Moreover, in the context of Alzheimer’s disease (AD), brain atrophy and WMH progression might be entangled due to the accumulation of AD biomarkers^22^, while alternative hypotheses claim they are entirely distinct and independent epiphenomena^92,93^. In supplementary analyses, we therefore tested separate LGCMs which additionally corrected for (a) vascular risk, (b) APOE-ε4 carriership and plasma Aβ42/40^94^, (c) plasma pTau181^39^, and (d) plasma neurofilament light chain (Nfl)^39^. We examined whether controlling for these common underlying risk factors would alter the covariant structure between neurocognitive domains.

##### Supplementary analysis II: Differences between converting and cognitively stable individuals

To validate our multi-domain model, we examined in a supplementary analysis whether latent factor scores in the three neurocognitive domains of interest differed between individuals who stayed cognitively stable within the course of the study (*n* = 413) and those who converted to mild cognitive impairment (MCI) or dementia (*n* = 93 [*n*_converted to MCI_ = 86, n_converted to MCI and subsequent dementia_ = 7]). Conversion status in all individuals was assessed up to 5 years and 4 months after inclusion in the study (mean follow-up time: 5.41 years; *n* = 506; 37 individuals exceeded this range). Information on clinical progression to MCI or dementia was assessed for cognitively unimpaired individuals up until April 2023^95^.

Due to the small sample of converted individuals, we refrained from using grouped LGCM to analyse these differences. Instead, we extracted regression-based factor scores of the latent intercepts and latent slopes and compared them via two-sided Mann-Whitney-U tests, with $r=\,\frac{Z}{\sqrt{N}}$ as effect size. Individuals who converted were generally older (*U* = 23836, *p* = 2.73 × 10^−4^, *r* = 0.162 [0.08, 0.24]), had higher cardiovascular risk (*U* = 23055, *p* = 0.002, *r* = 0.140 [0.05, 0.23]), lower baseline plasma Aβ42/40 ratios (*U* = 15143, *p* = 1.78 × 10^−4^, *r* = 0.140 [0.05, 0.23]), higher plasma pTau181 levels (*U* = 17285, *p* = 1.09 × 10^−4^, *r* = 0.186 [0.09, 0.27]), and higher plasma neurofilament light chain levels (NfL; *U* = 20005, *p* = 0.017, *r* = 0.140 [0.09, 0.27]) than cognitively stable individuals. There were no significant differences between converting and cognitively stable individuals in terms of sex distribution or education. Differences across groups in modifiable lifestyle factors are detailed in Supplementary Table 2.

#### Domain-specific and domain-general contributors to neurocognitive changes

Using the extracted factor scores, we examined domain-specific relationships with modifiable lifestyle factors and personality traits via FDR-corrected Spearman’s correlation that accounted for sex, age, years of education, and TICV. We also report unaccounted correlations in supplements. In order to give an estimate of how much these contributing factors may explain in the variability of factor scores, we leveraged robust multiple linear regression.

Based on research suggestions for brain maintenance^9^, we additionally quantified maintenance more explicitly using the predicted values from the multiple regression model (see Unique contributions of WMH and MTLV-ratio rates of change to cognitive ageing), which are thought to reflect the brain-structure–related component of cognitive ageing. The index describes individual differences in cognitive changes that are shared with structural brain changes. Higher individual predicted values hence indicate preserved cognitive functioning related to preserved brain integrity, consistent with more successful brain maintenance. We then correlated this brain maintenance index with modifiable lifestyle factors and personality traits to identify domain-general contributors to brain maintenance, and compared differences in brain maintenance between cognitively stable vs. converting individuals. To validate and assess the biological relevance of the brain maintenance index, we assessed whether it related to an independent molecular marker of biological ageing pace. More specifically, we correlated it with the baseline DunedinPACE^96^ epigenetic-ageing score derived from DNA-methylation profiling available in a subsample of participants (*n* = 502).

DNA methylation was assessed in blood using the Illumina MethylationEPIC BeadChip, and arrays were scanned using the Illumina iScan system. The methylation data were processed using the *R* package *meffil* (v1.3.8)^97^. Quality control followed the default settings implemented in the package. Functional normalization was applied using 20 principal components, with batch information included as a fixed effect. The normalized beta values were subsequently used to calculate DunedinPACE using the *R* package *dnaMethyAge* (v0.2.0). Partial Spearman’s correlations were computed between the maintenance index and DunedinPACE, adjusting for age, sex, years of education, and TICV.

##### Supplementary analysis III: Differences between individuals with high and low education

As lifestyle could counterbalance the risk for cognitive decline and brain integrity in face of low education or socioeconomic status^64^, we explored whether associations between lifestyle factors and personality traits and each neurocognitive domain differed between individuals with high and low education—a key factor for preserving cognition and lower dementia risk^10,51^. We divided the sample into individuals with lower vs. higher education based on a median split (*median*_*years of education*_ = 14; lower education: *n* = 273, *mean*_*years of education*_ = 12.3 ± 1.29; higher education: *n* = 270, *mean*_*years of education*_ = 17.4 ± 1.60). To ensure that education-related variability in latent slopes of WMH, MTLV-ratio, and PACC5 performance was retained, we retrieved regression-based factor scores from a trivariate LGCM which did not include years of education. We then computed the aforementioned FDR-corrected Spearman’s correlations between personality and lifestyle factors and domain-specific latent slopes in lower and higher education groups separately, accounting for sex, age, and TICV.

#### Software

We carried out all analyses in *R* (v4.2.3) using RStudio (v1.3.1073). We modelled trivariate LGCM using *lavaan* (v0.6-16). We used Mann-Whitney-U tests in *rstatix* (v0.7.2), partial correlation in *ppcor* (v1.1), and robust multiple linear regression in *robustbase* (v0.99.0). We created figures using *ggplot2* (v3.4.2) and *semPlot* (v1.1.6).

### Reporting summary

Further information on research design is available in the Nature Portfolio Reporting Summary linked to this article.

## Supplementary information

Supplementary Information

Reporting Summary

Transparent Peer Review file

## Source data

Source Data

## Data Availability

The raw data collected in the study DELCODE—DZNE-Longitudinal Cognitive Impairment and Dementia Study (BN012) cannot be made openly available without violation of the data protection concept of the DZNE. Access to the relevant study data can be obtained by submitting an application to the Clinical Research Platform of the DZNE. The template for the application for the submission of data and biomaterial samples is available on the DZNE homepage (https://www.dzne.de/en/research/research-areas/clinical-research/for-researchers/). The expected timeframe for response to access requests is 1 month. Access will be granted for 10 years. Source data are provided with this paper.

## Code availability

The R code implementing the trivariate latent growth curve model (lavaan syntax), factor-score extraction, and robust regression for the brain maintenance index is openly available at https://github.com/neuroprognosis/brain-maintenance-lgcm. The repository contains a synthetic example dataset reproducing the analysis pipeline end-to-end so that the framework is usable on alternative cohorts without access to DELCODE raw data.
