# Supplementary material for: Joint trajectories of brain atrophy, white matter hyperintensities and cognition quantify brain maintenance
Source: Nat Commun. 2026 Jul 4;17:5846. doi: 10.1038/s41467-026-74957-2 (PMC13332871; doi:10.1038/s41467-026-74957-2)
Supplement: Supplementary file 1 — Supplementary Information [file 41467_2026_74957_MOESM1_ESM.pdf]

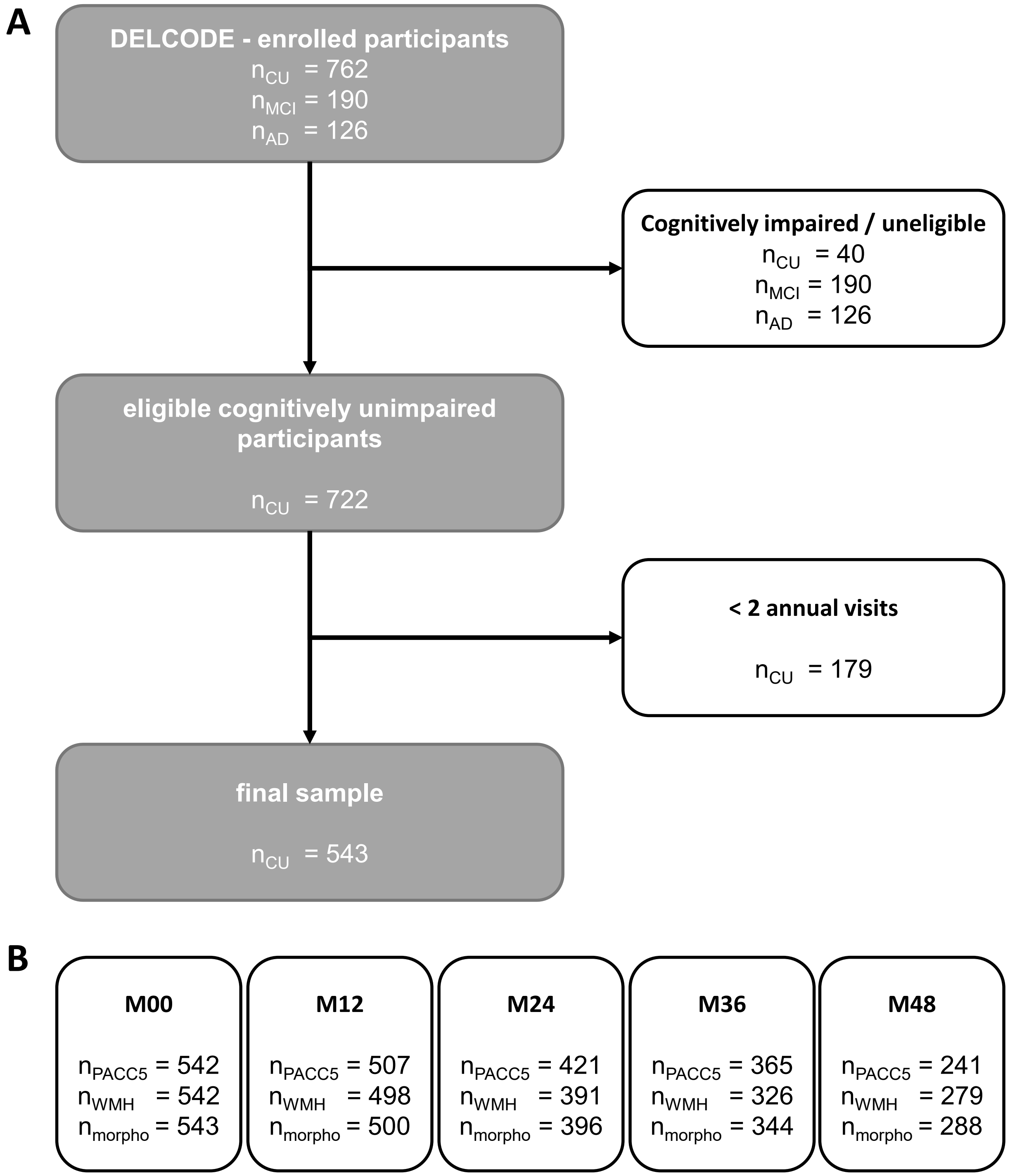

Supplementary Figure 1. Sample flow chart. (A) Flow chart of included, eligible, cognitively unimpaired individuals with at least two annual visits used for the analysis (n = 543). CU = cognitively unimpaired; MCI = mild cognitive impairment; AD =dementia of the Alzheimer type. **(B)** Available data per domain of interest per annual assessment time point: Cognition assessed by PACC5; white matter hyperintensities (WMH) encompassing total volumes and regional volumes in frontal and posterior regions; brain morphometric data (morpho) encompassing medial temporal lobe to ventricle ratio (MTLV-ratio), frontal and parietal cortical volume.

Supplementary Table 1. Overview of modifiable lifestyle factors and personality traits used as contributors to trajectories of each neurocognitive domain.

Possible brain maintenance factor	Operationalized by	Description/Details	Score
**Total cardiovascular risk score**	information of baseline medical records	• Assessed at baseline<br>• Equally weighted score according to former or current smoking, presence of obesity, hyperlipidaemia, hypertension, diabetes<br>• Each condition coded as present or absent<br>• sum of present risk factors was corrected for amount of available information ($n_{available}$ = 543)	• 0.0 to 1.0<br>• Higher values reflect higher cardiovascular risk
**Late life depressive symptoms**	15-item German version of geriatric depression scale (GDS) [1]	• Assessed annually<br>• 5 to 10 points correspond to mild to moderate depressive symptoms<br>• 11 to 15 points denote severe depressive symptoms<br>• 2 % missing at baseline ($n_{available}$ = 532)	• 0.0 to 15.0<br>• Higher values reflect higher depressive symptoms
**Mediterranean diet**	European Prospective Investigation of Cancer semi-quantitative food frequency questionnaire [2]	• Assessed at baseline<br>• 148 items queried for the last year<br>• adherence to Mediterranean diet according [3]<br>• 31 % missing at baseline ($n_{available}$ = 373)	• higher values denote stronger adherence to Mediterranean diet
**Physical activity**	German version of physical activity scale for the elderly (PASE) [4,5]	• assessed annually<br>• queried physical activity during the week prior each visit<br>• individuals report time spent sitting, by foot, with different activities of mild to severe intensities<br>• CAVE: modified scoring procedure that did not include the number of hours spent with an activity, as the reported hours by DELCODE participants proved to be implausible<br>• 4 % missing at baseline ($n_{available}$ = 521)	• higher values denote higher physical activity
**Sleep quality**	Pittsburgh Sleep Quality Index (PSQI) [6]	• assessed annually<br>• queried four weeks prior each visit<br>• total score used as proxy for sleep quality<br>• 10 % missing at baseline ($n_{available}$ = 532)	• 0.0 to 21.0<br>• higher values reflect lower sleep quality

**Social network**	6-item German version of Lubben Social Network Scale (LSNS) [7]	• assessed annually<br>• queries amount of family members and friends, with whom participants were in regular contact or received support from<br>• 4 % missing at baseline ($n_{available}$ = 489)	• 0.0 to 30.0<br>• scores below 12 indicate social isolation.
**Lifetime experiences**	Lifetime experiences questionnaire (LEQ) [8]	• LEQ measures of young adulthood and midlife assessed at baseline<br>• LEQ for late life assessed at each visit<br>• queries complex cognitive engagement during three life periods: young adulthood (y: 13-30 years), midlife (m: 30-65 years) and late life (h: ≥ 65 years or from retirement onward)<br>• differentiates between specific and non-specific activities assessed for each period<br>○ Specific activities refer to life-period-specific activities (education, employment)<br>○ non-specific activities refer to activities that can span all life periods (leisure time activities, e.g., reading, artistic endeavours)<br>• missing at baseline<br>○ Specific: LEQ_y = 1% ($n_{available}$ = 535); LEQ_m =5% ($n_{available}$ = 518); LEQ_h = 14% ($n_{available}$ = 467)<br>○ Non-specific: LEQ_y = 2% ($n_{available}$ = 532); LEQ_m =2% ($n_{available}$ = 534); LEQ_h = 24% ($n_{available}$ = 414)	• higher values reflect higher cognitive engagement
**Personality traits**	Big Five Inventory BFI-10 [9]	• assessed at baseline<br>• assessed personality traits openness, conscientiousness, extraversion, agreeableness, and neuroticism according to the five-factor model<br>• each trait assessed with 2 items which were averaged for each dimension<br>• 1 % missing at baseline ($n_{available}$ = 536)	• range per each personality trait from 1.0 – 5.0<br>• higher values reflect higher manifestation of trait

Supplementary Table 2. Descriptive statistics of personality traits and modifiable lifestyle factors at baseline in the total sample and comparing CU-stable and CU-converted individuals.

	Total	CU-stable	CU-converted	$p_{\text{CU-stable vs. CU-converted}}$	$r_{\text{CU-stable vs. CU-converted}}$
**cardiovascular risk**	**0.31 ± 0.22**	0.29 ± 0.21	0.38 ± 0.24	0.002	0.140 [0.05, 0.23]
**Personality**					
Openness	**3.57 ± 0.89**	3.59 ± 0.89	3.42 ± 0.89	0.088	0.076 [0.01, 0.16]
Conscientiousness	**3.89 ± 0.80**	3.88 ± 0.79	3.97 ± 0.85	0.293	0.047 [0.00, 0.13]
Extraversion	**3.25 ± 0.98**	3.23 ± 0.98	3.29 ± 0.99	0.592	0.024 [0.00, 0.11]
Agreeableness	**3.25 ± 0.78**	3.25 ± 0.78	3.31 ± 0.76	0.577	0.025 [0.00, 0.11]
Neuroticism	**2.98 ± 0.98**	2.92 ± 0.99	3.18 ± 0.95	0.020	0.104 [0.02, 0.19]
**GDS**	**1.45 ± 1.87**	1.27 ± 1.73	2.20 ± 2.31	7.54×10^{-6}	0.201 [0.12, 0.28]
**MeDi**	**4.60 ± 1.58**	4.65 ± 1.60	4.25 ± 1.61	0.111	0.087 [0.01, 0.18]
**PASE**	**31.78 ± 12.49**	32.20 ± 12.70	30.70 ± 11.70	0.384	0.040 [0.00, 0.13]
**PSQI**	**5.47 ± 3.31**	5.36 ± 3.19	5.71 ± 3.67	0.502	0.032 [0.00, 0.12]
**LSNS**	**22.35 ± 5.07**	22.30 ± 5.02	22.50 ± 5.36	0.828	0.010 [0.00, 0.10]
**Lifetime experiences**					
LEQy specific	**18.51 ± 5.31**	18.60 ± 5.32	18.40 ± 5.38	0.608	0.023 [0.00, 0.12]
LEQm specific	**32.30 ± 10.86**	32.60 ± 10.90	31.80 ± 10.90	0.592	0.024 [0.00, 0.11]
LEQh specific	**22.58 ± 5.14**	22.50 ± 5.24	22.40 ± 4.94	0.927	0.004 [0.00, 0.11]
LEQy nonspecific	**18.91 ± 4.97**	19.10 ± 5.09	18.10 ± 4.61	0.064	0.083 [0.01, 0.17]
LEQm nonspecific	**18.53 ± 4.70**	18.60 ± 4.65	18.00 ± 5.12	0.196	0.058 [0.00, 0.16]
LEQh nonspecific	**16.38 ± 4.49**	16.50 ± 4.53	15.90 ± 4.74	0.389	0.044 [0.00, 0.15]

Annotations. Mean ± standard deviation. Comparison of CU-stable vs. CU-converted via two-sided Mann-Whitney-U tests on the averaged lifestyle factors. Effect size r with 95% confidence interval. Personality traits were acquired via the BFI-10. GDS = geriatric depression scale. MeDi = Mediterranean diet. PASE = physical activity scale for the elderly. PSQI = Pittsburgh sleep quality index. LSNS = Lubben social network scale. LEQ = lifetime experiences questionnaire, y = young (13-30 years), m = middle-age (30-65 years), h = high age (≥ 65 years or from retirement onward).

Supplementary Table 3. Satorra-Bentler scaled χ2 difference tests (one-sided) comparing model with linear slope only vs. model with linear and quadratic slope in each neurocognitive domain of interest.

		Δ χ2	Δdf	p
**Cognition**	**PACC5** [a]	9.38	6	0.153
**Brain morpho-metric measure**	**MTLV-ratio** [b]	27.50	8	5.79×10^{-4}
	**Frontal cortical volume**	12.73	8	0.122
	**Parietal cortical volume**	12.37	8	0.136
**WMH**	**Total** [c]	0.93	7	0.996
	**Frontal** [d]	13.42	7	0.063
	**Posterior**	7.91	8	0.443

Annotations. [a] including a quadratic slope led to model convergence issues (negative quadratic slope variance), which was resolved by specifying uncorrelated linear and quadratic slope. [b] significant mean quadratic change (B = -0.439, Z = -3.881, p < 0.001) with trend wise significant variance (B = 0.909, Z = 1.752, p = 0.080). [c] including a quadratic slope led to model convergence issues (negative quadratic slope variance), which was resolved by fixing the quadratic slope variance to a small positive number (0.0001). [d] including a quadratic slope led to model convergence issues (negative quadratic slope variance), which was resolved by specifying uncorrelated linear and quadratic slope.

Supplementary Table 4. Model details of the main trivariate latent growth curve model between PACC5, total WMH volumes, and MTLV-ratio. Models included covariates age, sex, years of education, and total intracranial volume (TICV). Model fit: $\chi^2(135) = 155.06$, $p = 0.114$; *CFI* = 0.998; *RMSEA* = 0.022 [0.000, 0.038]; *SRMR* = 0.018; *Yuan-Bentler scaling factor* = 1.081. Number of detected outliers per variable and time point (M00/M12/M24/M36/M48) was as follows: $n_{Outliers\ PACC5}$ = 8/4/5/4/2, $n_{Outliers\ WMH}$ = 5/6/4/1/6, $n_{Outliers\ MTLV\text{-}ratio}$ = 27/26/17/16/7. P-values below machine precision are reported as $< 2.2\times10^{-16}$.

				Estimate	SE	95% CI	Std. Estimate	Z	p
Association with Demographics	Intercept cognition	~	Age	-0.315	0.033	-0.38, -0.25	-0.398	-9.646	< 2.2×10^-16
		~	Sex	0.282	0.031	0.22, 0.34	0.356	9.013	< 2.2×10^-16
		~	Years of Education	0.204	0.032	0.14, 0.27	0.257	6.337	2.34×10^-10
	Slope cognition	~	Age	-0.042	0.009	-0.06, -0.02	-0.367	-4.783	1.73×10^-06
		~	Sex	-0.008	0.009	-0.02, 0.01	-0.068	-0.908	0.364
		~	Years of Education	0.014	0.009	-0.00, 0.03	0.120	1.581	0.114
	Intercept WMH	~	Age	0.355	0.038	0.28, 0.43	0.371	9.250	< 2.2×10^-16
		~	Sex	0.159	0.053	0.05, 0.26	0.166	2.973	0.003
		~	Years of Education	-0.065	0.039	-0.14, 0.01	-0.068	-1.674	0.094
		~	TICV	0.249	0.051	0.15, 0.35	0.260	4.883	1.04×10^-06
	Slope WMH	~	Age	-0.006	0.004	-0.01, 0.00	-0.102	-1.462	0.144
		~	Sex	0.002	0.006	-0.01, 0.01	0.026	0.272	0.786
		~	Years of Education	-0.003	0.004	-0.01, 0.01	-0.044	-0.660	0.509
		~	TICV	-0.003	0.005	-0.01, 0.01	-0.050	-0.639	0.523
	Intercept brain	~	Age	-0.353	0.028	-0.41, -0.30	-0.495	-12.609	< 2.2×10^-16
		~	Sex	0.104	0.034	0.04, 0.17	0.146	3.076	0.002
		~	Years of Education	0.074	0.027	0.02, 0.13	0.103	2.720	0.007
		~	TICV	-0.138	0.031	-0.20, -0.08	-0.193	-4.378	1.20×10^-05
	Linear slope brain	~	Age	-0.038	0.006	-0.05, -0.03	-0.434	-6.241	4.35×10^-10
		~	Sex	0.000	0.007	-0.01, 0.01	0.004	0.044	0.965
		~	Years of Education	-0.005	0.006	-0.02, 0.01	-0.055	-0.815	0.415

				Estimate	SE	95% CI	Std. Estimate	Z	p
		~	TICV	-0.009	0.008	-0.02, 0.01	-0.106	-1.180	0.238
	Quadratic slope brain	~	Age	0.002	0.001	-0.00, 0.00	0.145	1.241	0.215
		~	Sex	0.002	0.002	-0.00, 0.01	0.141	0.969	0.332
		~	Years of Education	0.003	0.001	0.00, 0.01	0.272	2.291	0.022
		~	TICV	-0.001	0.002	-0.00, 0.00	-0.091	-0.717	0.473
**Association between latent variables**	Intercept cognition	~~	Slope cognition	0.015	0.007	0.00, 0.03	0.226	2.100	0.036
		~~	Intercept WMH	-0.033	0.023	-0.08, 0.01	-0.062	-1.423	0.155
		~~	Slope WMH	0.002	0.003	-0.00, 0.01	0.045	0.573	0.566
		~~	Intercept brain	0.052	0.018	0.02, 0.09	0.151	2.877	0.004
		~~	Linear slope brain	0.008	0.004	-0.00, 0.02	0.168	1.900	0.057
		~~	Quadratic slope brain	0.000	0.001	-0.00, 0.00	-0.058	-0.430	0.667
	Slope cognition	~~	Intercept WMH	-0.005	0.007	-0.02, 0.01	-0.053	-0.648	0.517
		~~	Slope WMH	-0.001	0.001	-0.00, -0.00	-0.208	-2.010	0.044
		~~	Intercept brain	0.015	0.005	0.00, 0.03	0.259	2.838	0.005
		~~	Linear slope brain	0.003	0.001	0.00, 0.01	0.354	2.439	0.015
		~~	Quadratic slope brain	0.000	0.000	-0.00, 0.00	0.224	1.070	0.285
	Intercept WMH	~~	Slope WMH	-0.003	0.004	-0.01, 0.00	-0.064	-0.823	0.410
		~~	Intercept brain	-0.066	0.021	-0.11, -0.03	-0.136	-3.195	0.001
		~~	Linear slope brain	-0.013	0.005	-0.02, -0.00	-0.186	-2.734	0.006
		~~	Quadratic slope brain	0.000	0.001	-0.00, 0.00	0.029	0.256	0.798
	Slope WMH	~~	Intercept brain	-0.002	0.002	-0.01, 0.00	-0.045	-0.613	0.540
		~~	Linear slope brain	-0.001	0.000	-0.00, 0.00	-0.130	-1.450	0.147
		~~	Quadratic slope brain	0.000	0.000	-0.00, 0.00	-0.084	-0.629	0.529
	Intercept brain	~~	Linear slope brain	0.018	0.004	0.01, 0.03	0.413	4.348	1.37×10^-05
		~~	Quadratic slope brain	0.002	0.001	-0.00, 0.00	0.245	1.603	0.109
	Linear slope brain	~~	Quadratic slope brain	0.000	0.000	-0.00, 0.00	-0.354	-1.061	0.289

				Estimate	SE	95% CI	Std. Estimate	Z	p
Association covariates	Age	~~	Sex	-0.194	0.042	-0.28, -0.11	-0.194	-4.662	3.13×10^-06
		~~	TICV	0.103	0.041	0.02, 0.18	0.103	2.497	0.013
		~~	Years of Education	-0.111	0.046	-0.20, -0.02	-0.111	-2.430	0.015
	Sex	~~	TICV	-0.665	0.020	-0.70, -0.63	-0.665	-33.180	< 2.2×10^-16
		~~	Years of Education	-0.227	0.039	-0.30, -0.15	-0.227	-5.791	7.00×10^-09
	Years of Education	~~	TICV	0.251	0.039	0.18, 0.33	0.251	6.514	7.32×10^-11

Supplementary Table 5. Model details of the trivariate latent growth curve model between PACC5, frontal WMH volumes, and frontal cortical volumes. Models included covariates age, sex, years of education, and total intracranial volume (TICV). Model fit: $\chi^2(147) = 208.53$, p = 0.001; *CFI* = 0.993; TLI = 0.992; *RMSEA* = 0.041 [0.026, 0.054]; *SRMR* = 0.025; Yuan-Bentler scaling factor = 1.15. Number of detected outliers per variable and time point (M00/M12/M24/M36/M48) was as follows: $n_{\text{Outliers PACC5}}$ = 8/4/5/4/2, $n_{\text{Outliers frtonal WMH}}$ = 5/5/8/5/8, $n_{\text{Outliers frontal volume}}$ = 5/7/6/8/5 P-values below machine precision are reported as $< 2.2\times10^{-16}$. Frontal WMH significantly increased with substantial interindividual variability (intercept frontal WMH slope: B = 1.269, Z = 19.45, p $< 2.2\times10^{-16}$; variance frontal WMH slope: B = 0.968, Z = 5.375, p = 7.66×10^{-8}). Frontal cortical volume decreased with substantial interindividual variability (intercept frontal cortical volume slope: B = -0.624, Z = -8.75, p < 2.2×10^{-16}; variance frontal cortical volume slope: B = 0.912, Z = 5.176, p = 2.26×10^{-7}). Higher baseline PACC5 performance was linked to lower frontal volume loss (intercept PACC5 ~ slope frontal volume: $cov_{Standardized}$ = 0.190, Z = 2.066, p = 0.039, p_{FDR} = 0.378), however this association did not survive FDR-correction.

				Estimate	SE	95% CI	Std. Estimate	Z	p
**Association with Demographics**	Intercept cognition	~	Age	-0.316	0.033	-0.38, -0.25	-0.399	-9.613	< 2.2×10^-16
		~	Sex	0.280	0.031	0.22, 0.34	0.354	8.926	< 2.2×10^-16
		~	Years of Education	0.202	0.032	0.14, 0.26	0.255	6.257	3.93×10^-10
	Slope cognition	~	Age	-0.042	0.009	-0.06, -0.02	-0.372	-4.741	2.12×10^-06
		~	Sex	-0.005	0.009	-0.02, 0.01	-0.048	-0.626	0.531
		~	Years of Education	0.015	0.009	-0.00, 0.03	0.131	1.713	0.087
	Intercept WMH	~	Age	0.333	0.038	0.26, 0.41	0.354	8.683	< 2.2×10^-16
		~	Sex	0.161	0.052	0.06, 0.26	0.171	3.068	0.002
		~	Years of Education	-0.072	0.038	-0.15, 0.00	-0.076	-1.877	0.060
		~	TICV	0.244	0.051	0.14, 0.34	0.259	4.816	1.46×10^-06
	Slope WMH	~	Age	-0.005	0.004	-0.01, 0.00	-0.098	-1.335	0.182
		~	Sex	0.002	0.005	-0.01, 0.01	0.034	0.375	0.708
		~	Years of Education	0.000	0.004	-0.01, 0.01	0.003	0.047	0.962
		~	TICV	-0.006	0.004	-0.01, 0.00	-0.114	-1.453	0.146
	Intercept brain	~	Age	-0.170	0.023	-0.21, -0.13	-0.179	-7.483	7.26×10^-14
		~	Sex	0.126	0.031	0.07, 0.19	0.133	4.079	4.52×10^-05
		~	Years of Education	0.047	0.022	0.00, 0.09	0.049	2.127	0.033

				Estimate	SE	95% CI	Std. Estimate	Z	p
		~	TICV	0.878	0.032	0.82, 0.94	0.925	27.444	< 2.2×10^-16
	Slope brain	~	Age	-0.010	0.003	-0.02, -0.00	-0.242	-2.978	0.003
		~	Sex	0.001	0.004	-0.01, 0.01	0.030	0.301	0.764
		~	Years of Education	0.001	0.003	-0.01, 0.01	0.018	0.256	0.798
		~	TICV	-0.005	0.004	-0.01, 0.00	-0.122	-1.256	0.209
**Association between latent variables**	Intercept cognition	~~	Slope cognition	0.013	0.007	-0.00, 0.03	0.204	1.826	0.068
		~~	Intercept WMH	-0.037	0.025	-0.09, 0.01	-0.070	-1.507	0.132
		~~	Slope WMH	0.001	0.003	-0.00, 0.01	0.025	0.304	0.761
		~~	Intercept brain	0.015	0.014	-0.01, 0.04	0.051	1.071	0.284
		~~	Slope brain	0.005	0.002	0.00, 0.01	0.190	2.066	0.039
	Slope cognition	~~	Intercept WMH	-0.004	0.007	-0.02, 0.01	-0.049	-0.576	0.564
		~~	Slope WMH	-0.001	0.001	-0.00, 0.00	-0.147	-1.279	0.201
		~~	Intercept brain	0.002	0.004	-0.01, 0.01	0.049	0.608	0.543
		~~	Slope brain	0.000	0.001	-0.00, 0.00	-0.049	-0.326	0.744
	Intercept WMH	~~	Slope WMH	-0.010	0.004	-0.02, -0.00	-0.202	-2.593	0.010
		~~	Intercept brain	-0.033	0.018	-0.07, 0.00	-0.080	-1.858	0.063
		~~	Slope brain	0.000	0.002	-0.01, 0.00	-0.006	-0.079	0.937
	Slope WMH	~~	Intercept brain	-0.001	0.002	-0.00, 0.00	-0.050	-0.819	0.413
		~~	Slope brain	0.000	0.000	-0.00, 0.00	0.045	0.412	0.680
	Intercept brain	~~	Slope brain	0.002	0.002	-0.00, 0.01	0.120	1.473	0.141
**Association covariates**	Age	~~	Sex	-0.194	0.042	-0.28, -0.11	-0.194	-4.662	3.13×10^-06
		~~	TICV	0.103	0.041	0.02, 0.18	0.103	2.497	0.013
		~~	Years of Education	-0.111	0.046	-0.20, -0.02	-0.111	-2.430	0.015
	Sex	~~	TICV	-0.665	0.020	-0.70, -0.63	-0.665	-33.180	< 2.2×10^-16
		~~	Years of Education	-0.227	0.039	-0.30, -0.15	-0.227	-5.791	7.00×10^-09
	Years of Education	~~	TICV	0.251	0.039	0.18, 0.33	0.251	6.514	7.32×10^-11

Supplementary Table 6. Model details of the trivariate latent growth curve model between PACC5, posterior WMH volumes, and parietal cortical volumes. Models included covariates age, sex, years of education, and total intracranial volume (TICV). Model fit: $\chi^2(147) = 148.00$, $p = 0.461$; $CFI = 1.00$; TLI = 0.998; $RMSEA = 0.000$ [0.000, 0.025]; $SRMR = 0.024$; Yuan-Bentler scaling factor = 1.114. Number of detected outliers per variable and time point (M00/M12/M24/M36/M48) was as follows: $n_{\text{Outliers PACC5}}$ = 8/4/5/4/2, $n_{\text{Outliers posterior WMH}}$ = 3/5/5/2/2, $n_{\text{Outliers parietal volume}}$ = 5/2/4/3/4. P-values below machine precision are reported as $< 2.2\times10^{-16}$. Posterior WMH significantly increased with substantial interindividual variability (intercept posterior WMH slope: B = 0.712, Z = 12.320, $< 2.2\times10^{-16}$; variance posterior WMH slope: B = 0.986, Z = 6.355, $p < 2.2\times10^{-16}$). Parietal cortical volume decreased with substantial interindividual variability (intercept parietal cortical volume slope: B = -0.968, Z = -12.043, $p < 2.2\times10^{-16}$; variance parietal cortical volume slope: B = 0.765, Z = 2.780, p = 0.005). We found that higher baseline PACC5 performance associated with lower parietal cortical volume loss (*intercept PACC5 ~ slope parietal volume*: $cov_{Standardized} = 0.249$, $Z = 2.205$, $p = 0.027$, $p_{FDR} = 0.324$), which however did not survive FDR-correction.

				Estimate	SE	95% CI	Std. Estimate	Z	p
Association with Demographics	Intercept cognition	~	Age	-0.316	0.033	-0.38, -0.25	-0.399	-9.631	< 2.2×10^-16
		~	Sex	0.281	0.031	0.22, 0.34	0.355	8.956	< 2.2×10^-16
		~	Years of Education	0.202	0.032	0.14, 0.26	0.255	6.257	3.92×10^-10
	Slope cognition	~	Age	-0.041	0.009	-0.06, -0.02	-0.372	-4.748	2.06×10^-06
		~	Sex	-0.006	0.009	-0.02, 0.01	-0.057	-0.751	0.453
		~	Years of Education	0.014	0.009	-0.00, 0.03	0.127	1.649	0.099
	Intercept WMH	~	Age	0.300	0.036	0.23, 0.37	0.321	8.217	2.22×10^-16
		~	Sex	0.048	0.053	-0.06, 0.15	0.051	0.904	0.366
		~	Years of Education	-0.077	0.038	-0.15, -0.00	-0.083	-2.048	0.041
		~	TICV	0.226	0.051	0.13, 0.33	0.242	4.402	1.07×10^-05
	Slope WMH	~	Age	-0.001	0.005	-0.01, 0.01	-0.008	-0.143	0.886
		~	Sex	0.011	0.006	-0.00, 0.02	0.132	1.695	0.090
		~	Years of Education	-0.005	0.005	-0.01, 0.00	-0.056	-1.004	0.315
		~	TICV	0.008	0.006	-0.00, 0.02	0.101	1.431	0.152
	Intercept brain	~	Age	-0.194	0.025	-0.24, -0.14	-0.202	-7.601	2.93×10^-14
		~	Sex	0.145	0.035	0.08, 0.21	0.151	4.202	2.65×10^-05
		~	Years of Education	0.012	0.027	-0.04, 0.07	0.013	0.450	0.653

				Estimate	SE	95% CI	Std. Estimate	Z	p
		~	TICV	0.845	0.036	0.77, 0.91	0.881	23.732	< 2.2×10^-16
	Slope brain	~	Age	-0.013	0.003	-0.02, -0.01	-0.391	-4.531	5.88×10^-06
		~	Sex	0.002	0.004	-0.01, 0.01	0.073	0.642	0.521
		~	Years of Education	0.002	0.003	-0.00, 0.01	0.046	0.582	0.561
		~	TICV	-0.006	0.004	-0.01, 0.00	-0.182	-1.717	0.086
**Association between latent variables**	Intercept cognition	~~	Slope cognition	0.013	0.007	-0.00, 0.03	0.207	1.851	0.064
		~~	Intercept WMH	-0.021	0.024	-0.07, 0.02	-0.040	-0.899	0.369
		~~	Slope WMH	0.004	0.003	-0.00, 0.01	0.072	1.039	0.299
		~~	Intercept brain	0.012	0.018	-0.02, 0.05	0.035	0.700	0.484
		~~	Slope brain	0.005	0.002	0.00, 0.01	0.249	2.205	0.027
	Slope cognition	~~	Intercept WMH	-0.011	0.007	-0.03, 0.00	-0.126	-1.491	0.136
		~~	Slope WMH	0.000	0.001	-0.00, 0.00	0.006	0.060	0.952
		~~	Intercept brain	0.008	0.005	-0.00, 0.02	0.139	1.693	0.091
		~~	Slope brain	0.001	0.001	-0.00, 0.00	0.262	1.418	0.156
	Intercept WMH	~~	Slope WMH	-0.004	0.005	-0.01, 0.01	-0.051	-0.757	0.449
		~~	Intercept brain	0.005	0.023	-0.04, 0.05	0.010	0.227	0.820
		~~	Slope brain	-0.002	0.002	-0.01, 0.00	-0.095	-1.010	0.313
	Slope WMH	~~	Intercept brain	0.002	0.003	-0.00, 0.01	0.051	0.889	0.374
		~~	Slope brain	0.001	0.000	-0.00, 0.00	0.280	1.791	0.073
	Intercept brain	~~	Slope brain	0.003	0.002	0.00, 0.01	0.207	2.206	0.027
**Association covariates**	Age	~~	Sex	-0.194	0.042	-0.28, -0.11	-0.194	-4.662	3.13×10^-06
		~~	TICV	0.103	0.041	0.02, 0.18	0.103	2.497	0.013
		~~	Years of Education	-0.111	0.046	-0.20, -0.02	-0.111	-2.430	0.015
	Sex	~~	TICV	-0.665	0.020	-0.70, -0.63	-0.665	-33.180	< 2.2×10^-16
		~~	Years of Education	-0.227	0.039	-0.30, -0.15	-0.227	-5.791	7.00×10^-09
	Years of Education	~~	TICV	0.251	0.039	0.18, 0.33	0.251	6.514	7.32×10^-11

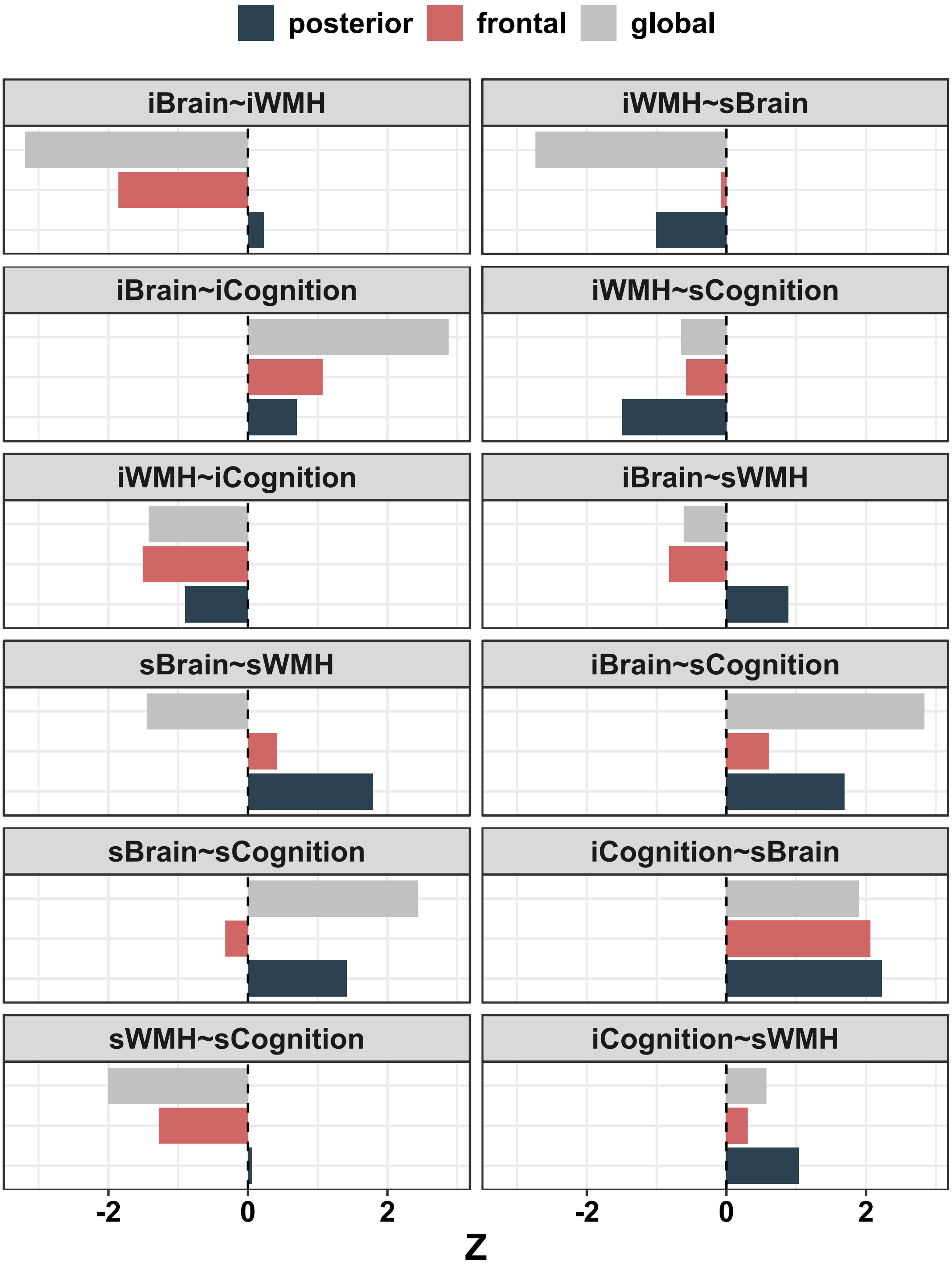

Supplementary Figure 2. Comparison of effect size (here *Z*) across cross-domain relationships of global and regional models. Plots show the cross-domain relationships between latent intercepts (prefix "i") and latent slopes (prefix "s") across the three domains of interests, i.e. white matter hyperintensities (WMH; total, frontal, posterior), brain morphometrics (here Brain; MTLV-ratio as global, frontal cortical volume, parietal cortical volume as posterior) and cognition (PACC5). Left panel shows intercept-intercept associations, and slope-slope associations. Right panel shows intercept-slope associations. Source data are provided as a Source Data file.

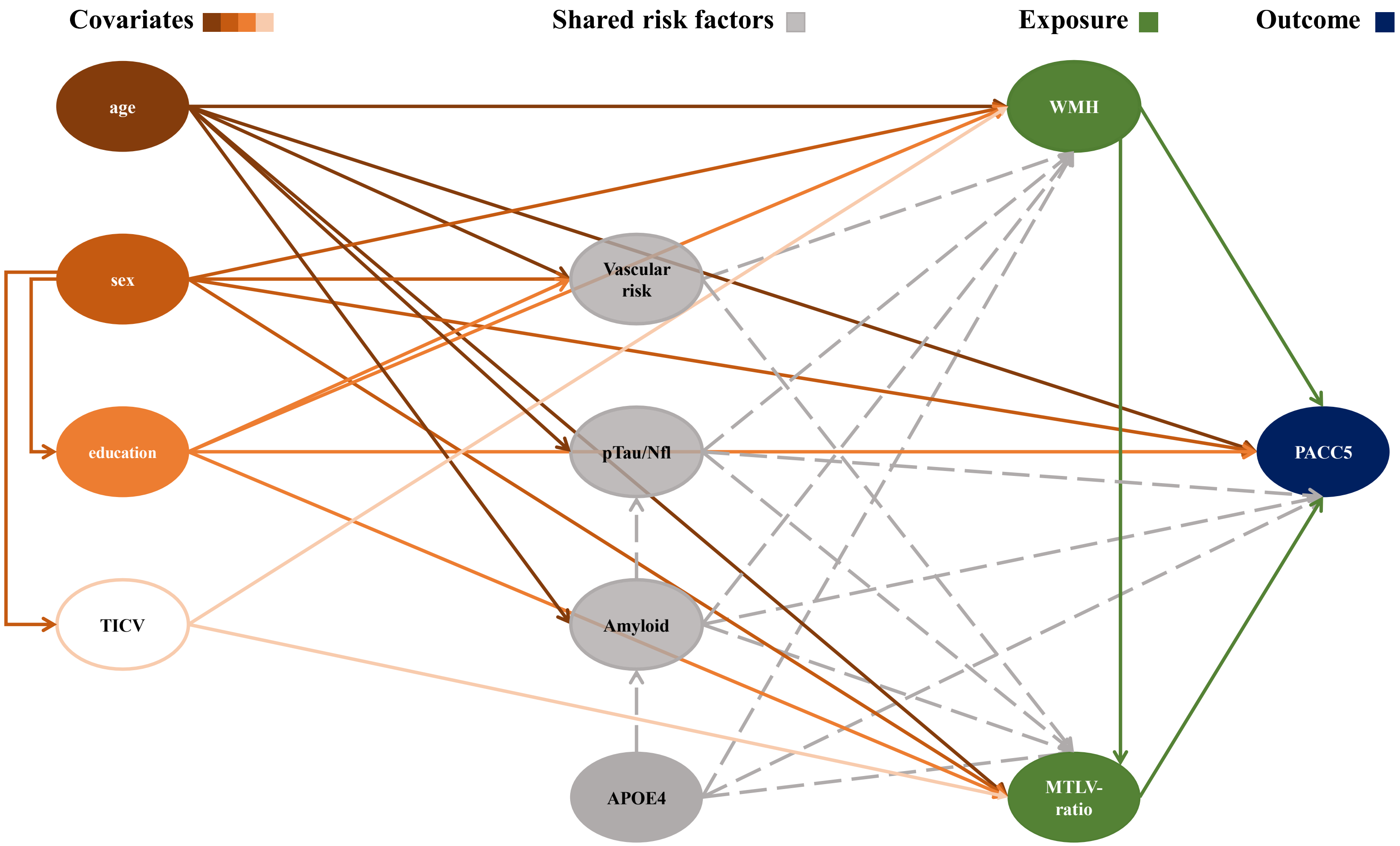

Supplementary Figure 3. Schematic directed acyclic graph (DAG). Covariates were used in the main model. Inclusion of shared risk factors vascular risk, APOE4 and Plasma Amyloid, pTau, and Neurofilament light chain (NfL) was tested in subsequent additional models presented here in supplementary tables 7-10. TICV = total intracranial volume; WMH = white matter hyperintensities; MTLV-ratio = medial temporal lobe to ventricle ratio. PACC5 = preclinical Alzheimer's disease composite score.

Supplementary Table 7. Model details of the main trivariate latent growth curve model between PACC5, total WMH volumes, and MTLV-ratio with additional covariate cardiovascular risk. Model fit: $\chi^2(147) = 166.17$, $p = 0.133$; *CFI* = 0.998; *RMSEA* = 0.019 [0.000, 0.036]; *SRMR* = 0.019; *Yuan-Bentler scaling factor* = 1.081. Adding cardiovascular risk did not significantly improve model fit compared to the main model; One-sided Satorra-Bentler scaled χ^2 difference test: $\Delta\chi^2(12) = 11.11$, p = 0.520. P-values below machine precision are reported as < 2.2×10^{-16}.

				Estimate	SE	95% CI	Std. Estimate	Z	p
Association with Demographics	Intercept cognition	~	Age	-0.315	0.033	-0.38, -0.25	-0.398	-9.645	< 2.2×10^-16
		~	Sex	0.282	0.031	0.22, 0.34	0.356	9.014	< 2.2×10^-16
		~	Years of Education	0.204	0.032	0.14, 0.27	0.257	6.338	2.32×10^-10
	Slope cognition	~	Age	-0.042	0.009	-0.06, -0.02	-0.368	-4.783	1.73×10^-06
		~	Sex	-0.008	0.009	-0.02, 0.01	-0.068	-0.910	0.363
		~	Years of Education	0.014	0.009	-0.00, 0.03	0.120	1.577	0.115
	Intercept WMH	~	Age	0.352	0.038	0.28, 0.43	0.368	9.236	< 2.2×10^-16
		~	Sex	0.174	0.054	0.07, 0.28	0.181	3.228	0.001
		~	Years of Education	-0.053	0.039	-0.13, 0.02	-0.056	-1.364	0.173
		~	TICV	0.246	0.050	0.15, 0.34	0.257	4.889	1.01×10^-06
		~	vascular risk	0.074	0.038	0.00, 0.15	0.078	1.968	0.049
	Slope WMH	~	Age	-0.006	0.004	-0.01, 0.00	-0.102	-1.446	0.148
		~	Sex	0.001	0.006	-0.01, 0.01	0.020	0.215	0.830
		~	Years of Education	-0.003	0.004	-0.01, 0.01	-0.048	-0.719	0.472
		~	TICV	-0.003	0.005	-0.01, 0.01	-0.049	-0.633	0.527
		~	vascular risk	-0.002	0.004	-0.01, 0.01	-0.024	-0.368	0.713
	Intercept brain	~	Age	-0.351	0.028	-0.41, -0.30	-0.493	-12.571	< 2.2×10^-16
		~	Sex	0.098	0.034	0.03, 0.17	0.138	2.868	0.004
		~	Years of Education	0.068	0.028	0.01, 0.12	0.096	2.467	0.014
		~	TICV	-0.136	0.031	-0.20, -0.07	-0.191	-4.359	1.30×10^-05
		~	vascular risk	-0.032	0.026	-0.08, 0.02	-0.045	-1.253	0.210
	Linear slope brain	~	Age	-0.039	0.006	-0.05, -0.03	-0.434	-6.252	4.05×10^-10

				Estimate	SE	95% CI	Std. Estimate	Z	p
		~	Sex	0.001	0.007	-0.01, 0.02	0.008	0.093	0.926
		~	Years of Education	-0.004	0.006	-0.02, 0.01	-0.051	-0.739	0.460
		~	TICV	-0.009	0.008	-0.02, 0.01	-0.106	-1.181	0.238
		~	vascular risk	0.002	0.006	-0.01, 0.01	0.023	0.325	0.745
	Quadratic slope brain	~	Age	0.002	0.001	-0.00, 0.00	0.147	1.259	0.208
		~	Sex	0.002	0.002	-0.00, 0.00	0.132	0.921	0.357
		~	Years of Education	0.003	0.001	0.00, 0.01	0.264	2.236	0.025
		~	TICV	-0.001	0.002	-0.00, 0.00	-0.089	-0.697	0.486
		~	vascular risk	-0.001	0.001	-0.00, 0.00	-0.052	-0.457	0.648
**Association between latent variables**	Intercept cognition	~~	Slope cognition	0.015	0.007	0.00, 0.03	0.226	2.105	0.035
		~~	Intercept WMH	-0.030	0.023	-0.08, 0.02	-0.057	-1.301	0.193
		~~	Slope WMH	0.002	0.003	-0.00, 0.01	0.043	0.551	0.582
		~~	Intercept brain	0.051	0.018	0.02, 0.09	0.148	2.819	0.005
		~~	Linear slope brain	0.008	0.004	-0.00, 0.02	0.169	1.893	0.058
		~~	Quadratic slope brain	0.000	0.001	-0.00, 0.00	-0.060	-0.438	0.661
	Slope cognition	~~	Intercept WMH	-0.004	0.007	-0.02, 0.01	-0.046	-0.565	0.572
		~~	Slope WMH	-0.001	0.001	-0.00, -0.00	-0.210	-2.031	0.042
		~~	Intercept brain	0.015	0.005	0.00, 0.03	0.254	2.790	0.005
		~~	Linear slope brain	0.003	0.001	0.00, 0.01	0.356	2.457	0.014
		~~	Quadratic slope brain	0.000	0.000	-0.00, 0.00	0.219	1.044	0.296
	Intercept WMH	~~	Slope WMH	-0.003	0.004	-0.01, 0.00	-0.064	-0.819	0.413
		~~	Intercept brain	-0.062	0.020	-0.10, -0.02	-0.131	-3.074	0.002
		~~	Linear slope brain	-0.013	0.005	-0.02, -0.00	-0.188	-2.731	0.006
		~~	Quadratic slope brain	0.000	0.001	-0.00, 0.00	0.034	0.302	0.763
	Slope WMH	~~	Intercept brain	-0.002	0.003	-0.01, 0.00	-0.047	-0.629	0.529
		~~	Linear slope brain	-0.001	0.000	-0.00, 0.00	-0.131	-1.459	0.145

				Estimate	SE	95% CI	Std. Estimate	Z	p
		~~	Quadratic slope brain	0.000	0.000	-0.00, 0.00	-0.086	-0.637	0.524
	Intercept brain	~~	Linear slope brain	0.018	0.004	0.01, 0.03	0.414	4.370	1.24×10^-05
		~~	Quadratic slope brain	0.002	0.001	-0.00, 0.00	0.242	1.584	0.113
	Linear slope brain	~~	Quadratic slope brain	0.000	0.000	-0.00, 0.00	-0.355	-1.064	0.287
**Association covariates**	Age	~~	Sex	-0.194	0.042	-0.28, -0.11	-0.194	-4.657	3.21×10^-06
		~~	TICV	0.103	0.041	0.02, 0.18	0.103	2.497	0.013
		~~	Years of Education	-0.111	0.046	-0.20, -0.02	-0.111	-2.432	0.015
		~~	vascular risk	0.098	0.044	0.01, 0.19	0.098	2.212	0.027
	Sex	~~	TICV	-0.665	0.020	-0.70, -0.63	-0.665	-33.182	< 2.2×10^-16
		~~	Years of Education	-0.227	0.039	-0.30, -0.15	-0.227	-5.793	6.91×10^-09
		~~	vascular risk	-0.190	0.040	-0.27, -0.11	-0.190	-4.695	2.66×10^-06
	Years of Education	~~	TICV	0.251	0.038	0.18, 0.33	0.251	6.521	6.97×10^-11
	TICV	~~	vascular risk	0.123	0.045	0.04, 0.21	0.123	2.749	0.006
	Years of Education	~~	vascular risk	-0.112	0.041	-0.19, -0.03	-0.112	-2.736	0.006

Supplementary Table 8. Model details of the trivariate latent growth curve model with additional covariates APOE-ε4, and Plasma Aβ42/40. Model fit: $\chi^2(155) = 165.32$, $p = 0.271$; $CFI = 0.999$; $RMSEA = 0.015$ [0.000, 0.033]; $SRMR = 0.017$; *Yuan-Bentler scaling factor* = 1.076. Adding APOE-ε4 and Plasma Aβ42/40 did not significantly improve model fit compared to the main model; One-sided Satorra-Bentler scaled χ^2 difference test: $\Delta\chi^2(20) = 9.87$, p = 0.971. P-values below machine precision are reported as $< 2.2\times10^{-16}$.

				Estimate	SE	95% CI	Std. Estimate	Z	p
Association with Demographics	Intercept cognition	~	Age	-0.309	0.033	-0.37, -0.25	-0.391	-9.420	< 2.2×10^-16
		~	Sex	0.275	0.033	0.21, 0.34	0.348	8.304	< 2.2×10^-16
		~	Years of Education	0.203	0.032	0.14, 0.27	0.257	6.328	2.49×10^-10
		~	Plasma Amyloid	-0.027	0.038	-0.10, 0.05	-0.034	-0.713	0.476
		~	APOE4	0.010	0.032	-0.05, 0.07	0.012	0.301	0.763
	Slope cognition	~	Age	-0.040	0.009	-0.06, -0.02	-0.351	-4.510	6.47×10^-06
		~	Sex	-0.014	0.009	-0.03, 0.00	-0.123	-1.548	0.122
		~	Years of Education	0.014	0.009	-0.00, 0.03	0.121	1.604	0.109
		~	Plasma Amyloid	-0.017	0.012	-0.04, 0.01	-0.146	-1.369	0.171
		~	APOE4	-0.016	0.009	-0.03, 0.00	-0.139	-1.760	0.078
	Intercept WMH	~	Age	0.342	0.039	0.27, 0.42	0.357	8.731	< 2.2×10^-16
		~	Sex	0.176	0.055	0.07, 0.28	0.184	3.185	0.001
		~	Years of Education	-0.065	0.039	-0.14, 0.01	-0.067	-1.655	0.098
		~	TICV	0.243	0.051	0.14, 0.34	0.254	4.790	1.66×10^-06
		~	Plasma Amyloid	0.071	0.049	-0.03, 0.17	0.074	1.445	0.148
		~	APOE4	0.015	0.041	-0.07, 0.10	0.016	0.365	0.715
	Slope WMH	~	Age	-0.006	0.004	-0.01, 0.00	-0.096	-1.339	0.180
		~	Sex	0.001	0.006	-0.01, 0.01	0.020	0.213	0.831
		~	Years of Education	-0.003	0.004	-0.01, 0.01	-0.043	-0.646	0.518
		~	TICV	-0.003	0.005	-0.01, 0.01	-0.052	-0.651	0.515
		~	Plasma Amyloid	-0.002	0.005	-0.01, 0.01	-0.028	-0.359	0.720
		~	APOE4	0.003	0.005	-0.01, 0.01	0.045	0.606	0.545

				Estimate	SE	95% CI	Std. Estimate	Z	p
	Intercept brain	~	Age	-0.356	0.028	-0.41, -0.30	-0.499	-12.657	< 2.2×10^-16
		~	Sex	0.108	0.035	0.04, 0.18	0.152	3.143	0.002
		~	Years of Education	0.073	0.027	0.02, 0.13	0.103	2.713	0.007
		~	TICV	-0.136	0.032	-0.20, -0.07	-0.191	-4.290	1.79×10^-05
		~	Plasma Amyloid	0.021	0.032	-0.04, 0.08	0.029	0.644	0.520
		~	APOE4	-0.035	0.027	-0.09, 0.02	-0.049	-1.316	0.188
	Linear slope brain	~	Age	-0.037	0.006	-0.05, -0.03	-0.423	-6.018	1.77×10^-09
		~	Sex	-0.001	0.008	-0.02, 0.01	-0.012	-0.142	0.887
		~	Years of Education	-0.005	0.006	-0.02, 0.01	-0.057	-0.859	0.390
		~	TICV	-0.007	0.008	-0.02, 0.01	-0.077	-0.871	0.384
		~	Plasma Amyloid	-0.006	0.007	-0.02, 0.01	-0.073	-0.963	0.335
		~	APOE4	-0.018	0.006	-0.03, -0.01	-0.208	-2.996	0.003
	Quadratic slope brain	~	Age	0.002	0.001	-0.00, 0.00	0.132	1.104	0.269
		~	Sex	0.002	0.002	-0.00, 0.01	0.141	0.955	0.340
		~	Years of Education	0.003	0.001	0.00, 0.01	0.270	2.227	0.026
		~	TICV	-0.001	0.002	-0.00, 0.00	-0.110	-0.850	0.395
		~	Plasma Amyloid	0.001	0.001	-0.00, 0.00	0.056	0.453	0.651
		~	APOE4	0.000	0.002	-0.00, 0.00	0.011	0.083	0.934
**Association between latent variables**	Intercept cognition	~~	Slope cognition	0.014	0.007	0.00, 0.03	0.228	2.085	0.037
		~~	Intercept WMH	-0.032	0.023	-0.08, 0.01	-0.059	-1.354	0.176
		~~	Slope WMH	0.002	0.003	-0.00, 0.01	0.044	0.572	0.567
		~~	Intercept brain	0.052	0.018	0.02, 0.09	0.151	2.893	0.004
		~~	Linear slope brain	0.008	0.004	-0.00, 0.02	0.168	1.860	0.063
		~~	Quadratic slope brain	0.000	0.001	-0.00, 0.00	-0.058	-0.424	0.672
	Slope cognition	~~	Intercept WMH	-0.003	0.007	-0.02, 0.01	-0.038	-0.454	0.650
		~~	Slope WMH	-0.001	0.001	-0.00, -0.00	-0.212	-2.034	0.042

				Estimate	SE	95% CI	Std. Estimate	Z	p
		~~	Intercept brain	0.015	0.005	0.00, 0.03	0.269	2.894	0.004
		~~	Linear slope brain	0.002	0.001	0.00, 0.00	0.317	2.109	0.035
		~~	Quadratic slope brain	0.000	0.000	-0.00, 0.00	0.258	1.206	0.228
	Intercept WMH	~~	Slope WMH	-0.003	0.004	-0.01, 0.00	-0.064	-0.835	0.404
		~~	Intercept brain	-0.066	0.020	-0.11, -0.03	-0.137	-3.217	0.001
		~~	Linear slope brain	-0.012	0.005	-0.02, -0.00	-0.180	-2.528	0.011
		~~	Quadratic slope brain	0.000	0.001	-0.00, 0.00	0.020	0.180	0.857
	Slope WMH	~~	Intercept brain	-0.001	0.002	-0.01, 0.00	-0.041	-0.575	0.565
		~~	Linear slope brain	-0.001	0.000	-0.00, 0.00	-0.126	-1.303	0.193
		~~	Quadratic slope brain	0.000	0.000	-0.00, 0.00	-0.085	-0.623	0.533
	Intercept brain	~~	Linear slope brain	0.018	0.004	0.01, 0.03	0.419	4.327	1.51×10^{-05}
		~~	Quadratic slope brain	0.002	0.001	-0.00, 0.00	0.246	1.596	0.110
	Linear slope brain	~~	Quadratic slope brain	0.000	0.000	-0.00, 0.00	-0.351	-1.041	0.298
**Association covariates**	Age	~~	Sex	-0.194	0.042	-0.28, -0.11	-0.194	-4.660	3.16×10^{-06}
		~~	TICV	0.103	0.041	0.02, 0.18	0.103	2.499	0.012
		~~	Years of Education	-0.111	0.046	-0.20, -0.02	-0.111	-2.428	0.015
		~~	Plasma Amyloid	0.242	0.049	0.15, 0.34	0.242	4.917	8.80×10^{-07}
		~~	APOE4	0.009	0.043	-0.08, 0.09	0.009	0.205	0.838
	Sex	~~	TICV	-0.665	0.020	-0.70, -0.63	-0.665	-33.200	$< 2.2\times10^{-16}$
		~~	Years of Education	-0.227	0.039	-0.30, -0.15	-0.227	-5.784	7.28×10^{-09}
		~~	Plasma Amyloid	-0.315	0.040	-0.39, -0.24	-0.315	-7.885	3.11×10^{-15}
		~~	APOE4	-0.071	0.043	-0.16, 0.01	-0.071	-1.635	0.102
	Years of Education	~~	TICV	0.251	0.039	0.18, 0.33	0.251	6.509	7.58×10^{-11}
	Plasma Amyloid	~~	TICV	0.230	0.041	0.15, 0.31	0.230	5.621	1.89×10^{-08}
	APOE4	~~	TICV	0.109	0.043	0.02, 0.19	0.109	2.527	0.012
	Years of Education	~~	Plasma Amyloid	0.037	0.048	-0.06, 0.13	0.037	0.774	0.439

			Estimate	SE	95% CI	Std. Estimate	Z	p
	~~	APOE4	0.023	0.043	-0.06, 0.11	0.023	0.537	0.591
Plasma Amyloid	~~	APOE4	0.247	0.045	0.16, 0.33	0.247	5.496	3.88×10^-08

Supplementary Table 9. Model details of the main trivariate latent growth curve model between PACC5, total WMH volumes, and MTLV-ratio with additional covariate plasma pTau181. Model fit: $\chi^2(145) = 148.15$, $p = 0.412$; $CFI = 0.999$; $RMSEA = 0.012$ [0.000, 0.033]; $SRMR = 0.017$; *Yuan-Bentler scaling factor* = 1.151. Adding plasma pTau181 did not significantly improve model fit compared to the main model; One-sided Satorra-Bentler scaled χ^2 difference test: $\Delta\chi^2(10) = 2.52$, $p = 0.990$. P-values below machine precision are reported as $< 2.2\times10^{-16}$.

				Estimate	SE	95% CI	Std. Estimate	Z	p
Association with Demographics	Intercept cognition	~	Age	-0.297	0.033	-0.36, -0.23	-0.376	-9.082	< 2.2×10^-16
		~	Sex	0.276	0.031	0.22, 0.34	0.349	8.919	< 2.2×10^-16
		~	Years of Education	0.208	0.032	0.15, 0.27	0.263	6.557	5.51×10^-11
		~	Plasma pTau181	-0.073	0.028	-0.13, -0.02	-0.092	-2.603	0.009
	Slope cognition	~	Age	-0.036	0.009	-0.05, -0.02	-0.316	-3.981	6.87×10^-05
		~	Sex	-0.010	0.009	-0.03, 0.01	-0.085	-1.132	0.257
		~	Years of Education	0.014	0.009	-0.00, 0.03	0.125	1.670	0.095
		~	Plasma pTau181	-0.028	0.009	-0.05, -0.01	-0.249	-3.239	0.001
	Intercept WMH	~	Age	0.336	0.041	0.26, 0.42	0.351	8.233	2.22×10^-16
		~	Sex	0.169	0.054	0.06, 0.27	0.177	3.156	0.002
		~	Years of Education	-0.070	0.039	-0.15, 0.01	-0.073	-1.800	0.072
		~	TICV	0.256	0.051	0.16, 0.36	0.267	5.008	5.51×10^-07
		~	Plasma pTau181	0.080	0.043	-0.00, 0.16	0.084	1.883	0.060
	Slope WMH	~	Age	-0.006	0.004	-0.01, 0.00	-0.095	-1.346	0.178
		~	Sex	0.001	0.006	-0.01, 0.01	0.019	0.196	0.844
		~	Years of Education	-0.003	0.004	-0.01, 0.01	-0.043	-0.654	0.513
		~	TICV	-0.003	0.005	-0.01, 0.01	-0.056	-0.705	0.481
		~	Plasma pTau181	-0.003	0.003	-0.01, 0.00	-0.046	-0.825	0.409
	Intercept brain	~	Age	-0.341	0.029	-0.40, -0.28	-0.478	-11.915	< 2.2×10^-16
		~	Sex	0.099	0.034	0.03, 0.16	0.138	2.920	0.004
		~	Years of Education	0.076	0.027	0.02, 0.13	0.107	2.840	0.005
		~	TICV	-0.141	0.032	-0.20, -0.08	-0.197	-4.453	8.45×10^-06

				Estimate	SE	95% CI	Std. Estimate	Z	p
		~	Plasma pTau181	-0.052	0.024	-0.10, -0.01	-0.073	-2.228	0.026
	Linear slope brain	~	Age	-0.035	0.006	-0.05, -0.02	-0.394	-5.523	3.34×10^-08
		~	Sex	-0.001	0.007	-0.02, 0.01	-0.014	-0.168	0.866
		~	Years of Education	-0.004	0.006	-0.02, 0.01	-0.043	-0.650	0.516
		~	TICV	-0.010	0.008	-0.03, 0.01	-0.118	-1.310	0.190
		~	Plasma pTau181	-0.015	0.006	-0.03, -0.00	-0.173	-2.674	0.007
	Quadratic slope brain	~	Age	0.002	0.001	-0.00, 0.00	0.147	1.231	0.218
		~	Sex	0.002	0.002	-0.00, 0.01	0.134	0.915	0.360
		~	Years of Education	0.003	0.001	0.00, 0.01	0.264	2.226	0.026
		~	TICV	-0.001	0.002	-0.00, 0.00	-0.097	-0.748	0.455
		~	Plasma pTau181	-0.001	0.001	-0.00, 0.00	-0.046	-0.407	0.684
**Association between latent variables**	Intercept cognition	~~	Slope cognition	0.013	0.007	-0.00, 0.03	0.206	1.863	0.063
		~~	Intercept WMH	-0.028	0.023	-0.07, 0.02	-0.053	-1.215	0.224
		~~	Slope WMH	0.002	0.003	-0.00, 0.01	0.040	0.519	0.604
		~~	Intercept brain	0.049	0.018	0.01, 0.08	0.143	2.733	0.006
		~~	Linear slope brain	0.007	0.004	-0.00, 0.02	0.152	1.701	0.089
		~~	Quadratic slope brain	0.000	0.001	-0.00, 0.00	-0.061	-0.457	0.648
	Slope cognition	~~	Intercept WMH	-0.003	0.007	-0.02, 0.01	-0.034	-0.406	0.685
		~~	Slope WMH	-0.001	0.001	-0.00, -0.00	-0.225	-2.101	0.036
		~~	Intercept brain	0.014	0.005	0.00, 0.02	0.251	2.695	0.007
		~~	Linear slope brain	0.003	0.001	0.00, 0.00	0.323	2.146	0.032
		~~	Quadratic slope brain	0.000	0.000	-0.00, 0.00	0.226	1.081	0.280
	Intercept WMH	~~	Slope WMH	-0.003	0.004	-0.01, 0.00	-0.062	-0.798	0.425
		~~	Intercept brain	-0.062	0.020	-0.10, -0.02	-0.130	-3.034	0.002
		~~	Linear slope brain	-0.012	0.005	-0.02, -0.00	-0.175	-2.555	0.011
		~~	Quadratic slope brain	0.000	0.001	-0.00, 0.00	0.030	0.270	0.787

				Estimate	SE	95% CI	Std. Estimate	Z	p
	Slope WMH	~~	Intercept brain	-0.002	0.002	-0.01, 0.00	-0.048	-0.647	0.517
		~~	Linear slope brain	-0.001	0.000	-0.00, 0.00	-0.138	-1.523	0.128
		~~	Quadratic slope brain	0.000	0.000	-0.00, 0.00	-0.091	-0.677	0.498
	Intercept brain	~~	Linear slope brain	0.017	0.004	0.01, 0.03	0.405	4.212	2.53×10^-05
		~~	Quadratic slope brain	0.002	0.001	-0.00, 0.00	0.250	1.645	0.100
	Linear slope brain	~~	Quadratic slope brain	0.000	0.000	-0.00, 0.00	-0.361	-1.067	0.286
**Association covariates**	Age	~~	Sex	-0.194	0.041	-0.28, -0.11	-0.194	-4.680	2.87×10^-06
		~~	TICV	0.103	0.041	0.02, 0.18	0.103	2.495	0.013
		~~	Years of Education	-0.111	0.046	-0.20, -0.02	-0.111	-2.424	0.015
		~~	Plasma pTau181	0.249	0.100	0.05, 0.44	0.249	2.488	0.013
	Sex	~~	TICV	-0.665	0.020	-0.70, -0.63	-0.665	-33.091	< 2.2×10^-16
		~~	Years of Education	-0.227	0.039	-0.30, -0.15	-0.227	-5.787	7.17×10^-09
		~~	Plasma pTau181	-0.133	0.069	-0.27, 0.00	-0.133	-1.927	0.054
	Years of Education	~~	TICV	0.251	0.039	0.18, 0.33	0.251	6.513	7.36×10^-11
	Plasma pTau181	~~	TICV	0.026	0.070	-0.11, 0.16	0.026	0.372	0.710
	Years of Education	~~	Plasma pTau181	0.044	0.046	-0.05, 0.13	0.044	0.958	0.338

Supplementary Table 10. Model details of the main trivariate latent growth curve model between PACC5, total WMH volumes, and MTLV-ratio with additional covariate plasma neurofilament light chain (NfL). Model fit: $\chi^2(145) = 159.27$, $p = 0.197$; *CFI* = 0.997; *RMSEA* = 0.028 [0.000, 0.042]; *SRMR* = 0.017; *Yuan-Bentler scaling factor* = 1.152. Adding plasma NfL did not significantly improve model fit compared to the main model; One-sided Satorra-Bentler scaled χ^2 difference test: $\Delta\chi^2(10) = 7.47$, $p = 0.680$. P-values below machine precision are reported as $< 2.2\times10^{-16}$.

				Estimate	SE	95% CI	Std. Estimate	Z	p
Association with Demographics	Intercept cognition	~	Age	-0.309	0.036	-0.38, -0.24	-0.391	-8.675	< 2.2×10^-16
		~	Sex	0.282	0.031	0.22, 0.34	0.356	8.996	< 2.2×10^-16
		~	Years of Education	0.205	0.032	0.14, 0.27	0.259	6.395	1.61×10^-10
		~	Plasma NfL	-0.013	0.037	-0.09, 0.06	-0.017	-0.357	0.721
	Slope cognition	~	Age	-0.031	0.010	-0.05, -0.01	-0.267	-3.143	0.002
		~	Sex	-0.008	0.008	-0.02, 0.01	-0.066	-0.897	0.370
		~	Years of Education	0.012	0.009	-0.00, 0.03	0.108	1.446	0.148
		~	Plasma NfL	-0.031	0.010	-0.05, -0.01	-0.269	-3.055	0.002
	Intercept WMH	~	Age	0.324	0.042	0.24, 0.41	0.339	7.746	9.55×10^-15
		~	Sex	0.154	0.053	0.05, 0.26	0.161	2.882	0.004
		~	Years of Education	-0.065	0.039	-0.14, 0.01	-0.068	-1.670	0.095
		~	TICV	0.242	0.051	0.14, 0.34	0.253	4.739	2.15×10^-06
		~	Plasma NfL	0.077	0.034	0.01, 0.14	0.080	2.266	0.023
	Slope WMH	~	Age	-0.005	0.005	-0.01, 0.00	-0.089	-1.193	0.233
		~	Sex	0.002	0.006	-0.01, 0.01	0.028	0.294	0.769
		~	Years of Education	-0.003	0.004	-0.01, 0.01	-0.046	-0.683	0.495
		~	TICV	-0.003	0.005	-0.01, 0.01	-0.046	-0.597	0.550
		~	Plasma NfL	-0.002	0.004	-0.01, 0.01	-0.037	-0.522	0.602
	Intercept brain	~	Age	-0.320	0.030	-0.38, -0.26	-0.447	-10.650	< 2.2×10^-16
		~	Sex	0.110	0.034	0.04, 0.18	0.154	3.283	0.001
		~	Years of Education	0.071	0.027	0.02, 0.12	0.100	2.643	0.008

				Estimate	SE	95% CI	Std. Estimate	Z	p
		~	TICV	-0.131	0.032	-0.19, -0.07	-0.183	-4.137	3.52×10^-05
		~	Plasma NfL	-0.088	0.029	-0.15, -0.03	-0.123	-3.009	0.003
	Linear slope brain	~	Age	-0.033	0.007	-0.05, -0.02	-0.374	-5.027	4.99×10^-07
		~	Sex	0.001	0.007	-0.01, 0.02	0.014	0.174	0.862
		~	Years of Education	-0.005	0.006	-0.02, 0.01	-0.059	-0.895	0.371
		~	TICV	-0.008	0.008	-0.02, 0.01	-0.093	-1.060	0.289
		~	Plasma NfL	-0.014	0.009	-0.03, 0.00	-0.159	-1.619	0.105
	Quadratic slope brain	~	Age	0.002	0.002	-0.00, 0.01	0.181	1.451	0.147
		~	Sex	0.002	0.002	-0.00, 0.01	0.142	0.998	0.318
		~	Years of Education	0.003	0.001	0.00, 0.01	0.267	2.263	0.024
		~	TICV	-0.001	0.002	-0.00, 0.00	-0.085	-0.685	0.494
		~	Plasma NfL	-0.001	0.002	-0.01, 0.00	-0.099	-0.574	0.566
**Association between latent variables**	Intercept cognition	~~	Slope cognition	0.015	0.007	0.00, 0.03	0.235	2.130	0.033
		~~	Intercept WMH	-0.032	0.023	-0.08, 0.01	-0.061	-1.387	0.165
		~~	Slope WMH	0.002	0.003	-0.00, 0.01	0.045	0.574	0.566
		~~	Intercept brain	0.052	0.018	0.02, 0.09	0.153	2.897	0.004
		~~	Linear slope brain	0.008	0.004	-0.00, 0.02	0.170	1.908	0.056
		~~	Quadratic slope brain	0.000	0.001	-0.00, 0.00	-0.047	-0.349	0.727
	Slope cognition	~~	Intercept WMH	-0.003	0.007	-0.02, 0.01	-0.032	-0.379	0.705
		~~	Slope WMH	-0.001	0.001	-0.00, -0.00	-0.229	-2.134	0.033
		~~	Intercept brain	0.013	0.005	0.00, 0.02	0.240	2.627	0.009
		~~	Linear slope brain	0.003	0.001	0.00, 0.00	0.334	2.232	0.026
		~~	Quadratic slope brain	0.000	0.000	-0.00, 0.00	0.206	1.001	0.317
	Intercept WMH	~~	Slope WMH	-0.003	0.004	-0.01, 0.00	-0.060	-0.783	0.434
		~~	Intercept brain	-0.061	0.020	-0.10, -0.02	-0.129	-3.025	0.002
		~~	Linear slope brain	-0.012	0.005	-0.02, -0.00	-0.179	-2.662	0.008

				Estimate	SE	95% CI	Std. Estimate	Z	p
		~~	Quadratic slope brain	0.000	0.001	-0.00, 0.00	0.037	0.336	0.737
	Slope WMH	~~	Intercept brain	-0.002	0.002	-0.01, 0.00	-0.050	-0.683	0.495
		~~	Linear slope brain	-0.001	0.000	-0.00, 0.00	-0.144	-1.594	0.111
		~~	Quadratic slope brain	0.000	0.000	-0.00, 0.00	-0.082	-0.610	0.542
	Intercept brain	~~	Linear slope brain	0.017	0.004	0.01, 0.03	0.405	4.243	2.21×10^-05
		~~	Quadratic slope brain	0.002	0.001	-0.00, 0.00	0.240	1.589	0.112
	Linear slope brain	~~	Quadratic slope brain	0.000	0.000	-0.00, 0.00	-0.369	-1.097	0.273
**Association covariates**	Age	~~	Sex	-0.194	0.042	-0.28, -0.11	-0.194	-4.660	3.16×10^-06
		~~	TICV	0.103	0.041	0.02, 0.18	0.103	2.512	0.012
		~~	Years of Education	-0.111	0.046	-0.20, -0.02	-0.111	-2.432	0.015
		~~	Plasma NfL	0.405	0.050	0.31, 0.50	0.405	8.171	2.22×10^-16
	Sex	~~	TICV	-0.665	0.020	-0.70, -0.63	-0.665	-33.187	< 2.2×10^-16
		~~	Years of Education	-0.227	0.039	-0.30, -0.15	-0.227	-5.791	6.99×10^-09
		~~	Plasma NfL	-0.075	0.039	-0.15, 0.00	-0.075	-1.908	0.056
	Years of Education	~~	TICV	0.251	0.039	0.18, 0.33	0.251	6.507	7.65×10^-11
	Plasma NfL	~~	TICV	0.088	0.038	0.01, 0.16	0.088	2.299	0.021
	Years of Education	~~	Plasma NfL	-0.040	0.061	-0.16, 0.08	-0.040	-0.666	0.506

Assessment of Plasma levels of Aβ42/40, its correlation with CSF-derived Aβ42/40 ratio, as well as its diagnostic and predictive utility in the DELCODE cohort are outlined in[10], and determination of plasma pTau181 and Nfl are outlined in[11]. Plasma levels of Aβ42/40 were available for 417 individuals, plasma pTau levels for 469 individuals, and NfL levels for 512 individuals. We opted for plasma instead of CSF-derived markers, as the latter was only available in 185 individuals. Moreover, plasma Aβ42/40 has been shown to associate and discriminate well with regards to Aβ-PET[12,13], especially when combined with APOE-ε4 status[10,14,15]. We reversed plasma Aβ42/40 levels to facilitate interpretation of effects, i.e., higher levels denote more pathological levels. We determined APOE-ε4 carriership based on the presence of at least one ε4 allele ($n_{carrier} = 150$; $n_{non\text{-}carrier} = 388$; $n_{missing} = 5$). The procedure for APOE genotyping is described in detail in[16].

Higher cardiovascular risk appeared to relate to higher baseline levels of total WMH (Supplementary Table 7). Individuals with APOE-ε4 carriership had steeper declines in MTLV-ratios[17–19] (Supplementary Table 8). APOE-ε4 carriership was not associated with baseline levels of PACC5 performance, total WMH, or MTLV ratio, nor with changes in PACC5 performance or WMH over time (Supplementary Table 8). There were no significant associations of more pathological baseline levels of plasma Aβ42/40 with baseline levels or changes in PACC5 performance, WMH, or MTLV-ratios (Supplementary Table 8). Plasma Aβ42/40 is a valid but approximate measure CSF-Aβ42/40 levels[10] and hence might underrepresent AD pathological changes[20].

Higher levels of plasma pTau181 were associated with lower PACC5 baseline performance and faster cognitive decline (Supplementary Table 9). Moreover, they related to smaller baseline MTLV-ratio as well as MTLV-ratio decline (Supplementary Table 9), as previously shown[11].

Higher levels of NfL were related to faster PACC5 performance decline (Supplementary Table 10). Furthermore, higher NfL was linked to higher baseline volumes of total WMH (Supplementary Table 10), and smaller MTLV-ratio at baseline (Supplementary Table 10). Relations between higher NfL levels and cognitive decline as well as WMH burden have previously been described in community dwelling, healthy ageing individuals[21–23]. Both blood-based or CSF-based NfL have previously been shown to predict atrophic changes and ventricular enlargement[24,25]. However, these associations remain mixed in healthy ageing individuals[23,24,26], which might be explained by its high variability after the sixth life decade[22].

Overall, adjusting for these biomarkers, did not, result in changes to the qualitative interpretation of associations between latent intercepts and latent slopes between WMH, MTLV-ratio, and PACC5 performance[27–32].

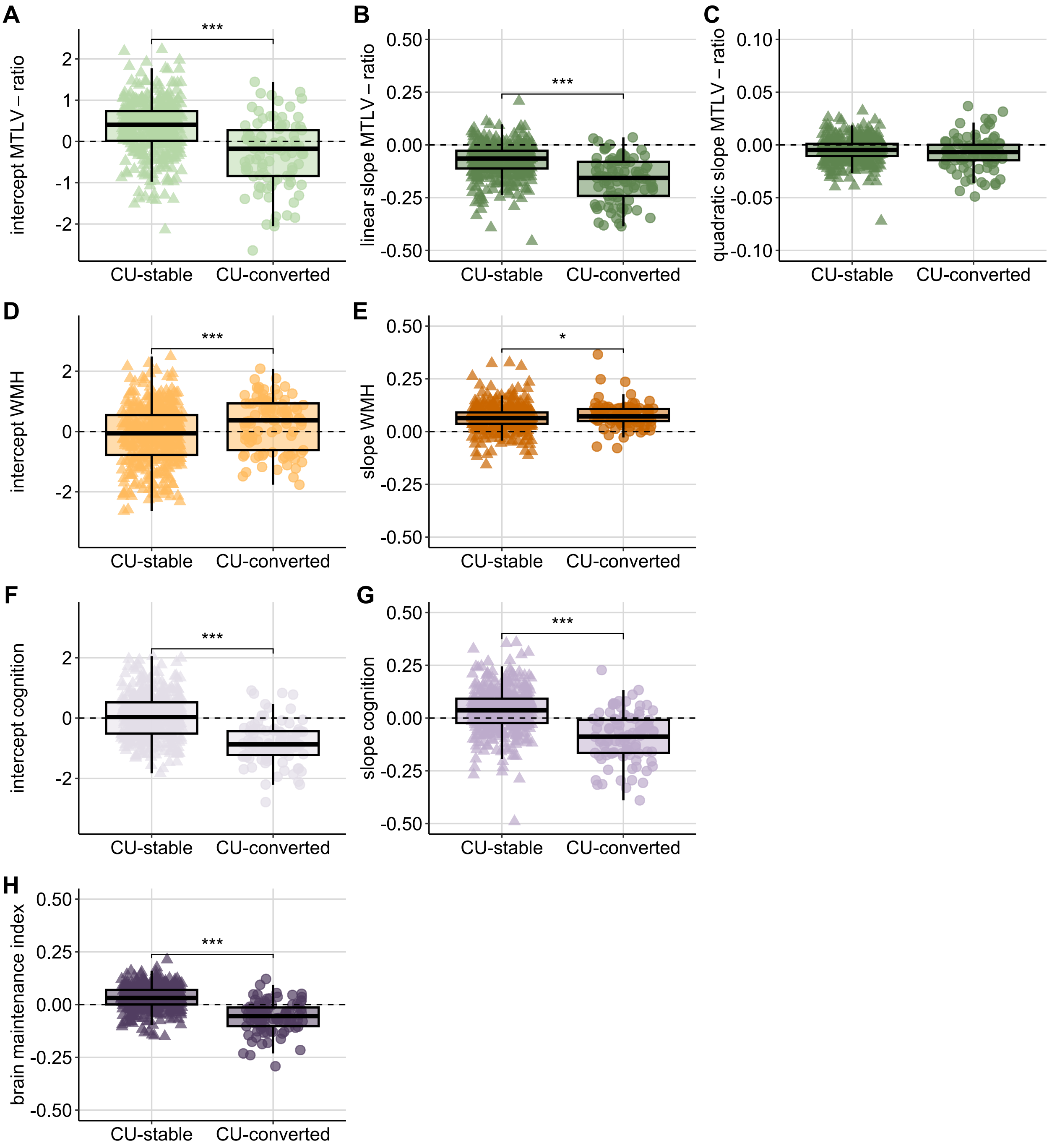

Supplementary Figure 4. Comparison of factor scores between cognitively stable and converting individuals reveal unfavourable pathological levels and progression within neurocognitive domains and brain maintenance index in individuals who converted to MCI or dementia during the course of the study. Factor scores derived from the trivariate LGCM accounted for effects of age, sex, years of education, and in the case of WMH and MTLV-ratio for total intracranial volume (TICV). The brain maintenance index was defined by predicted values of cognitive slope retrieved from robust multiple regression model, predicting cognitive slope by

brain-domain changes and baseline levels of all neurocognitive domains. **(A-C)** MTLV-ratio differed between individuals who progressed to MCI or dementia and cognitively stable individuals regarding baseline levels (U=28430, $p=4.43\times10^{-13}$, r=0.322 [0.23–0.40]) and linear rates of changes (U=29253, $p=3.08\times10^{-15}$, r=0.351 [0.27–0.43])[33–40]. Linear MTLV-ratio decline was present in 95.70% of individuals converting to MCI or AD vs. in 87.65%. of cognitively stable individuals (Fisher's Exact Test: OR=3.13, p=0.026). Quadratic MTLV-ratio change did not significantly differ between cognitively stable and converting individuals (U=21325, p=0.096, r=0.074 [0.01–0.17]). **(D-E)** Total WMH differed between individuals who progressed to MCI or dementia and cognitively stable individuals regarding baseline levels (U=14840, $p=6.13\times10^{-4}$, r=0.152 [0.06–0.24][41,42]) and rates of changes (U=16594, p=0.041, r=0.091 [0.01–0.17][43–46]). WMH progression was present in 94.62% of individuals converting to MCI or dementia vs. in 89.59% of cognitively stable individuals (Fisher's Exact Test: nonsignificant). **(F-G)** Cognition as assessed with the PACC5 differed between individuals who progressed to MCI or dementia and cognitively stable individuals regarding baseline levels (U=31389, $p=1.13\times10^{-21}$, r=0.425 [0.35–0.49]) and rates of changes (U=30674, $p=2.19\times10^{-19}$, r=0.400 [0.33–0.46])[47–51]. PACC5 performance decline was present in 78.49% of individuals converting to MCI or dementia vs. in 33.66% of cognitively stable individuals (Fisher's Exact Test: OR=7.16, $p=2.86\times10^{-15}$). **(H)** Brain maintenance was higher in cognitively stable as compared to converting individuals (U=32378, $p=4.61\times10^{-25}$, r=0.460 [0.39–0.52]). ***p<0.001, **p< 0.01, *p<0.05. Source data are provided as a Source Data file.

3d_scatter_regressio
n.html

Supplementary Figure 5. Interactive 3D Scatterplot of independent effects of WMH and MTLV-ratio on cognitive changes in ageing (*click the icon to access and download the interactive figure*). Colours reflect the slope in cognition with red colours pointing to negative and blue colours to positive slope estimates. Factor scores were extracted from the trivariate LGCM via regression-based method adjusted for effects of age, sex, years of education, and in the case of WMH and MTLV-ratio for total intracranial volume (TICV). Source data are provided as a Source Data file.

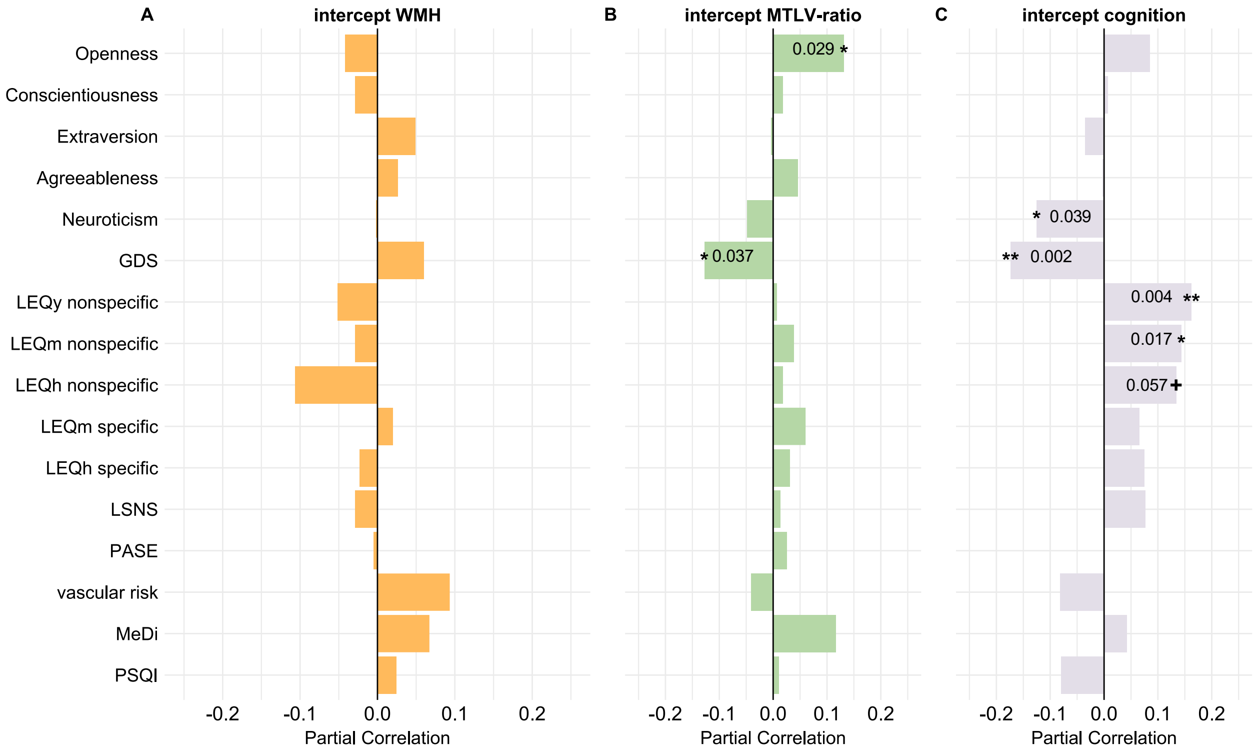

Supplementary Figure 6. Associations of latent intercepts with modifiable lifestyle factors. Factor scores for latent intercepts were derived from the trivariate LGCM via regression-based method. We used partial Spearman's correlations to account for the effects of age, sex, years of education, and TICV. All correlations were FDR-corrected. Panels show relations between lifestyle factors and intercepts of (**A**) total WMH, (**B**) MTLV-ratio, and (**C**) cognition as assessed with the PACC5. *** $p < 0.001$, ** $p < 0.01$, * $p < 0.05$, + $p < 0.1$. Exact FDR-corrected p-values are reported in the plot. Source data are provided as a Source Data file.

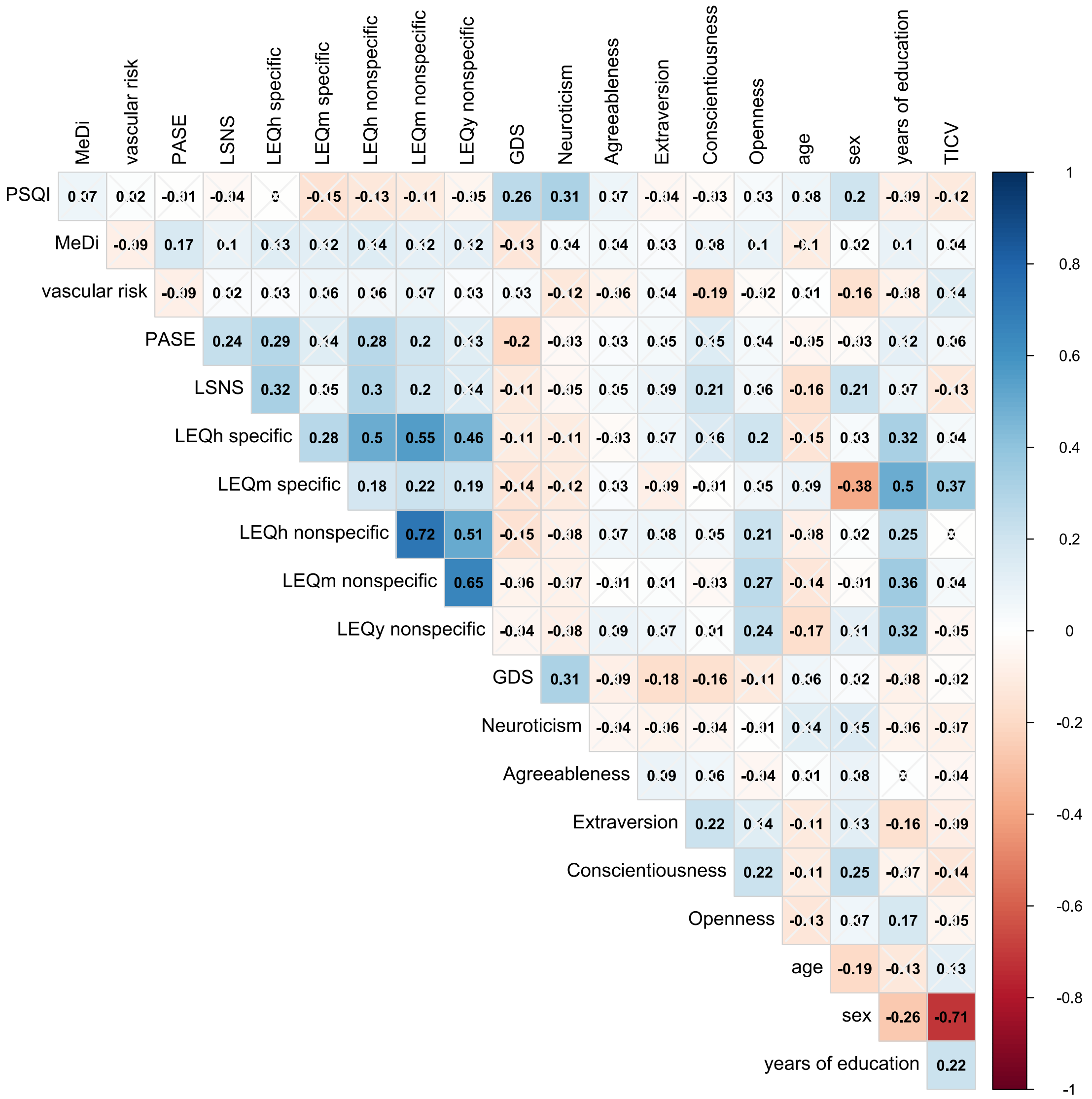

Supplementary Figure 7. Correlation matrix of lifestyle factors and demographic factors. FDR-corrected Spearman correlations are shown. Non-significant correlations are crossed out. Source data are provided as a Source Data file.

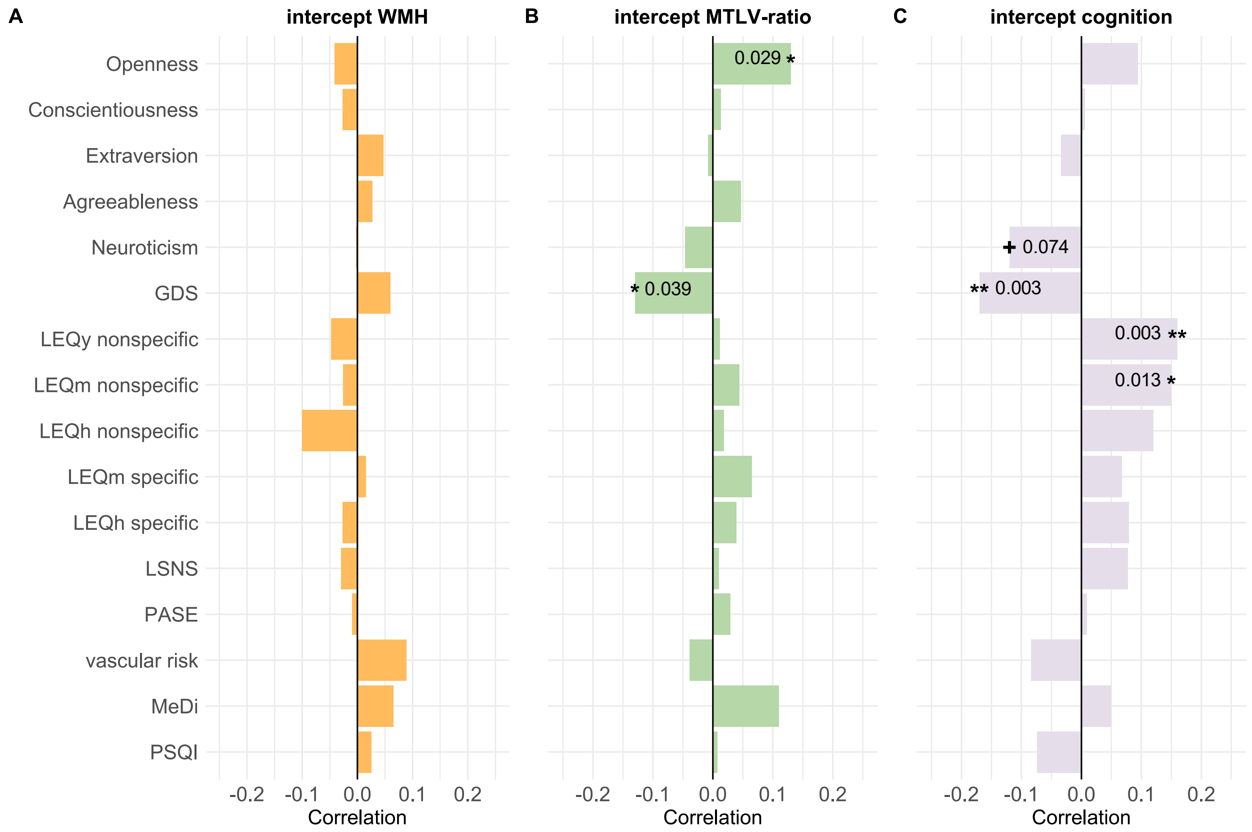

Supplementary Figure 8. FDR-corrected Spearman's correlation of latent intercepts with personality and modifiable lifestyle factors. Factor scores for latent intercepts were derived from the trivariate LGCM via regression-based method. Panels show relations between lifestyle factors and intercepts of (**A**) total WMH, (**B**) MTLV-ratio, and (**C**) cognition as assessed with the PACC5. *** $p < 0.001$, ** $p < 0.01$, * $p < 0.05$, + $p < 0.1$. Exact FDR-corrected p-values are reported in the plot. Source data are provided as a Source Data file.

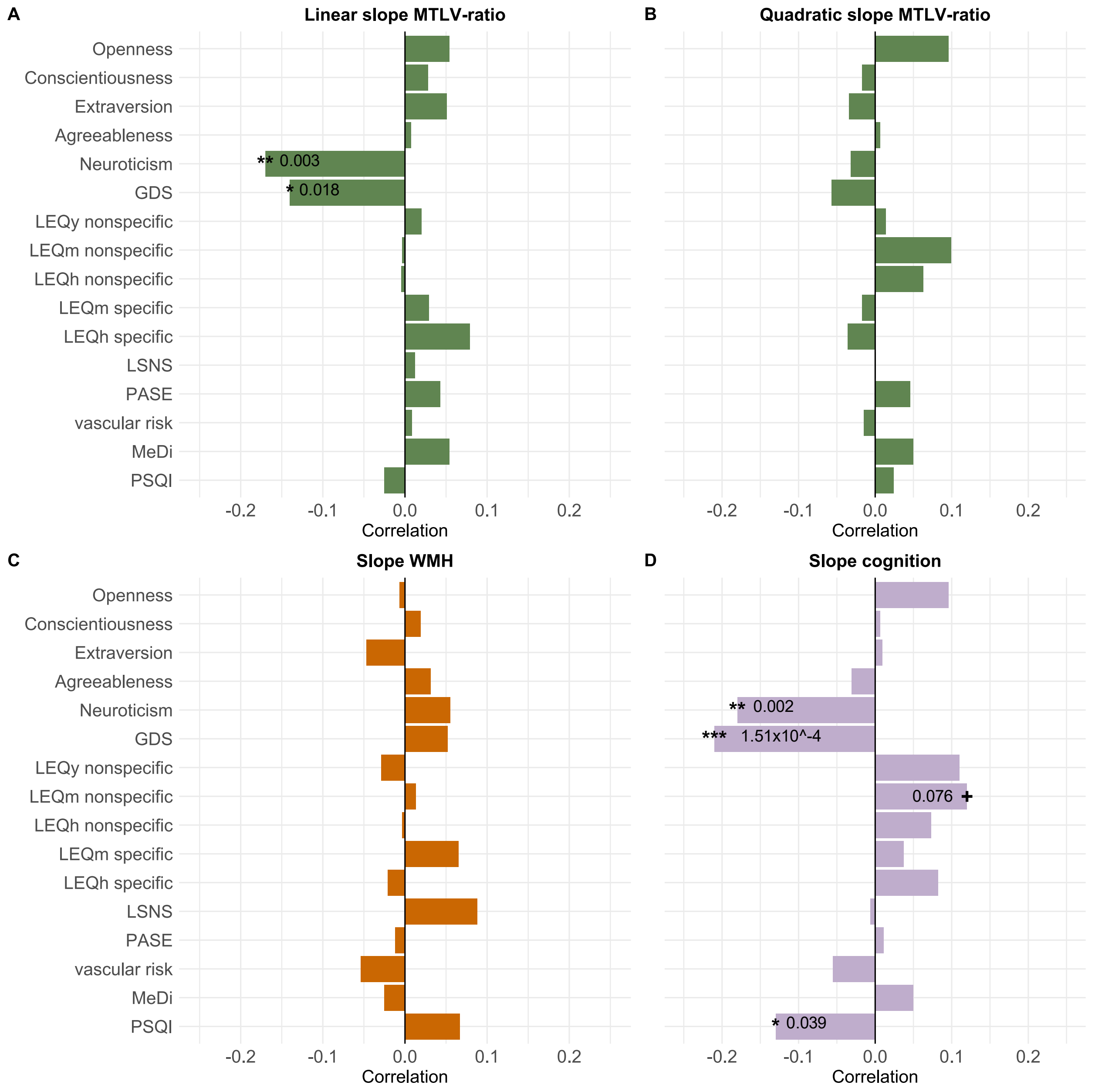

Supplementary Figure 9. FDR-corrected Spearman's correlation of latent slopes with modifiable lifestyle factors. Factor scores for latent slopes were derived from the trivariate LGCM via regression-based method. Panels show relations between lifestyle factors and latent slopes of (**A&B**) MTLV-ratio, (**C**) total WMH, and (**D**) cognition as assessed with the PACC5. *** $p < 0.001$, ** $p < 0.01$, * $p < 0.05$, + $p < 0.1$. Exact FDR-corrected p-values are reported in the plot. Source data are provided as a Source Data file.

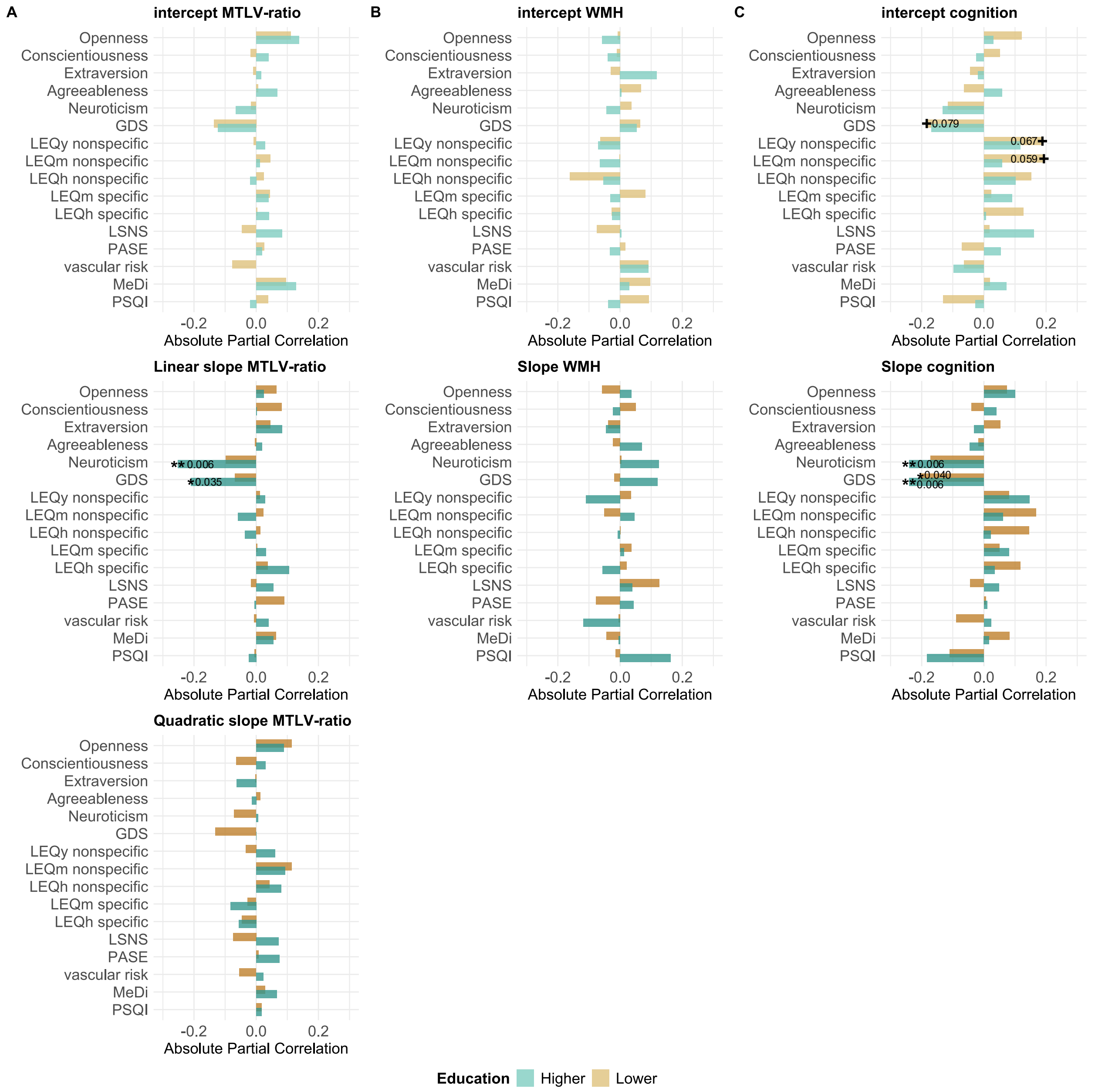

Supplementary Figure 10. Associations of neurocognitive domains with modifiable lifestyle factors across higher and lower educated individuals. Factor scores for latent intercepts and latent slopes were derived from a trivariate LGCM without correction for years of education via regression-based method. We used grouped partial Spearman's correlations to account for the effects of age, sex, and total intracranial volume (TICV). Turquois bars represent correlations for higher education (Ø 17.4 years), amber bars represent correlations for lower education (Ø 12.3 years). All correlations were FDR-corrected. Panels show relations between lifestyle factors, intercepts and slopes, respectively, of **(A)** MTLV-ratio, **(B)**, total WMH, and **(C)** cognition as assessed with the PACC5. ***p<0.001, **p<0.01, *p< 0.05, +p<0.1. Personality traits were

acquired via the Big Five Inventory BFI-10. GDS=geriatric depression scale. LEQ=lifetime experiences questionnaire assessed for three life periods y=young adulthood (13-30 years), m=midlife (30-65 years), h=late life (≥ 65 years or from retirement onward). LSNS=Lubben social network scale. PASE=physical activity scale for the elderly. MeDi=Mediterranean diet. PSQI=Pittsburgh sleep quality index (CAVE: by convention higher values denote lower sleep quality). We were particularly interested in education-associated changes within neurocognitive domains. Neither in lower nor higher educated individuals, WMH progression was linked to modifiable lifestyle factors. In line with previous studies[52], we found that irrespective of level of education, higher GDS scores were related to stronger PACC5 performance decline (lower: ρ=-0.204, p_{FDR}=0.040, 95%-CI [-0.32,-0.08]; higher ρ=-0.240, p_{FDR}=0.006, 95%-CI [-0.35,-0.12]). Moreover, higher GDS score tended to contribute to lower baseline PACC5 performance in lower educated people (ρ=-0.184, p_{FDR}=0.079, 95%-CI [-0.30,-0.06]). In individuals with higher education, higher GDS was also associated with steeper linear MTLV-ratio decline (ρ=-0.210, p_{FDR}=0.035, 95%-CI [-0.32,-0.09]). Higher levels of cognitively demanding leisure time activities in young (ρ=0.188, p_{FDR}=0.067, 95%-CI [0.07,0.30]) and middle-age (ρ=0.193, p_{FDR}=0.059, 95%-CI [0.07,0.31]) tended to contribute to increased baseline PACC5 performance in lower educated individuals, suggesting that engaging in cognitively stimulating behaviours may help compensate for cognitive disadvantages[53,54]. In individuals with higher levels of education, neuroticism was associated with MTLV-ratio decline (ρ=-0.252, p_{FDR}=0.006, 95%-CI [-0.36,-0.13]) and lower PACC5 performance changes (ρ=-0.241, p_{FDR}=0.006, 95%-CI [-0.35,-0.12]). Source data are provided as a Source Data file.

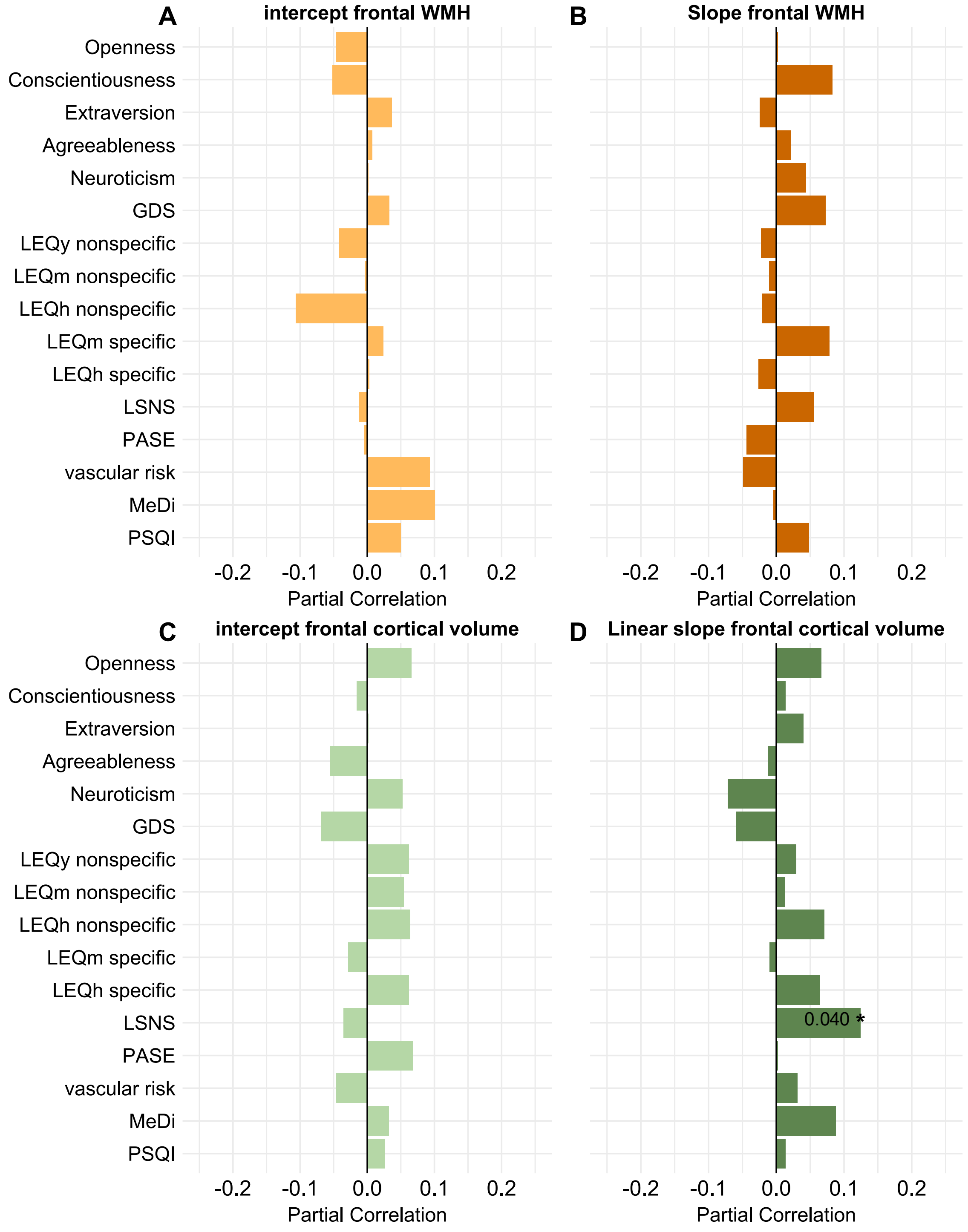

Supplementary Figure 11. FDR-corrected partial Spearman's correlation of latent intercepts and slopes from regional model (frontal) with personality and modifiable lifestyle factors. Factor scores for latent slopes were derived from the regional trivariate LGCM via regression-based method (frontal regions). We used partial Spearman's correlations to account for the effects of age, sex, years of education, and TICV. Panels show relations between personality and lifestyle factors and latent slopes of frontal WMH (**A&B**), and frontal cortical volume (**C&D**). *** p < 0.001, ** p < 0.01, * p < 0.05, + p < 0.1. Exact FDR-corrected p-values are reported in the plot. Source data are provided as a Source Data file.

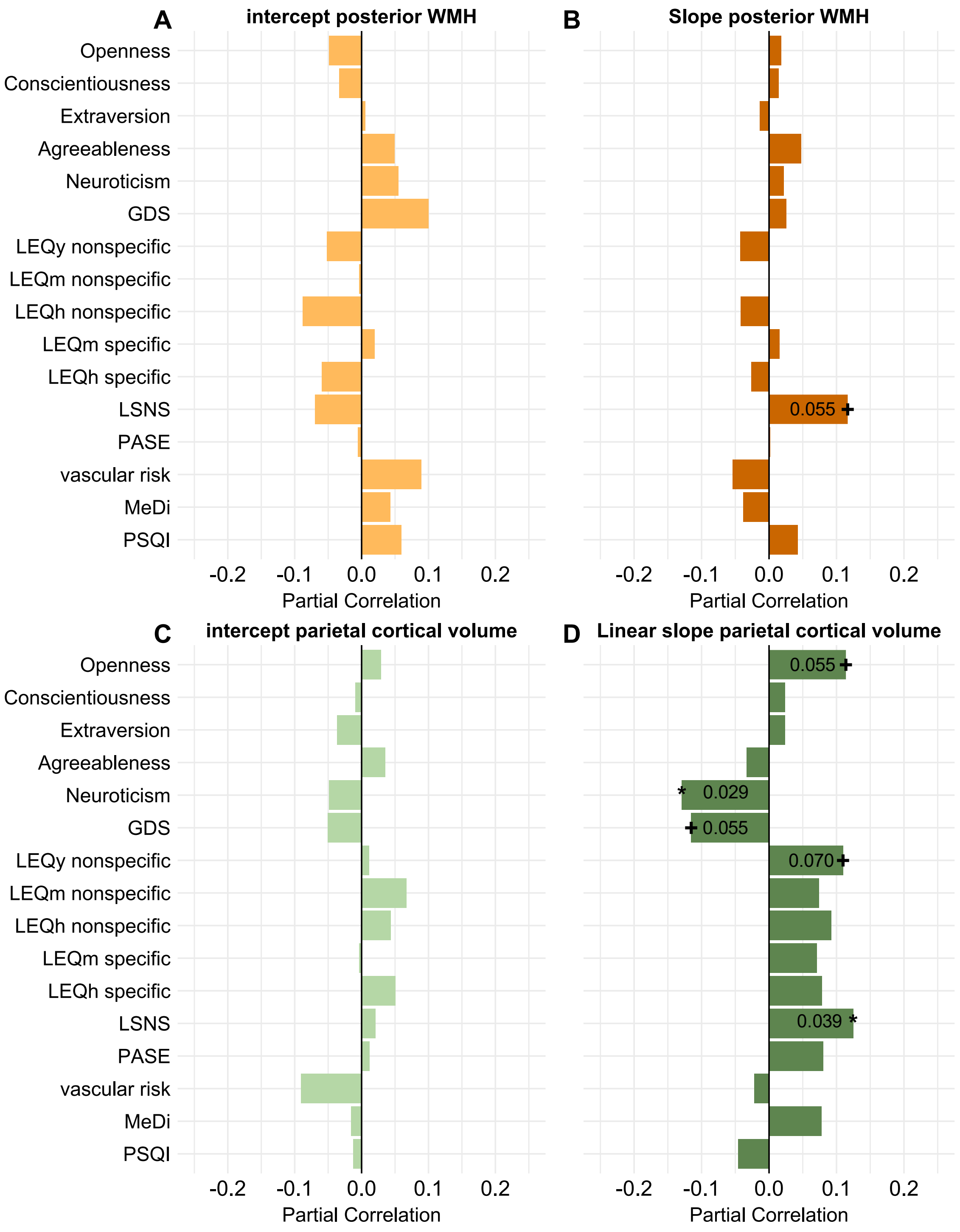

Supplementary Figure 12. FDR-corrected partial Spearman's correlation of latent intercepts and slopes from regional model (posterior) with personality and modifiable lifestyle factors. Factor scores for latent slopes were derived from the regional trivariate LGCM via regression-based method (posterior regions). We used partial Spearman's correlations to account for the effects of age, sex, years of education, and TICV. Panels show relations between personality and lifestyle factors and latent slopes of posterior WMH (**A&B**), and parietal cortical volume (**C&D**). *** $p < 0.001$, ** $p < 0.01$, * $p < 0.05$, + $p < 0.1$. Exact FDR-corrected p-values are reported in the plot. Source data are provided as a Source Data file.

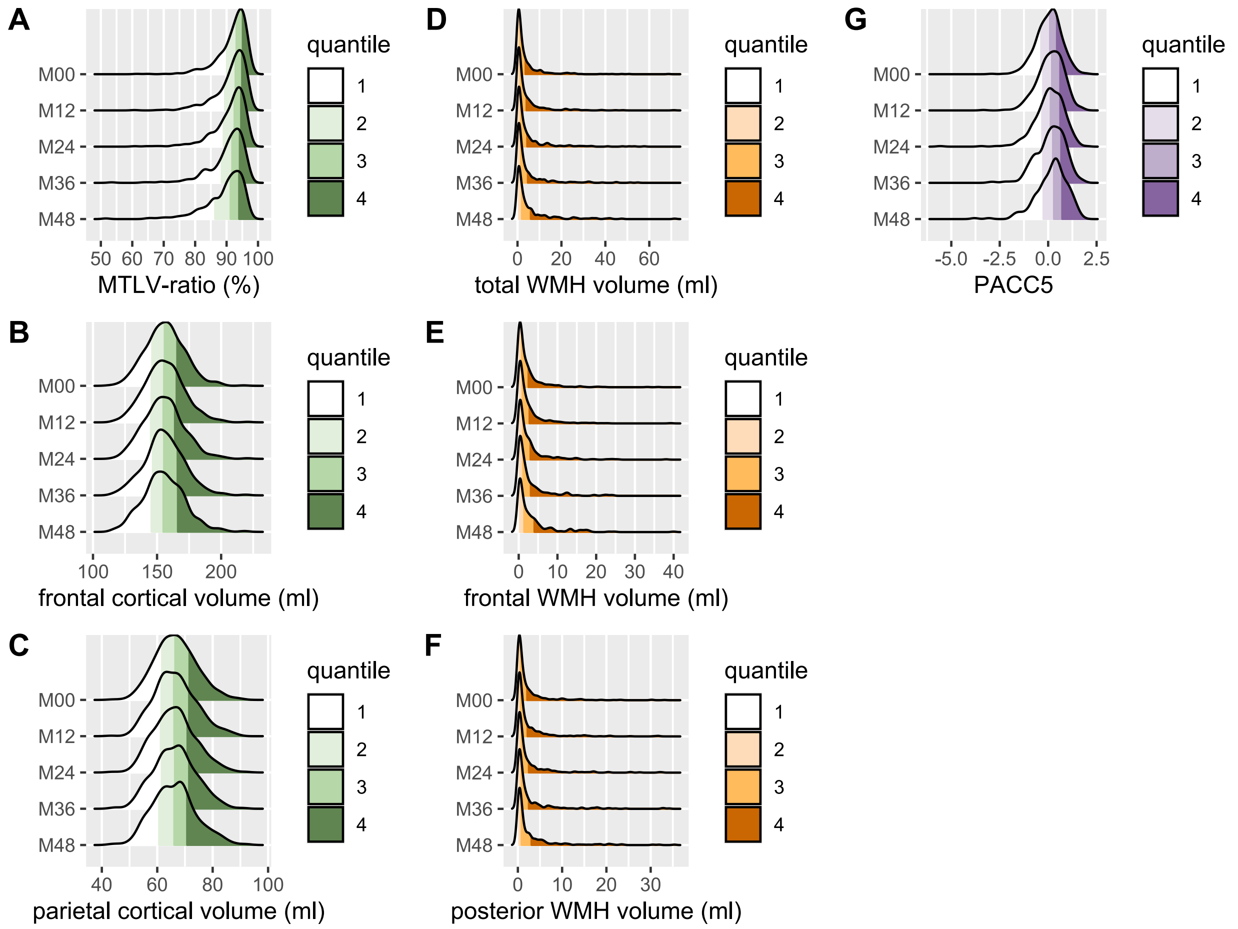

Supplementary Figure 13. Distribution plots of original data. Ridgeline density distribution of variables of the three neurocognitive domains of interest across the five assessment time points. Quantiles for each density are plotted. **(A-C)** Morphometric brain measures, incl. medial temporal lobe-to-ventricle ratio (MTLV-ratio), frontal and parietal cortical volumes. **(B-F)** White matter hyperintensity (WMH) volumes, incl. total, frontal, and posterior volumes. **(G)** Preclinical Alzheimer's Cognitive Composite Score (PACC5). Source data are provided as a Source Data file.

References

1. Yesavage JA, Brink TL, Rose TL, et al. Development and validation of a geriatric depression screening scale: A preliminary report. *J Psychiatr Res*. 1982;17(1):37-49. doi:10.1016/0022-3956(82)90033-4

2. Nöthlings U, Hoffmann K, Bergmann MM, Boeing H. Fitting Portion Sizes in a Self-Administered Food Frequency Questionnaire ,. *J Nutr*. 2007;137(12):2781-2786. doi:10.1093/jn/137.12.2781

3. Wesselman LMP, van Lent DM, Schröder A, et al. Dietary patterns are related to cognitive functioning in elderly enriched with individuals at increased risk for Alzheimer's disease. *Eur J Nutr*. 2021;60(2):849-860. doi:10.1007/s00394-020-02257-6

4. Märki A. *Entwicklung Und Evaluation Eines Beratungsinstrumentes Zur Förderung Der Körperlichen Aktivität Bei Älteren Menschen Unter Berücksichtigung Des Transtheoretischen Modells Der Verhlatensänderung*. University of Basel; 2004.

5. Washburn RA, Smith KW, Jette AM, Janney CA. The physical activity scale for the elderly (PASE): Development and evaluation. *J Clin Epidemiol*. 1993;46(2):153-162. doi:10.1016/0895-4356(93)90053-4

6. Buysse DJ, Reynolds CF, Monk TH, Berman SR, Kupfer DJ. The Pittsburgh sleep quality index: A new instrument for psychiatric practice and research. *Psychiatry Res*. 1989;28(2):193-213. doi:10.1016/0165-1781(89)90047-4

7. Lubben J, Blozik E, Gillmann G, et al. Performance of an Abbreviated Version of the Lubben Social Network Scale Among Three European Community-Dwelling Older Adult Populations. *Gerontologist*. 2006;46(4):503-513. doi:10.1093/geront/46.4.503

8. Valenzuela MJ, Sachdev PS. Assessment of complex mental activity across the lifespan: development of the Lifetime of Experiences Questionnaire (LEQ). *Psychol Med*. 2007;37(7):1015-1025. doi:10.1017/S003329170600938X

9. Rammstedt B, John OP. Measuring personality in one minute or less: A 10-item short version of the Big Five Inventory in English and German. *J Res Pers*. 2007;41(1):203-212. doi:10.1016/j.jrp.2006.02.001

10. Vogelgsang J, Hansen N, Stark M, et al. Plasma amyloid beta X-42/X-40 ratio and cognitive decline in suspected early and preclinical Alzheimer's disease. *Alzheimer's & Dementia*. 2024;20(8):5132-5142. doi:10.1002/alz.13909

11. Mengel D, Soter E, Ott JM, et al. Blood biomarkers confirm subjective cognitive decline (SCD) as a distinct molecular and clinical stage within the NIA-AA framework of Alzheimer´s disease. *Mol Psychiatry*. 2025;30(7):3150-3159. doi:10.1038/s41380-025-03021-0

12. Doecke JD, Pérez-Grijalba V, Fandos N, et al. Total Aβ 42 /Aβ 40 ratio in plasma predicts amyloid-PET status, independent of clinical AD diagnosis. *Neurology*. 2020;94(15):E1580-E1591. doi:10.1212/WNL.0000000000009240

13. Hu Y, Kirmess KM, Meyer MR, et al. Assessment of a Plasma Amyloid Probability Score to Estimate Amyloid Positron Emission Tomography Findings Among Adults With Cognitive Impairment. *JAMA Netw Open*. 2022;5(4):e228392. doi:10.1001/jamanetworkopen.2022.8392

14. West T, Kirmess KM, Meyer MR, et al. A blood-based diagnostic test incorporating plasma Aβ42/40 ratio, ApoE proteotype, and age accurately identifies brain amyloid status: findings from a multi cohort validity analysis. *Mol Neurodegener*. 2021;16(1):30. doi:10.1186/s13024-021-00451-6

15. Palmqvist S, Stomrud E, Cullen N, et al. An accurate fully automated panel of plasma biomarkers for Alzheimer's disease. *Alzheimer's and Dementia*. 2023;19(4):1204-1215. doi:10.1002/alz.12751

16. Jessen F, Spottke A, Boecker H, et al. Design and first baseline data of the DZNE multicenter observational study on predementia Alzheimer's disease (DELCODE). *Alzheimers Res Ther*. 2018;10(1):1-10. doi:10.1186/s13195-017-0314-2

17. Cacciaglia R, Molinuevo JL, Falcón C, et al. Effects of APOE -ε4 allele load on brain morphology in a cohort of middle-aged healthy individuals with enriched genetic risk for Alzheimer's disease. *Alzheimer's & Dementia*. 2018;14(7):902-912. doi:10.1016/j.jalz.2018.01.016

18. Schuff N, Woerner N, Boreta L, et al. MRI of hippocampal volume loss in early Alzheimer's disease in relation to ApoE genotype and biomarkers. *Brain*. 2008;132(4):1067-1077. doi:10.1093/brain/awp007

19. Gorbach T, Pudas S, Bartrés-Faz D, et al. Longitudinal association between hippocampus atrophy and episodic-memory decline in non-demented APOE ε4 carriers. *Alzheimer's & Dementia: Diagnosis, Assessment & Disease Monitoring*. 2020;12(1):1-9. doi:10.1002/dad2.12110

20. Aschenbrenner AJ, Li Y, Henson RL, et al. Comparison of plasma and CSF biomarkers in predicting cognitive decline. *Ann Clin Transl Neurol*. 2022;9(11):1739-1751. doi:10.1002/acn3.51670

21. van Arendonk J, Wolters FJ, Neitzel J, et al. Plasma neurofilament light chain in relation to 10-year change in cognition and neuroimaging markers: a population-based study. *Geroscience*. 2023;46(1):57-70. doi:10.1007/s11357-023-00876-5

22. Khalil M, Pirpamer L, Hofer E, et al. Serum neurofilament light levels in normal aging and their association with morphologic brain changes. *Nat Commun*. 2020;11(1):812. doi:10.1038/s41467-020-14612-6

23. Meeker KL, Butt OH, Gordon BA, et al. Cerebrospinal fluid neurofilament light chain is a marker of aging and white matter damage. *Neurobiol Dis*. 2022;166:105662. doi:10.1016/j.nbd.2022.105662

24. Mattson MP, Arumugam T V. Hallmarks of Brain Aging: Adaptive and Pathological Modification by Metabolic States. *Cell Metab*. *Cell Press*. 2018;27(6):1176-1199. doi:10.1016/j.cmet.2018.05.011

25. Idland AV, Sala-Llonch R, Borza T, et al. CSF neurofilament light levels predict hippocampal atrophy in cognitively healthy older adults. *Neurobiol Aging*. 2017;49:138-144. doi:10.1016/j.neurobiolaging.2016.09.012

26. Malek-Ahmadi M, Su Y, Ghisays V, et al. Plasma NfL is associated with the APOE ε4 allele, brain imaging measurements of neurodegeneration, and lower recall memory scores in cognitively unimpaired late-middle-aged and older adults. *Alzheimers Res Ther*. 2023;15(1). doi:10.1186/s13195-023-01221-w

27. Appelman APA, Exalto LG, van der Graaf Y, Biessels GJ, Mali WPTM, Geerlings MI. White Matter Lesions and Brain Atrophy: More than Shared Risk Factors? A Systematic Review. *Cerebrovascular Diseases*. 2009;28(3):227-242. doi:10.1159/000226774

28. Godin O, Maillard P, Crivello F, et al. Association of White-Matter Lesions with Brain Atrophy Markers: The Three-City Dijon MRI Study. *Cerebrovascular Diseases*. 2009;28(2):177-184. doi:10.1159/000226117

29. Cox SR, Lyall DM, Ritchie SJ, et al. Associations between vascular risk factors and brain MRI indices in UK Biobank. *Eur Heart J*. 2019;40(28):2290-2299. doi:10.1093/eurheartj/ehz100

30. Fiford CM, Manning EN, Bartlett JW, et al. White matter hyperintensities are associated with disproportionate progressive hippocampal atrophy. *Hippocampus*. 2017;27(3):249-262. doi:10.1002/hipo.22690

31. Bernal J, Menze I, Yakupov R, et al. Longitudinal evidence for a mutually reinforcing relationship between white matter hyperintensities and cortical thickness in cognitively unimpaired older adults. *Alzheimers Res Ther*. 2024;16(1):240. doi:10.1186/s13195-024-01606-5

32. Aamand R, Rasmussen PM, Andersen KS, et al. Cerebral microvascular changes in healthy carriers of the APOE-ε4 Alzheimer's disease risk gene. Klann E, ed. *PNAS Nexus*. 2024;3(9):1-15. doi:10.1093/pnasnexus/pgae369

33. Driscoll I, Davatzikos C, An Y, et al. Longitudinal pattern of regional brain volume change differentiates normal aging from MCI. *Neurology*. 2009;72(22):1906-1913. doi:10.1212/WNL.0b013e3181a82634

34. Carmichael OT, Kuller LH, Lopez OL, et al. Cerebral ventricular changes associated with transitions between normal cognitive function, mild cognitive impairment, and dementia. *Alzheimer Dis Assoc Disord*. 2007;21(1):14-24. doi:10.1097/WAD.0b013e318032d2b1

35. Mizuno K, Wakai M, Takeda A, Sobue G. Medial temporal atrophy and memory impairment in early stage of Alzheimer's disease: an MRI volumetric and memory assessment study. *J Neurol Sci*. 2000;173(1):18-24. doi:10.1016/S0022-510X(99)00289-0

36. Macdonald KE, Bartlett JW, Leung KK, Ourselin S, Barnes J. The Value of Hippocampal and Temporal Horn Volumes and Rates of Change in Predicting Future Conversion to AD. *Alzheimer Dis Assoc Disord*. 2013;27(2):168-173. doi:10.1097/WAD.0b013e318260a79a

37. Bartos A, Gregus D, Ibrahim I, Tintěra J. Brain volumes and their ratios in Alzheimer´s disease on magnetic resonance imaging segmented using Freesurfer 6.0. *Psychiatry Res Neuroimaging*. 2019;287(December 2018):70-74. doi:10.1016/j.pscychresns.2019.01.014

38. Schoemaker D, Buss C, Pietrantonio S, et al. The hippocampal-to-ventricle ratio (HVR): Presentation of a manual segmentation protocol and preliminary evidence. *Neuroimage*. 2019;203(April):116108. doi:10.1016/j.neuroimage.2019.116108

39. Coupé P, Manjón J V., Mansencal B, Tourdias T, Catheline G, Planche V. Hippocampal-amygdalo-ventricular atrophy score: Alzheimer disease detection using normative and pathological lifespan models. *Hum Brain Mapp*. 2022;43(10):3270-3282. doi:10.1002/hbm.25850

40. Zahodne LB, Wall MM, Schupf N, et al. Late-life memory trajectories in relation to incident dementia and regional brain atrophy. *J Neurol*. 2015;262(11):2484-2490. doi:10.1007/s00415-015-7871-8

41. Bangen KJ, Preis SR, Delano-Wood L, et al. Baseline White Matter Hyperintensities and Hippocampal Volume are Associated With Conversion From Normal Cognition to Mild Cognitive Impairment in the Framingham Offspring Study. *Alzheimer Dis Assoc Disord*. 2018;32(1):50-56. doi:10.1097/WAD.0000000000000215

42. Prosser L, Macdougall A, Sudre CH, et al. Predicting Cognitive Decline in Older Adults Using Baseline Metrics of AD Pathologies, Cerebrovascular Disease, and Neurodegeneration. *Neurology*. 2023;100(8):E834-E845. doi:10.1212/WNL.0000000000201572

43. Jokinen H, Koikkalainen J, Laakso HM, et al. Global Burden of Small Vessel Disease–Related Brain Changes on MRI Predicts Cognitive and Functional Decline. *Stroke*. 2020;51(1):170-178. doi:10.1161/STROKEAHA.119.026170

44. Kamal F, Morrison C, Maranzano J, Zeighami Y, Dadar M. White Matter Hyperintensity Trajectories in Patients With Progressive and Stable Mild Cognitive Impairment. *Neurology*. 2023;101(8):1-31. doi:10.1212/WNL.0000000000207514

45. Dadar M, Maranzano J, Ducharme S, Collins DL. White matter in different regions evolves differently during progression to dementia. *Neurobiol Aging*. 2019;76:71-79. doi:10.1016/j.neurobiolaging.2018.12.004

46. de Havenon A, Sheth KN, Yeatts SD, Turan TN, Prabhakaran S. White matter hyperintensity progression is associated with incident probable dementia or mild cognitive impairment. *Stroke Vasc Neurol*. 2022;7(4):364-366. doi:10.1136/svn-2021-001357

47. Hassenstab J, Ruvolo D, Jasielec M, Xiong C, Grant E, Morris JC. Absence of practice effects in preclinical Alzheimer's disease. *Neuropsychology*. 2015;29(6):940-948. doi:10.1037/neu0000208

48. Jutten RJ, Grandoit E, Foldi NS, et al. Lower practice effects as a marker of cognitive performance and dementia risk: A literature review. *Alzheimer's & Dementia: Diagnosis, Assessment & Disease Monitoring*. 2020;12(1):1-12. doi:10.1002/dad2.12055

49. De Simone MS, Perri R, Rodini M, et al. A Lack of Practice Effects on Memory Tasks Predicts Conversion to Alzheimer Disease in Patients With Amnestic Mild Cognitive Impairment. *J Geriatr Psychiatry Neurol*. 2021;34(6):582-593. doi:10.1177/0891988720944244

50. Machulda MM, Pankratz VS, Christianson TJ, et al. Practice Effects and Longitudinal Cognitive Change in Normal Aging vs. Incident Mild Cognitive Impairment and Dementia in The Mayo Clinic Study of Aging. *Clin Neuropsychol*. 2013;27(8):1247-1264. doi:10.1080/13854046.2013.836567

51. Samaroo A, Amariglio RE, Burnham S, et al. Diminished Learning Over Repeated Exposures (LORE) in preclinical Alzheimer's disease. *Alzheimer's & Dementia: Diagnosis, Assessment & Disease Monitoring*. 2020;12(1):1-10. doi:10.1002/dad2.12132

52. Santos NC, Costa PS, Cunha P, et al. Clinical, physical and lifestyle variables and relationship with cognition and mood in aging: a cross-sectional analysis of distinct educational groups. *Front Aging Neurosci*. 2014;6(FEB):1-15. doi:10.3389/fnagi.2014.00021

53. Feng Y, Jia S, Zhao W, et al. Independent and Joint Associations of Socioeconomic Status and Lifestyle behaviors with Cognitive Impairment among Elderly Chinese Population. *Journal of Prevention of Alzheimer's Disease*. 2024;5(11):1513-1522. doi:10.14283/jpad.2024.127

54. Wang K, Fang Y, Zheng R, et al. Associations of socioeconomic status and healthy lifestyle with incident dementia and cognitive decline: two prospective cohort studies. *EClinicalMedicine*. 2024;76:102831. doi:10.1016/j.eclinm.2024.102831
