# Supplementary material for: Joint trajectories of brain atrophy, white matter hyperintensities and cognition quantify brain maintenance
Source: Nat Commun. 2026 Jul 4;17:5846. doi: 10.1038/s41467-026-74957-2 (PMC13332871; doi:10.1038/s41467-026-74957-2)
Supplement: Supplementary file 2 — Reporting Summary [file 41467_2026_74957_MOESM2_ESM.pdf]

nature portfolio

Corresponding author(s): Inga Menze

Last updated by author(s): Apr 18, 2026

Reporting Summary

Nature Portfolio wishes to improve the reproducibility of the work that we publish. This form provides structure for consistency and transparency in reporting. For further information on Nature Portfolio policies, see our Editorial Policies and the Editorial Policy Checklist.

Statistics

For all statistical analyses, confirm that the following items are present in the figure legend, table legend, main text, or Methods section.

n/a	Confirmed	
☐	☒	The exact sample size (*n*) for each experimental group/condition, given as a discrete number and unit of measurement
☐	☒	A statement on whether measurements were taken from distinct samples or whether the same sample was measured repeatedly
☐	☒	The statistical test(s) used AND whether they are one- or two-sided<br>*Only common tests should be described solely by name; describe more complex techniques in the Methods section.*
☐	☒	A description of all covariates tested
☐	☒	A description of any assumptions or corrections, such as tests of normality and adjustment for multiple comparisons
☐	☒	A full description of the statistical parameters including central tendency (e.g. means) or other basic estimates (e.g. regression coefficient) AND variation (e.g. standard deviation) or associated estimates of uncertainty (e.g. confidence intervals)
☐	☒	For null hypothesis testing, the test statistic (e.g. *F*, *t*, *r*) with confidence intervals, effect sizes, degrees of freedom and *P* value noted<br>*Give P values as exact values whenever suitable.*
☒	☐	For Bayesian analysis, information on the choice of priors and Markov chain Monte Carlo settings
☒	☐	For hierarchical and complex designs, identification of the appropriate level for tests and full reporting of outcomes
☐	☒	Estimates of effect sizes (e.g. Cohen's *d*, Pearson's *r*), indicating how they were calculated

Our web collection on statistics for biologists contains articles on many of the points above.

Software and code

Policy information about availability of computer code

Data collection	The R code implementing the trivariate latent growth curve model (lavaan syntax), factor-score extraction, and robust regression for the brain maintenance index is openly available at https://github.com/neuroprognosis/brain-maintenance-lgcm. The repository contains a synthetic example dataset reproducing the analysis pipeline end-to-end so that the framework is usable on alternative cohorts without access to DELCODE raw data.
Data analysis	MRI processing was performed using Freesurfer (v7.1) and LST-AI (Wiltgen et al., 2024).<br>R (v4.2.3) was used for statistical analyses and visualisation, using packages lavaan (v0.6-16), rstatix (v0.7.2), ppcor (v1.1), ggplot2 (v3.4.2), robustbase (v0.99.0), and semPlot (v1.1.6). Methylation data for DunedinPACE was processed via meffil (v1.3.8) and dnaMethyAge (v0.2.0).

For manuscripts utilizing custom algorithms or software that are central to the research but not yet described in published literature, software must be made available to editors and reviewers. We strongly encourage code deposition in a community repository (e.g. GitHub). See the Nature Portfolio guidelines for submitting code & software for further information.

Data

Policy information about availability of data

All manuscripts must include a data availability statement. This statement should provide the following information, where applicable:

- Accession codes, unique identifiers, or web links for publicly available datasets
- A description of any restrictions on data availability
- For clinical datasets or third party data, please ensure that the statement adheres to our policy

The raw data collected in the study "DELCODE—DZNE-Longitudinal Cognitive Impairment and Dementia Study (BN012)" cannot be made openly available without violation of the data protection concept of the DZNE. Access to the relevant study data can be obtained by submitting an application to the Clinical Research Platform of the DZNE. The template for the application for the submission of data and biomaterial samples is available on the DZNE homepage (https://www.dzne.de/en/research/research-areas/clinical-research/for-researchers/). The expected timeframe for response to access requests is 1 month. Access will be granted for 10 years. Source data are provided with this paper.

Research involving human participants, their data, or biological material

Policy information about studies with human participants or human data. See also policy information about sex, gender (identity/presentation), and sexual orientation and race, ethnicity and racism.

Reporting on sex and gender	Sex was self-reported by participants (287 females, 256 males in the analysis sample). Disaggregated sex and gender information was not collected.<br>Sex was used as a covariate in our analyses. No sex-specific analyses were conducted, as the goal of our study was a broader understanding of the interrelationships between white matter hyperintensities, ageing-related atrophy, and cognitive ageing.
Reporting on race, ethnicity, or other socially relevant groupings	No race, ethnicity, or other socially relevant grouping variables - except for years of education - were used in the analyses. Years of education, age, and sex were controlled for in our analysis. For a supplementary analysis, years of education were also used as a grouping variable.
Population characteristics	see below
Recruitment	Cognitively unimpaired individuals in this study were recruited by standardized public advertisement. SCD patients of DELCODE that were also considered cognitively unimpared were referrals, including self-referrals, to the participating memory clinics of the German Center for Neuodegenerative Diseases. In the manuscript, we refer to the DELCODE baseline paper (Jessen et al., 2018) for detailed information.
Ethics oversight	Ethics committees of the medical faculties of all participating sites, Berlin (Charité, University Medicine), Bonn, Cologne, Göttingen, Magdeburg, Munich (Ludwig-Maximilians-University), Rostock, and Tübingen, gave ethical approval for this work. The ethics committee of the medical faculty of the University of Bonn led and coordinated the process (trial registration number 117/13).

Note that full information on the approval of the study protocol must also be provided in the manuscript.

Field-specific reporting

Please select the one below that is the best fit for your research. If you are not sure, read the appropriate sections before making your selection.

☐ Life sciences ☒ Behavioural & social sciences ☐ Ecological, evolutionary & environmental sciences

For a reference copy of the document with all sections, see nature.com/documents/nr-reporting-summary-flat.pdf

Behavioural & social sciences study design

All studies must disclose on these points even when the disclosure is negative.

Study description	DELCODE (DZNE Longitudinal Cognitive Impairment and Dementia Study; Jessen et al., 2018) is an observational longitudinal memory clinic-based multicentre study in Germany conducted by the German Centre for Neurodegenerative Diseases (DZNE). Quantative data was analysed in this study.
Research sample	543 individuals who attend at least two annual visits were included in the analyses. Participants visited annually over a 4-year period (baseline assessment and 4 follow-ups). All provided relevant demographic data used in the study (sex, age, years of education: 52.85% female; mean age 69.99 ± 5.87 years; mean years of education: 14.82 ± 2.92 years). The DELCODE study sample comes from the general German population over 60 years. DELCODE is apparently suitable for examining brain maintenance, due to the availability of comprehensive neuropsychological data (here we used PACC5 scores), imaging data, self-report data on a variety of personality and lifestyle factors (here we used late-life depressive symptoms, Mediterranean diet, physical activity, sleep quality, social network, lifetime experiences, and cardiovascular risk factors), and Alzheimer's biomarkers or genetic risk factors (APOE4). In this study Plasma Aβ42/40, Plasma pTau181, Plasma NfL, and APOE4 were used for supplementary analyses (Plasma levels of Aβ42/40: n = 417; Plasma pTau181: n = 469; Plasa Nfl: n = 512; APOE4: carrier = 150; non-carrier = 388; missing = 5). DunedinPACE as molecular marker for biological ageing used for validation analyses was available for n = 502.

Sampling strategy	Initially, 1078 participants were enrolled into 5 groups at baseline (stratified sampling): Normal controls (NC), subjective cognitive decline (SCD), mild cognitive impairment (MCI), dementia due to Alzheimer's disease (AD), and AD patient relatives (ADR). For this study, only cognitively unimpaired individuals (NC,ADR,SCD) were used.<br>Generally, DELCODE was powered for the univariate detection of significant predictors for cognitive decline in subjects with SCD. In a European multicenter memory clinic study the frequency of AD type CSF in subjects with SCD was 50%. Due to slight differences in the definition of SCD (i.e. inclusion of subjects reporting worries about other than memory decline in DELCODE) a frequency of 40% individuals in the SCD group was estimated to display evidence for amyloid deposition in the CSF. Another study (van Harten et al.,2011) reported a hazard ratio (HR) of 15 for MCI/dementia (evidenced by episodic memory decline) in memory clinic patients with SCD and Aβ42 reduction in the CSF with a mean observation period of 4 years. DELCODE employed a more conservative estimation. The assumption for an univariate predictor for episodic memory decline in preclinical AD over 5 years was an odds ratio of 3. With these assumptions (40% SCD subjects with preclinical AD, OR=3, 5 year follow-up, 10% drop-out), 300 patients with SCD were required to identify a predictor of episodic memory decline with 80% power. The sizes of the other groups were defined to be sufficiently large for comparison with the SCD group and to be feasible to recruit within the DZNE multicenter structure.
Data collection	Trained researchers administered the neuropsychological pen-and-paper tests, and conducted MRI scanning. They were not aware of the primary study hypothesis.
Timing	Data included in this study was collected between 2014 and 2023
Data exclusions	To get robust estimates for latent linear change rates and baseline levels in our latent growth curve model analyses, we restricted the sample to individuals with at least two visits (543 participants from 722 eligible cognitively unimpaired individuals intially recruited at baseline for DELCODE). Prior fitting the latent growth curve model, we identified and removed outliers that were above Q3 + 1.5×IQR or below Q1 - 1.5×IQR of the median for WMH, MTLV-ratio, and PACC5 performance, respectively (affected n=38 individuals), separately for each assessment time point. To study the unique effects of WMH and MTLV-ratio latent slopes on the latent slopes of PACC5 we used robust multiple linear regression.
Non-participation	Decline of participation was not recorded. From the initially recruited sample, 138 participants only visited at baseline and then discontinued the study for various reasons (incl. participant or relative wanted termination; participant is in nursing home; other disease that prevents further participation; decision of the responsible doctor; participant moved away; contact lost; participant is bedridden; other reasons, e.g. death)
Randomization	There were no experimental groups used for allocation.

Reporting for specific materials, systems and methods

We require information from authors about some types of materials, experimental systems and methods used in many studies. Here, indicate whether each material, system or method listed is relevant to your study. If you are not sure if a list item applies to your research, read the appropriate section before selecting a response.

Materials & experimental systems

n/a	Involved in the study	
☒	☐	Antibodies
☒	☐	Eukaryotic cell lines
☒	☐	Palaeontology and archaeology
☒	☐	Animals and other organisms
☒	☐	Clinical data
☒	☐	Dual use research of concern
☒	☐	Plants

Methods

n/a	Involved in the study	
☒	☐	ChIP-seq
☒	☐	Flow cytometry
☐	☒	MRI-based neuroimaging

Plants

Seed stocks	*Report on the source of all seed stocks or other plant material used. If applicable, state the seed stock centre and catalogue number. If plant specimens were collected from the field, describe the collection location, date and sampling procedures.*
Novel plant genotypes	*Describe the methods by which all novel plant genotypes were produced. This includes those generated by transgenic approaches, gene editing, chemical/radiation-based mutagenesis and hybridization. For transgenic lines, describe the transformation method, the number of independent lines analyzed and the generation upon which experiments were performed. For gene-edited lines, describe the editor used, the endogenous sequence targeted for editing, the targeting guide RNA sequence (if applicable) and how the editor was applied.*
Authentication	*Describe any authentication procedures for each seed stock used or novel genotype generated. Describe any experiments used to assess the effect of a mutation and, where applicable, how potential secondary effects (e.g. second site T-DNA insertions, mosiacism, off-target gene editing) were examined.*

Magnetic resonance imaging

Experimental design

Design type	Structural analysis
Design specifications	n/a
Behavioral performance measures	n/a

Acquisition

Imaging type(s)	Structural
Field strength	3.0 T
Sequence & imaging parameters	- T1-weighted MPRAGE (full head coverage; 3D acquisition, GRAPPA factor 2, 1 mm^3 isotropic, 256 × 256 px, 192 sagittal slices, TR/TE/TI 2500/4.33/1100 ms, FA 7°)<br>- T2-weighted FLAIR (full head coverage; 1 mm^3 isotropic, 256 × 256 px, 192 sagittal slices, TR/TE/TI 5000/394/1800 ms)
Area of acquisition	T1-weighted and T2-weighted FLAIR were both acquired whole-brain

Diffusion MRI ☐ Used ☒ Not used

Preprocessing

Preprocessing software	- Freesurfer's longitudinal pipeline (v7.1) for segmentations from T1w MPRAGE images<br>- LST-AI (Wiltgen et al., 2024) for white matter hyperintensities segmentation
Normalization	n/a
Normalization template	n/a
Noise and artifact removal	During the imaging session, images were visually quality checked for artefacts (e.g. motion). In case of suboptimal imaging quality, the respective sequence was rerun.<br>Automatically generated segmentation and parcellation maps from Freesurfer were overlaid on the corresponding MPRAGE images and visually inspected by trained raters to identify and discard gross errors or abnormalities (e.g., topological defects, mislabelled cortical regions, or failed skull stripping).<br>WMH segmentations from LST-AI were post-processed to reduce false positive segmentation of WMH. Post-processing also involved excluding lesions located outside the supratentorial white matter, specifically those identified in cortical and subcortical grey matter, the brainstem, the choroid plexus, or the septum pellucidum.
Volume censoring	n/a

Statistical modeling & inference

Model type and settings	n/a
Effect(s) tested	n/a

Specify type of analysis: ☐ Whole brain ☐ ROI-based ☒ Both

Anatomical location(s)	- total, frontal, posterior (parietooccipital) whole-brain white matter hyperintensities volumes were used<br>- aggregated volumes of hippocampus, entorhinal cortex, parahippocampal cortex, amygdala, and inferior lateral ventricles, proposed to be sensitive to distinguish between normal and pathological ageing, were used from Freesurfer's longitudinal volume estimates to determine the medial temporal lobe-to-ventricle ratio (MTLV-ratio)<br>- frontal and posterior cortical volume according to Deskian et al. (2006) were used from Freesurfer's longitudinal volume estimates
Statistic type for inference (See Eklund et al. 2016)	n/a
Correction	n/a

Models & analysis

n/a | Involved in the study

- [x] n/a [ ] Functional and/or effective connectivity
- [x] n/a [ ] Graph analysis
- [ ] n/a [x] Multivariate modeling or predictive analysis

Multivariate modeling and predictive analysis

We leveraged latent growth curve modelling (LGCM) via lavaan to estimate growth factors—baseline levels (latent intercepts) and change (latent slopes)—in three domains of interest (white matter hyperintensities [log10-transformed], medial temporal lobe-to-ventricle ratio or frontal or posterior volume [Box-Cox transformed], PACC5 performance [Yeo-Johnson transformed]), while concurrently accounting for covariate effects. All latent intercepts and latent slopes were adjusted for effects of age, sex, years of education. Latent intercepts and latent slopes of WMH and MTLV-ratio/frontal or posterior volume were moreover corrected for TICV. All variables were z-scored (pooled across time points) before entering the model.

We modelled linear latent slopes and in case of MTLV-ratio linear and quadratic latent slopes. Models were fitted using robust maximum likelihood estimator (MLR) and missing data were handled using Full Information Maximum Likelihood Estimation (FIML). We ascertained the assumption of data missing at random via Little's missing completely at random test ($\chi2(1466) = 1544.30$, $p = 0.076$). Model fit was evaluated by the χ^2 test, Comparative Fit Index (CFI), root mean square error of approximation (RMSEA), and standardized root mean square residuals (SRMR). We extracted regression-based factor score estimates of latent slopes for each individual for post-hoc analyses.
