# Supplementary material for: Joint trajectories of brain atrophy, white matter hyperintensities and cognition quantify brain maintenance
Source: Nat Commun. 2026 Jul 4;17:5846. doi: 10.1038/s41467-026-74957-2 (PMC13332871; doi:10.1038/s41467-026-74957-2)
Supplement: Supplementary file 3 — Transparent Peer Review file [file 41467_2026_74957_MOESM3_ESM.pdf]

nature portfolio

Peer Review File

Joint trajectories of brain atrophy, white matter hyperintensities and cognition quantify brain maintenance

Corresponding Author: Ms Inga Menze

This file contains all reviewer reports in order by version, followed by all author rebuttals in order by version.

Version 0:

Reviewer comments:

Reviewer #1

(Remarks to the Author)
Summary
Menze et al. investigate the interrelated longitudinal changes in brain structure, cerebrovascular lesions, and cognition over four years in a cohort of 543 cognitively unimpaired older adults (the DELCODE study). Specifically, the authors employ latent growth curve modeling (LGCM) to examine trajectories of medial temporal lobe atrophy (measured as the MTL volume-to-ventricle ratio), white matter hyperintensity (WMH) lesion volume, and cognitive performance (using the PACC5 composite score). They also derive a “brain maintenance index” to quantify each individual’s overall preservation of brain structure and cognition. Key findings indicate that higher baseline WMH burden and more rapid WMH accumulation are associated with accelerated MTL atrophy over time. Both atrophy and WMH progression independently contribute to cognitive change. The study further reports that individuals with higher neuroticism, greater depressive symptoms, and lower cognitive engagement tend to have less favorable brain aging trajectories and a lower brain maintenance index.

Major Comments
1. I have some concerns about the novelty. Both age-related brain atrophy and WMH accumulation are well-established processes in normal aging, with numerous studies linking these changes to cognitive decline. Existing literature also suggests that these two processes are not independent: increased WMH burden is associated with greater cortical atrophy. Given this background, the novelty of the present study warrants closer scrutiny. Does the joint latent growth curve modeling of atrophy, WMH, and cognition truly reveal interactions or “couplings” that could not be detected in separate models or bivariate analyses? While the manuscript highlights the “brain maintenance index” as a novel outcome, it is essentially a predicted value from a multiple regression. It remains unclear how this adds substantial insight beyond the already known independent effects of WMHs and atrophy on cognition.
2. A notable finding is that the composite cognitive score (PACC5) improves slightly over time in this cohort. The LGCM reveals a positive cognitive slope, which the authors attribute to practice effects. This issue should be addressed more directly. An alternative approach would be to use cognitive measures that are more sensitive to age-related decline and less susceptible to practice effects—for example, individual episodic memory tasks, or composite scores specifically designed for longitudinal studies (potentially using alternate test forms to mitigate learning). The limitations of using PACC5 in this context should be discussed.
3. Related to the above, the impact of a positive cognitive slope on the LGCM requires careful consideration. The LGCM is presumably designed to estimate correlations between the slopes and intercepts of atrophy, WMH, and cognition. If cognitive performance unexpectedly improves over time, does this affect the estimation or interpretation of WMH and atrophy trajectories? The authors should provide evidence or sensitivity analyses to address whether the positive trend in cognition biases the model’s estimates of brain structure change.
4. The Methods state that outliers were removed before conducting LGCM analyses. Several aspects require clarification: How many outliers were removed, according to what criteria, and how might their exclusion influence the results? Blanket removal of outliers in longitudinal data can be problematic, as it risks excluding valid extreme cases and may bias the sample. Were these outliers individual timepoints, entire subjects, or specific variable values? The authors should report the number and type of data points removed. Additionally, have robust statistical approaches been considered instead of simply deleting outliers? This issue also applies to the multiple linear regression, where 38 outliers were removed. Using robust methods would align with best practices in statistical modeling.

5. The evaluation of the LGCM model fit indices raises some concern. The manuscript indicates p = 0.001 for the chi-square

test of model fit in SEM. This is unconventional; a non-significant chi-square ($p > 0.05$) is traditionally desired to indicate good model fit. The authors should give an interpretation.

6. The methods state that brain T1 measures were obtained using FreeSurfer, and WMH volumes were derived from an automated pipeline. However, there is no mention of manual editing or quality control of these automated segmentations. This omission is a significant methodological concern, as fully automated MRI segmentation is known to produce errors—FreeSurfer may mislabel tissue or include artifacts, especially in older adults with significant atrophy or lesions, and automated WMH segmentation can yield false positives. The authors should describe any steps taken for visual inspection or manual correction, or acknowledge this limitation if no such QC was performed.

Minor Comments
• Flowchart of Subject Selection: Including a CONSORT-style flow diagram or timeline showing participant inclusion and attrition would greatly enhance clarity.
• Supplementary Tables: The supplementary tables are not presented in the order referenced in the main text. Please revise for consistency and ease of reference.

Reviewer #2

(Remarks to the Author)
This study combines cross-sectional and longitudinal analyses to examine the co-evolution of ageing-related atrophy, white matter hyperintensities (WMH), and cognition, together with domain-specific and domain-general contributions of lifestyle and personality to brain-structure–related cognitive ageing. The key findings are:
1. WMH increased and MTLV-ratio declined over time, both indicating structural brain ageing.
2. These two brain measures were interrelated, with baseline WMH predicting greater subsequent atrophy.
3. PACC5 cognitive performance generally improved over time, most likely due to practice effects from repeated testing; however, this improvement was attenuated in individuals with more atrophy and faster WMH progression.
4. Personality and lifestyle factors — particularly neuroticism, depressive symptoms, and low cognitive engagement — were associated with poorer brain maintenance.

Specific comments:
(1) In Section 2.2.1, the authors state: "WMH volumes increased, MTLV-ratios decreased, and PACC5 performance improved throughout the study period." This pattern is unusual in ageing studies and is likely driven by practice effects, where repeated testing enhances performance and masks underlying decline. In contrast, Figure 1D appears to show a decrease in cognition (PACC5) over time. This apparent discrepancy should be clarified, ideally with a more detailed explanation of how the PACC5 trajectories were derived and interpreted in light of practice effects.
(2) The analysis of associations between neurocognitive trajectories and demographic variables (age, sex, years of education) is valuable. In the Education section, participants with more years of education had better baseline PACC5 scores but did not show greater longitudinal improvements in PACC5. I assume these refer to changes in PACC5 over the follow-up years? It would be helpful to clarify this point. Additionally, did you examine whether longitudinal MTLV-ratio changes were associated with education? This could add an interesting dimension to the results.
(3) Figure 2 is informative. As I understand it, individuals with faster WMH progression tend to have more negative cognitive slopes (red points lower on the z-axis), and those with steeper MTLV-ratio decline also tend to have more negative cognitive slopes. Individuals who perform better cognitively over time (blue points) generally have slower WMH progression and less atrophy. It may be worth producing an alternative, simplified 2D or clearer 3D plot to make this pattern more apparent to readers.
(4) For Figure 3, the current use of filled bars for positive correlations and striped bars for negative correlations could be replaced with a left–right bar format (e.g., bars to the right for positive, to the left for negative) to make the direction of correlations easier to interpret.
(5) The study includes plasma Aβ42/40 as a biomarker. Since other plasma biomarkers such as p-tau217, GFAP, and NfL are increasingly recognized for their value in detecting Alzheimer's disease pathology, could the authors clarify why only Aβ42/40 was included in the present analysis?
(6) Beyond total WMH volume, did you explore periventricular WMH (PVWMH) and deep WMH (DWMH) separately? These two WMH subtypes have distinct neuropathological underpinnings, risk factors, and cognitive associations, and their separate analysis might yield additional insights.

Reviewer #3

(Remarks to the Author)
Brain maintenance, also called brain reserve, has been of increasing interest in the research of cognitive aging and dementia prevention. Here, Menze et al. provided new insights on the issue by investigating how ageing-related atrophy (measured as medial temporal lobe to ventricle ratio [MTLV-ratio]) and white matter hyperintensities (WMH) co-evolved with each other and with cognitive ageing over four years. Based on a large sample of cognitively unpaired older adults, they found that steeper MTLV-ratio decline related to baseline WMH volume and its progression, and changes in MTLV-ratio and WMH volume independently contributed to cognitive changes. Besides, several lifestyle and personality factors were found to be related to unfavorable trajectories and brain maintenance index. The topic of this paper is of great interest to the field, but there were several issues in this manuscript that were difficult to follow at times and required some clarifications.

Major concerns:
1. the logic of Introduction part made me confused, brain maintenance should be highlighted at the very beginning of the manuscript, rather than ageing-related structural changes or cerebrovascular abnormalities. Furthermore, several statements should be modified to fit the purpose of the study, like Line 116-117, Line 135-136, current study neither aimed to elucidate the factors underlying the between-subject variability in contribution from structural and cerebrovascular brain changes to cognitive ageing, nor the nature of their interrelationships.
2. Towards the core statistical analysis, the latent growth curve modelling, did the authors have tried nonlinear modelling on latent slopes for the MTLV-ratio, WMH, and PACC5 performance? Besides, I would suggest that the authors conduct further moderation or mediation analysis between the three domains to get deeper information on their co-evolution relationship, rather than just simple correlation.
3. More explanations on operational definitions of brain-related domains are needed. the study focused on cognitively unimpaired older adults and the PACC5 was a general examination on cognitive function, why were only MTL regions chose to calculate the MTLV-ratio? How about the frontal and parietal regions? Similar issue existed for the WHM. although the authors discussed that 'our focus on global WMH precludes addressing potential regional associations with ageing-related atrophy', a inclusion of regions WMH would be able to establish a dedicated, more specific relationship between brain atrophy (MTLV-ratio or other) and WMH.

Minor concerns:
4. the potential practice effects of PACC5 performance (Line 198-200) could be separated from ageing-related changes by statistical modelling, referred to studies like Rabbitt et al., 2001 and Myrskylä et al., 2024.
5. please provide the distribution plots of the original data of WMH volumes, MTLV-ratio, and PACC5 performance.
6. How many participants have converted to MCI or dementia during the follow-ups? The number was not consistent in manuscript (n=93, Line 625) compared to the one in supplementary file (n = 69).
7. for the modifiable lifestyle factors and personality traits, I prefer not using the means of all available measurements as the manifestations of the lifestyle factors, since changes on those factors might related to MCI/dementia conversion or other health conditions.
8. typos? at Line 253, covstandaridzed = -0.181, where number in the supplementary table 3 was -0.281.

Reference
Rabbitt, P., Diggle, P., Smith, D., Holland, F., & Mc Innes, L. (2001). Identifying and separating the effects of practice and of cognitive ageing during a large longitudinal study of elderly community residents. Neuropsychologia, 39(5), 532-543.
Myrskylä, M., Hale, J. M., Schneider, D. C., & Mehta, N. K. (2024). Trends in Memory Function and Memory Impairment Among Older Adults in the United States and Europe, 1996–2018. The Journals of Gerontology, Series A: Biological Sciences and Medical Sciences, 79(Supplement_1), S11-S21.

Version 1:

Reviewer comments:

Reviewer #1

(Remarks to the Author)
The authors have responded thoroughly to my comments and have made substantial efforts to improve the manuscript. In particular, they conducted additional analyses and provided clearer explanations of their methodological choices. Regarding the concern about the PACC5 score and potential practice effects, the issue is not fully resolved, and the inherent limitations of the measure remain. However, the authors have acknowledged this limitation and provided a reasonable justification for retaining PACC5 as the cognitive outcome. They also implemented additional modelling strategies and expanded the discussion to address this concern more explicitly. I appreciate the authors' efforts to carefully consider the reviewer feedback and to strengthen the methodological transparency of the study. Importantly, the remaining limitations related to the PACC5 score do not appear to materially affect the main findings or the overall interpretation of the results. Overall, I believe the authors have responded constructively and the manuscript has been significantly improved as a result of the revision.

Reviewer #2

(Remarks to the Author)
The authors have carefully addressed the concerns raised in the previous round of review. The inclusion of new findings on plasma pTau181 and NfL is interesting. The revisions have improved the robustness of the manuscript, and I have no further major comments.

Reviewer #3

(Remarks to the Author)
the authors have conducted a great deal of supplementary analysis work and the revised and newly reported results have addressed my previous concerns. Good job.
after reading the remarkably revised manuscript, a new idea comes to my mind that although the joint modelling of brain structure and cognition gave a principled operationalization of brain maintenance, it also posed a big obstacle for other researchers or clinics to use the newly defined brain maintenance to evaluate the status of brain health, due to the need for sufficient data.

Open Access This Peer Review File is licensed under a Creative Commons Attribution 4.0 International License, which permits use, sharing, adaptation, distribution and reproduction in any medium or format, as long as you give appropriate credit to the original author(s) and the source, provide a link to the Creative Commons license, and indicate if changes were made.
In cases where reviewers are anonymous, credit should be given to 'Anonymous Referee' and the source.
The images or other third party material in this Peer Review File are included in the article's Creative Commons license, unless indicated otherwise in a credit line to the material. If material is not included in the article's Creative Commons license and your intended use is not permitted by statutory regulation or exceeds the permitted use, you will need to obtain permission directly from the copyright holder.
To view a copy of this license, visit https://creativecommons.org/licenses/by/4.0/

We sincerely thank the reviewers for their thoughtful and constructive feedback. Below, we provide detailed point-by-point responses, highlighting and pointing to the corresponding changes in our manuscript and supplementary materials to address the reviewers' comments.

In response to the reviewers' insightful suggestions, we have substantially revised and expanded the manuscript. The revision includes a complete restructuring of the Introduction to emphasize the brain maintenance framework more prominently, additional analyses and alternative model specifications addressing nonlinear trajectories, practice effects (manifest- vs. latent-level practice adjustment), robustness to outlier handling, robust regressions, regional models of WMH and cortical volumes, as well as complementary biomarker analyses including plasma pTau181 and plasma NfL. We reworked all figures and supplementary materials for clarity and consistency. Finally, we included a new validation analysis linking our brain maintenance index to an epigenetic ageing marker (DNA methylation clock) to externally corroborate its biological relevance.

These extensive revisions have considerably deepened the methodological transparency and strengthened the conceptual and empirical contribution of the study. We believe that these revisions have collectively improved the quality of our work. For convenience, links to response figures, response tables, or relevant pages are provided to facilitate cross-referencing within this document.

Reviewer #1 2

Reviewer #2 15

Reviewer #3 21

References 27

Reviewer #1

R1.0: Summary: Menze et al. investigate the interrelated longitudinal changes in brain structure, cerebrovascular lesions, and cognition over four years in a cohort of 543 cognitively unimpaired older adults (the DELCODE study). Specifically, the authors employ latent growth curve modelling (LGCM) to examine trajectories of medial temporal lobe atrophy (measured as the MTL volume-to-ventricle ratio), white matter hyperintensity (WMH) lesion volume, and cognitive performance (using the PACC5 composite score). They also derive a "brain maintenance index" to quantify each individual's overall preservation of brain structure and cognition. Key findings indicate that higher baseline WMH burden and more rapid WMH accumulation are associated with accelerated MTL atrophy over time. Both atrophy and WMH progression independently contribute to cognitive change. The study further reports that individuals with higher neuroticism, greater depressive symptoms, and lower cognitive engagement tend to have less favourable brain aging trajectories and a lower brain maintenance index.

Response: We thank the reviewer for their critical appraisal, their constructive feedback and the helpful suggestions. We have addressed these comments in the revised version of our work and will provide detailed responses to each of them below. We believe that – in accordance with the feedback of the other reviewers – they enhance the impact and transparency of our manuscript. In the revised version of the manuscript, we have revised all sections, included additional statements, clarifications and several new analyses.

R1.1: I have some concerns about the novelty. Both age-related brain atrophy and WMH accumulation are well-established processes in normal aging, with numerous studies linking these changes to cognitive decline. Existing literature also suggests that these two processes are not independent: increased WMH burden is associated with greater cortical atrophy. Given this background, the novelty of the present study warrants closer scrutiny. Does the joint latent growth curve modelling of atrophy, WMH, and cognition truly reveal interactions or "couplings" that could not be detected in separate models or bivariate analyses? While the manuscript highlights the "brain maintenance index" as a novel outcome, it is essentially a predicted value from a multiple regression. It remains unclear how this adds substantial insight beyond the already known independent effects of WMHs and atrophy on cognition.

Response: We thank the reviewer for this critical appraisal. We agree that the pairwise links between WMH, atrophy, and cognition are established in the literature. We nevertheless argue that our work provides methodological, conceptual, and empirical novelty (outlined below), which, as the reviewer's comment suggests, we did not articulate clearly enough in the initial version of the manuscript. In response, we have revised the manuscript to clarify and strengthen our contributions to the literature.

1. Methodological Novelty:

One of our primary aims was to characterise brain maintenance – the shared variability between changes in cognition and brain integrity – which, to our knowledge, has not yet been formally operationalised in the literature. We achieved this by modelling the co-evolution of MTLV-ratio, WMH, and PACC5 over time using the trivariate LGCM and explicitly isolating the component of cognitive ageing attributable to structural preservation.

Methodologically, a long tradition in SEM has demonstrated both for SEM in general (e.g. [1]) and LGM in particular (e.g. [2]) that the integration and estimation of the full causal hypothesis or theory into a single model is desirable and beneficial for reasons both methodological (e.g. appropriate standard errors, violations of measurement invariance [3] and more) and substantive (e.g. integration of measurement and theory). Although approaches exist to deal with estimation or convergence problems that arise when overly complex models are fit to modestly sized datasets, we demonstrate in a uniquely large dataset that we can, in fact, achieve the 'gold standard' of joint estimation of the entire theoretical model.

This aim cannot be achieved using three separate univariate or bivariate LGCMs, as this would omit a key statistical requirement of Stern's brain maintenance framework[4]: demonstrating that all studied processes evolve as a coupled system, evidenced by an adequate fit of their joint covariance structure to the longitudinal data. Separate models implicitly assume independence or fail to assess the global fit of the coupling, rendering any post-hoc combination of slopes less rigorous. In contrast, the good fit of our trivariate LGCM provides an omnibus validation of the coupled system (CFI = 0.998; RMSEA = 0.022 [0.00, 0.038]; SRMR

= 0.018), alongside the meaningful effect size of the key associations provides compelling evidence of the 'coupled system' that could not be achieved in a principled and rigorous manner with either non-growth curve approaches or sequential bivariate models.

Our methodological contribution lies in the parsimony and coherence of a full multivariate SEM, in which all domains are jointly estimated within a trivariate LGCM. This model estimates all parameters, better separates measurement error from the growth process of interest, incorporates covariate effects, and cross-domain covariances only once, aligning with Stern's framework and yielding a single, globally consistent covariance structure with harmonised scaling and missing-data handling for maintenance analyses.

2. Conceptual Novelty
The reviewer notes that our maintenance index is "a predicted value from a multiple regression". We would like to clarify that, while methodologically straightforward after a complex longitudinal model of multi-domain changes is established, our index represents a theoretically grounded operationalisation of brain maintenance as recently defined by Stern et al.[4] and thus constitutes a novel contribution. A related model-based cross-sectional single score characterization of age-related brain differences has been applied extensively in the field of ageing research to quantify individual's 'brain age' (e.g. [5]). There is evidence that the cross-sectional brain age gap might be a measure of resilience with indications for construct and predictive validity (e.g. [6,7.]). Although the latter approach to ageing characterisation is very popular in the field, there is also evidence that it might be limited by its cross-sectional nature[8]. This suggests the importance of alternative longitudinal characterization of ageing as proposed in our work. Moreover, prior work has largely focused on cognitive reserve, typically defined as residual performance exceeding that expected given brain structure. In contrast, brain maintenance targets shared variability – the extent to which cognition is preserved because brain integrity is preserved. By deriving a predicted cognitive slope from joint structural slopes, we isolate the component of cognitive ageing attributable to structural preservation, which, to our knowledge, has not been done previously. This yields a subject-specific metric that fulfils the theoretical requirement to link longitudinal structural brain preservation to longitudinal functional/cognitive preservation. More complex extensions (e.g., additional domains, non-linear models) could build on this framework in future work.

3. Empirical Novelty
Beyond the modelling framework, we would like to emphasize that our study contributes two novel empirical insights that extend the current literature in the following ways:

Identifying Modifiable Drivers of Maintenance: While many studies link lifestyle to cognition or brain structure separately, we extend this by identifying contributors to the maintenance process itself (the brain-structure-related cognitive slope). We show that *modifiable* factors – specifically depressive symptoms, neuroticism, and openness – are not just correlates of cognitive decline but are significantly associated with the proposed brain maintenance index. This follows the proposal by Stern et al. (2023, p.102-103) and moves the field beyond describing that maintenance happens, to identifying who maintains function via these specific contributors and pathways. Accordingly, we now added differences in cognitively stable and converting individuals in the brain maintenance index (**Supplementary Figure 4H**).

Biological Validation (DunedinPACE): Finally, in response to the reviewer's request, we conducted a new analysis and provide first evidence that our proposed brain maintenance index captures a real biological phenomenon and not just statistical noise. Specifically, we added a new validation analysis using DunedinPACE, a DNA-methylation marker of ageing pace[9] (predicted using epigenetic information). We observed that individuals with higher brain maintenance scores exhibited a significantly slower pace of systemic biological aging in our cohort. This connects the macro-structural/cognitive model to molecular ageing mechanisms, providing external validity to our trivariate maintenance approach.

To reflect these points, we have revised the Introduction, Methods, and Discussion sections as follows:

Introduction (revised text last paragraph):
"To our knowledge, the third requirement of the brain maintenance framework—isolating the component of cognitive ageing attributable specifically to structural preservation[9]—has not yet been explicitly addressed. We therefore set out to achieve three complementary objectives. First, we modelled coupled longitudinal changes across cerebrovascular pathology, brain atrophy, and cognitive performance within a unified multivariate latent growth curve modelling (LGCM) framework. We thus extend prior work on pairwise

associations by a parsimonious model able to validate global fit of the coupled system. Second, we derived individual cognitive trajectories as a function of joint structural change, enabling explicit quantification of the proportion of cognitive ageing explained by preserved brain integrity. This provides a principled operationalisation of brain maintenance as a longitudinal, multi-domain, system-level construct. Third, we identified modifiable lifestyle factors and personality traits that contribute to inter-individual differences in baseline levels and longitudinal change across neurocognitive domains and our proposed domain-general brain maintenance index. We demonstrate the utility of this framework in a large cohort of cognitively unimpaired older adults followed annually over four years, yielding novel mechanistic insight into the neurobiological basis of brain maintenance and identifying potential targets for preventive strategies against cognitive decline."

Methods (revised in 4.5.2 Latent growth curve modelling):
"We employed a trivariate LGCM to obtain a parsimonious, internally coherent estimation of measurement parameters, growth factors, and covariate effects within one joint likelihood. This unified estimation avoids multiple-comparison inflation, prevents redundant parameter estimation, ensures appropriate standard errors and is essential for identifying a latent brain maintenance index based on simultaneously estimated brain-related slopes."

Methods (added to 4.5.3 Domain-specific and domain-general contributors to neurocognitive changes):
"To validate and assess the biological relevance of the brain maintenance index, we assessed whether it related to an independent molecular marker of biological ageing pace. More specifically, we correlated it with the baseline DunedinPACE[93] epigenetic-ageing score derived from DNA-methylation profiling available in a subsample of participants (n = 502).
DNA methylation was assessed in blood using the Illumina MethylationEPIC BeadChip, and arrays were scanned using the Illumina iScan system. The methylation data were processed using the R package meffil (v1.3.8)[94]. Quality control followed the default settings implemented in the package. Functional normalization was applied using 20 principal components, with batch information included as a fixed effect The normalized beta values were subsequently used to calculate DunedinPACE using the R package dnaMethyAge (v0.2.0). Partial Spearman's correlations were computed between the maintenance index and DunedinPACE, adjusting for age, sex, years of education, and TICV."

Results (added to 2.3 Modifiable lifestyle factors and personality traits associate to changes in neurocognitive domains and brain maintenance):
"Importantly, lower DunedinPACE, indicating slower biological ageing, was significantly associated with higher brain maintenance index ($\rho = -0.144$, $p_{FDR} = 0.007$; Figure 4C). Individuals who remained cognitively stable over time exhibited higher brain maintenance than those who subsequently converted (Supplementary Figure 4H)."

Discussion (Revised/Added text):
"Moreover, we provide evidence for and clarify mechanisms of brain maintenance, demonstrating that changes in MTLV-ratio and total WMH were linked to and exerted additive, independent effects on cognitive performance change[9,24]. While bivariate approaches might obscure this distinction, potentially attributing cognitive decline to a single marker when it is actually driven by both, the present approach highlights the coupled evolution of brain measures, and suggests that cerebrovascular pathology compromises cognition not only by accelerating neurodegeneration but also via distinct, direct pathways."

and

"Importantly, we provide novel evidence for the validity and relevance of our proposed brain maintenance index by identifying specific predictors that explain interindividual heterogeneity, and illustrating its higher expression in cognitively stable compared to converting individuals (Supplementary Figure 4H). Additionally, we demonstrated its association with DunedinPACE, suggesting that individuals who exhibit slower systemic molecular ageing also show preserved neurocognitive ageing trajectories, providing external validation of the brain maintenance index."

R1.2: A notable finding is that the composite cognitive score (PACC5) improves slightly over time in this cohort. The LGCM reveals a positive cognitive slope, which the authors attribute to practice effects. This issue should be addressed more directly. An alternative approach would be to use cognitive measures that are more sensitive to age-related decline and less susceptible to practice effects—for example, individual

episodic memory tasks, or composite scores specifically designed for longitudinal studies (potentially using alternate test forms to mitigate learning). The limitations of using PACC5 in this context should be discussed.

Response: We thank the reviewer for this insightful comment. While practice effects themselves were shown to be diagnostically informative[10], disentangling them from ageing-related change could clarify how different neurocognitive mechanisms contribute to observed performance trajectories. To clarify our findings, 59.12% of participants showed positive changes in PACC5, while the remainder exhibited declines. This suggests, as all reviewers note (see **R2.1** and **R3.4**), that many participants may exhibit retest-related practice effects – an aspect that warrants further investigation

We agree that distinguishing retest-related practice effects from ageing-related cognitive change might be a crucial issue, particularly since only some PACC5 subtests were administered in parallel forms (SDMT, FCSRT), while others were not (MMSE, Logical Memory, fluency). Although alternate forms may reduce retest effects, they do not eliminate them. We therefore opted for a model-based correction rather than restricting analyses to a single subtest. Following Ferrer et al.[11] and Hoffman et al.[12], we implemented two complementary approaches:

1. **Manifest-level practice effects**: practice modelled as a fixed population-level offset at all follow-ups (**Response Figure 1A**).
2. **Latent-level individual practice effects**: PACC5 scores loaded on a latent practice factor (0 = baseline; 1 = all follow-ups). Although initially allowing interindividual variance in this factor led to non-convergence and negative variance, constraining its variance to zero yielded a concise and stable model (**Response Figure 1B**).

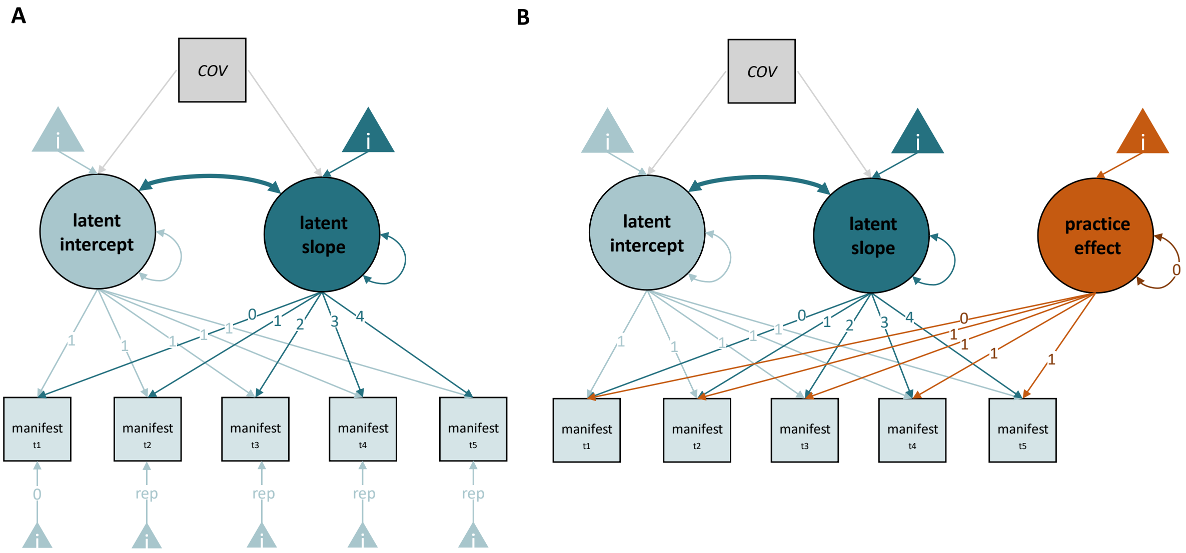

Response Figure 1. Schematic depiction of implementation of practice effects in cognitive retesting explored here. (A) Modelling a constant offset on manifest-level. Practice is modelled directly at the manifest level via a fixed offset ("rep" for repetition) applied to retest occasions. **(B)** Modelling of a latent-level practice effect. Here an offset after the first measurement is created. Therefore, loading at baseline (t1, first measurement occasion) is constrained to 0, while loading is fixed to 1 for subsequent measurement occasions. Its intercept (triangle) describes the mean population-level constant offset of practice effect. Although variance of the latent practice effect could, in principle, be estimated freely, it here was constrained to 0 for model stability (see text).

Both scenarios imply a robust, population-level practice effect, but we found no evidence for substantial between-person variability in that gain in our cohort. Consequently, the latent slope of cognitive performance represents net change in cognitive performance free from population-level practice effect and fit the data well (

Response Table 1).

Response Table 1. Model fit parameters for the model without accounting for practice effects and the models accounting for practice effects on manifest- and latent-level.

Model	Scaled Chi² (df)	Robust CFI	Robust RMSEA [90% CI]	SRMR
Without practice effect	35.22 (29)	1.00	0.008 [0.00, 0.04]	0.021
Manifest-level practice effect	28.22 (28)	1.00	0.00 [0.00, 0.04]	0.020
Latent-level practice effect (variance = 0)	28.22 (28)	1.00	0.00 [0.00, 0.04]	0.020

Moreover, by fixing the latent practice effect's variance to zero, both modelling approaches became numerically identical (**Response Table 2**). In the revised manuscript, we therefore adopted the manifest-level practice offset as the most parsimonious and stable solution. This specification effectively adjusts for population-level practice effects while preserving the interpretability of the latent cognitive slope as reflecting ageing-related change. Importantly, inclusion of this offset reduced the mean level change in the latent cognitive slope but did not reduce its variance, indicating that practice-related gain was primarily a population-level effect. This suggests that obtained slopes are an unbiased reflection of individual differences of change. We also emphasise, that this works' focus is not mean-level PACC5 change but interindividual differences and covariances between latent changes across the three neurocognitive domains. The adjustment thus accounted for the population-level retest-related offset while maintaining interindividual variability in longitudinal change (**Response Table 2**). This was essential for our study goal of examining individual differences in ageing slopes rather than mean trajectories[13]. The stable variance of the latent cognitive slope also preserved cross-domain associations, confirming that the adjustment successfully captured group-level practice effects without altering the interpretation or conclusions of our results.

Response Table 2. Parameter estimates for the linear cognitive slope, its variance, and mean practice effect for a univariate cognition model without accounting for practice effects and the models accounting for practice effects on manifest- and latent-level.

Model	Practice mean *est(SE), p*	cognitive slope *est(SE), p*	Variance cognitive slope *est(SE), p*
Without practice effect	/	0.038 (0.009), <0.001	0.010 (0.003), 0.001
Manifest-level practice effect	0.076 (0.029), 0.008	0.018 (0.012), 0.127	0.010 (0.003), 0.001
Latent-level practice effect (variance = 0)	0.076 (0.029), 0.008	0.018 (0.012), 0.127	0.010 (0.003), 0.001

We report this approach in our methods (4.5.2):

"Given the annual cognitive assessment, we additionally accounted for retest-related practice effects in the cognitive domain. We considered two conceptually equivalent approaches by comparing modelling of manifest-level and latent-level practice effect[86,87]. Although latent-level modelling would theoretically allow estimation of interindividual variability in practice effects, no such variability was observed here. Therefore, to adjust for population-level practice effects parsimoniously, practice was modelled as a fixed population-level offset at all manifest follow-ups."

Finally, we note that our modelling approach did not capture person-specific practice effects, as these were not supported by our data. In more diverse samples, such effects may nonetheless vary between individuals and warrant further investigation, as they could carry important information. We now clarify this point in the revised discussion:

"Second, although we accounted for a population-level practice effect in PACC5 performance, interindividual practice effects could not be reliably estimated, but they may vary in more diverse samples. Future studies are encouraged to explore both ageing and practice. They could inform about meaningful

differences in ageing- vs. retest-related effects learning that could relate to other neurocognitive ageing processes or lifestyle factors[69–71].”
Consistent with previous studies indicating that composite scores are more sensitive to ageing-related and preclinical amyloid-associated cognitive change than single-test measures due to lower intraindividual variability[14–16], we retained the PACC5 composite. We would also argue that the PACC5 composite offers higher reliability, broader cognitive coverage, and facilitates comparability with previous longitudinal and clinical studies in the same cohort. Its widespread use also facilitates comparability across studies. The rationale is now emphasised in the revised Methods (4.5.3):
“We assessed cognitive performance annually using the established preclinical Alzheimer’s cognitive composite score (PACC5)[32], a measure for early cognitive change specific for AD. PACC5 was chosen to enhance comparability across studies and since composite scores have been shown to be more sensitive to ageing-related cognitive change than individual test measures[82,83].”

In line with the reviewer’s feedback, we further expanded the discussion to acknowledge alternative composite measures:
“Fifth, while our analysis was based on a specific operationalization of the three neurocognitive domains, future research may benefit from further examining lesion load within specific hubs of cognitive networks[46], clarify the differential relevance of various CSVD markers[16,17,35,42], integrating more recently established blood biomarkers alongside biological staging of AD[72], and expand the cognitive domains studied. For instance, composite scores targeting executive functions may be additionally informative in the contexts of healthy ageing or vascular cognitive impairment[73,74].”

R1.3: Related to the above, the impact of a positive cognitive slope on the LGCM requires careful consideration. The LGCM is presumably designed to estimate correlations between the slopes and intercepts of atrophy, WMH, and cognition. If cognitive performance unexpectedly improves over time, does this affect the estimation or interpretation of WMH and atrophy trajectories? The authors should provide evidence or sensitivity analyses to address whether the positive trend in cognition biases the model’s estimates of brain structure change.

Response: We thank the reviewer for bringing up this issue. In our trivariate LGCM, each domain (WMH, atrophy, cognition) is parametrized by its independent latent intercept and slope (η_I^k, η_S^k), k=1,2,3 and domain-specific measurement model. Cross-domain relations are represented via freely estimated covariances among these latent factors e.g., Cov(η_S^{WMH}, η_S^{COG}), not via regressions. Under this specification, the joint likelihood factorizes such that the means and (co)variances of WMH/atrophy growth factors are identified by their own submodels; the sign or magnitude of the cognitive slope does not enter the estimating equations for WMH or atrophy growth parameters. Consequently, a positive cognitive slope does not bias the estimation of WMH or atrophy intercepts/slopes.

As suggested by the reviewer we addressed this concern also empirically by fitting individual models per domain. We note that within these reported individual models, we also accounted for a comment by reviewer #3 (**R3.2**) concerning possible nonlinear dynamics. Our approach here was two-fold: First, we fitted separate models per each neurocognitive domain of interest to examine the trends in mean slopes. Second, examining whether nonlinear slopes, i.e. quadratic growth, improved model fit.

Trends in trajectories and their variance remained similar in estimating univariate LGCM separately for each domain. Hence, empirically simultaneous estimation and covariance between them did not bias slope estimates in either domain significantly. However, the conceptual and statistical benefits of a simultaneous multi-domain SEM model of neurocognitive changes – the goal of this modelling study – were discussed in an above response. In order to address the reviewers’, concern we provide details of each individual model in **Response Figure 2**. In accordance with reviewer #3’s suggestion of nonlinear slopes, we here also note that we tested possible nonlinear dynamics in each domain separately and found that a quadratic slope improved model fit for MTLV-ratio (**Response Figure 2C&D**). For the full detailed information, we kindly refer to the respective comment and our response in **R3.2**. Consequently, we now provide their details in our methods (4.5.2) and the supplements of our overhauled manuscript, detailing this two-step approach of first characterizing (potentially non-linear) individual change processes per domain before integrating them in a parsimonious trivariate model, which is now updated to accommodate quadratic change in MTLV-ratio.

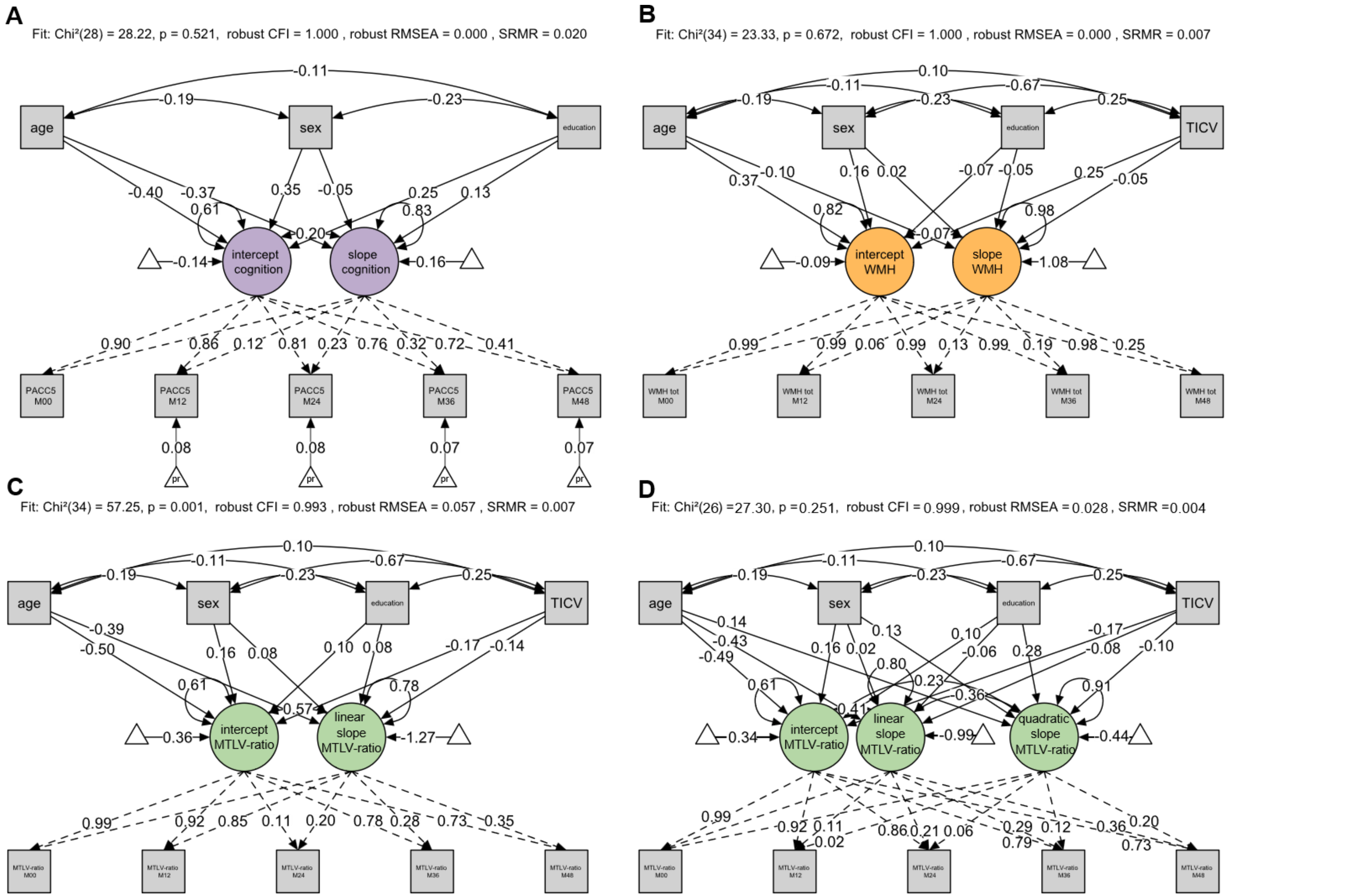

Response Figure 2. Separate Latent Growth Curve models per neurocognitive domain. Model fit indices are provided above each model. Each model included covariates age, sex, years of education, and – in case of WMH and MTLV-ratio – TICV. Circles represent latent variables, triangles intercepts of latent variables, double-headed arrows variance in latent intercepts and slopes. Standardized factor loadings are shown. Dashed lines indicate fixed loadings (intercepts = c(1,1,1,1,1); linear slopes = c(0,1,2,3,4); quadratic slope = c(0,1,4,9,16)). (**A**) PACC5 performance. A model with linear slope only was parsimonious enough to represent data. Triangles below PACC5 manifest variables indicate manifest practice-level offset (pr = practice). (**B**) Total WMH volumes. A model with linear slope only was parsimonious enough to represent data. (**C**) MTLV-ratio with linear slope only. (**D**) MTLV-ratio with linear and quadratic slope. Adding a quadratic slope improved model fit significantly over a simpler linear slope only model for MTLV-ratio ($\chi 2\Delta = 27.503$, $\Delta df = 8$, $p < 0.001$).

R1.4: The Methods state that outliers were removed before conducting LGCM analyses. Several aspects require clarification: How many outliers were removed, according to what criteria, and how might their exclusion influence the results? Blanket removal of outliers in longitudinal data can be problematic, as it risks excluding valid extreme cases and may bias the sample. Were these outliers individual time points, entire subjects, or specific variable values? The authors should report the number and type of data points removed. Additionally, have robust statistical approaches been considered instead of simply deleting outliers? This issue also applies to the multiple linear regression, where 38 outliers were removed. Using robust methods would align with best practices in statistical modelling.

Response: We thank the reviewer for this critical appraisal. Our previous manuscript version was not communicating clearly the applied methodological procedures regarding outlier detection and removal. To minimize inference biases and violations of modelling assumptions, a careful outlier detection and removal are generally suggested in large-scale neuroimaging studies that can contain various image or processing artefacts[17]. However, techniques and recommendations still vary across imaging protocols and domains, and we opted for a consistent solution across all three outcome domains explored in our study. We overhauled the respective section as follows (see revised methods section 4.5.2):

"Prior to model fitting, we identified and removed outliers for WMH, brain morphometric measures, and PACC5 performance, separately for each assessment time point. Values exceeding the interquartile range by more than Q3 + 1.5×IQR or below Q1 - 1.5×IQR of the median were considered outliers and set to missing in the respective time point. The IQR-based criterion follows distribution-free outlier detection[88], which has been shown to provide a robust compromise between sensitivity and false-positive control across a wide range of non-Gaussian distributions[89,90]. The number of detected outliers per variable and time point for each model is reported in the corresponding supplementary tables detailing model specifications."

Hence, outliers were detected based on interquartile range within each time point and per domain separately. Detected values were set to NA, in order to avoid losing entire subjects. With this procedure we allowed for missing values in the model. Since we were using FIML during model estimation, these missing values were addressed. Additionally, all our models were estimated using a robust estimator (MLR). We acknowledge that identifying and removing outliers can be problematic, especially in longitudinal data. Hence, we reran the global trivariate model on the full data including outlier values. We found the model, including a quadratic term for MTLV-ratio failed to converge, producing negative variance in latent quadratic slope estimate, likely due to (over)fitting on extreme values.

We tested a model restricting the quadratic slope variance estimate to a small positive number (0.0001), resulting in model convergence. Model fit was overall adequate ($\chi^2(136) = 235.70$, $p < 0.001$; $CFI = 0.994$; $RMSEA = 0.039$ [0.022, 0.054]; $SRMR = 0.016$; *Yuan-Bentler scaling factor* = 1.197; see **Response Table 3**), though slightly worse than the model based on cleaned data ($\chi^2(135) = 155.06$, $p = 0.114$; $CFI = 0.998$; $RMSEA = 0.022$ [0.00, 0.038]; $SRMR = 0.018$; *Yuan-Bentler scaling factor* = 1.081). Even, when imposing the same slope constraint to the model based on clean data, model fit of the latter was slightly better ($\chi^2(136) = 154.90$, $p = 0.128$; $CFI = 0.998$; $RMSEA = 0.021$ [0.00, 0.038]; $SRMR = 0.018$; *Yuan-Bentler scaling factor* = 1.084). Generally, we still observed an increase in WMH volumes and a decrease in MTLV-ratio over follow ups (intercept of WMH slope: $B = 1.065$, $p < 0.001$; intercept of linear MTVL-ratio slope: $B = -0.787$, $p < 0.001$), while PACC5 change was not significant ($B = 0.038$, $p = 0.687$).

With regards to associations between latent variables, we observed similar results: Individuals with higher baseline WMH volumes had lower baseline MTLV-ratios (*intercept WMH ~ intercept MTLV-ratio*: $cov_{Standardized} = -0.117$, $p = 0.004$) and experienced steeper declines in MTLV-ratios over time (*intercept WMH ~ slope MTLV-ratio*: $cov_{Standardized} = -0.129$, $p = 0.023$). Individuals with better initial PACC5 performance showed higher baseline MTLV-ratios (*intercept cognition ~ intercept MTLV-ratio*: $cov_{Standardized} = 0.179$, $p < 0.001$) and less MTLV-ratio decline (*intercept cognition ~ slope MTLV-ratio*: $cov_{Standardized} = 0.250$, $p = 0.005$), the latter implying an ameliorating effect of cognitive capability on ageing-related atrophy. PACC5 performance changes were more pronounced in individuals with higher MTLV-ratios at baseline, and with slower declines in MTLV-ratios (*slope cognition ~ intercept MTLV-ratio*: $cov_{Standardized} = 0.384$, $p < 0.001$; *slope cognition ~ slope MTLV-ratio*: $cov_{Standardized} = 0.421$, $p = 0.002$). The association between lower progression of WMH and higher PACC5 performance changes yielded a trend (*slope cognition ~ slope WMH*: $cov_{Standardized} = -0.173$, $p = 0.085$).

Overall, both models – based on data with and without outlier exclusion – allowed for consistent conclusions and interpretations. Moreover, handling outliers before model fitting facilitated model estimation without convergence issues. We therefore believe, that our outlier detection procedure followed a robust analytical strategy, and we report this in our manuscript.

We also appreciate the reviewer’s feedback on using robust multiple regression models, which we implemented using the package “robustbase” that employs a robust MM-type regression estimator, resistant to outliers. We adapted our modelling procedures accordingly and report the new results in the revised manuscript. Overall, results did not alter and our interpretation of additive effects of MTLV-ratio decline and WMH progression on cognitive change remained the same.

Response Table 3. Model details of the main trivariate latent growth curve model between PACC5, total WMH volumes, and MTLV-ratio without outlier correction. Models included covariates age, sex, years of education, and total intracranial volume (TICV). Model converged after constraining the quadratic slope variance to a small positive number. Model fit: $\chi^2(136) = 235.70$, $p < 0.001$; CFI = 0.994; RMSEA = 0.039 [0.022, 0.054]; SRMR = 0.016; Yuan-Bentler scaling factor = 1.197.

				Estimate	SE	Std. Estimate	Z	p
Association with Demographics	Intercept cognition	~	Age	-0.323	0.034	-0.386	-9.589	0.000
		~	Sex	0.295	0.032	0.353	9.165	0.000
		~	Years of Education	0.227	0.035	0.271	6.518	0.000
	Slope cognition	~	Age	-0.051	0.010	-0.383	-5.020	0.000
		~	Sex	-0.007	0.009	-0.054	-0.820	0.412
		~	Years of Education	0.008	0.010	0.058	0.806	0.420
	Intercept WMH	~	Age	0.370	0.040	0.374	9.199	0.000
		~	Sex	0.151	0.055	0.152	2.733	0.006
		~	Years of Education	-0.071	0.040	-0.072	-1.797	0.072
		~	TICV	0.249	0.052	0.251	4.812	0.000
	Slope WMH	~	Age	-0.008	0.005	-0.125	-1.643	0.100
		~	Sex	0.004	0.006	0.065	0.684	0.494
		~	Years of Education	-0.003	0.004	-0.053	-0.762	0.446
		~	TICV	-0.002	0.005	-0.031	-0.394	0.693
	Intercept brain	~	Age	-0.442	0.035	-0.498	-12.677	0.000
		~	Sex	0.119	0.037	0.134	3.253	0.001
		~	Years of Education	0.085	0.031	0.096	2.737	0.006
		~	TICV	-0.185	0.035	-0.209	-5.226	0.000
	Linear slope brain	~	Age	-0.042	0.007	-0.344	-6.008	0.000
		~	Sex	0.005	0.008	0.042	0.669	0.504
		~	Years of Education	0.002	0.007	0.013	0.217	0.828
		~	TICV	-0.016	0.008	-0.132	-1.918	0.055
	Quadratic slope brain	~	Age	0.001	0.001	0.124	0.874	0.382
		~	Sex	0.001	0.002	0.092	0.538	0.590
		~	Years of Education	0.002	0.001	0.198	1.433	0.152
		~	TICV	-0.001	0.002	-0.079	-0.522	0.602
Mean baseline level and change	Intercept cognition	~	1	-0.103	0.033	-0.123	-3.151	0.002
	Slope cognition	~	1	0.005	0.012	0.038	0.403	0.687
	Intercept WMH	~	1	-0.098	0.039	-0.099	-2.513	0.012
	Slope WMH	~	1	0.068	0.004	1.065	15.888	0.000
	Intercept brain	~	1	0.165	0.030	0.186	5.544	0.000
	Linear slope brain	~	1	-0.097	0.007	-0.787	14.096	0.000
	Quadratic slope brain	~	1	-0.005	0.001	-0.490	-3.628	0.000

				Estimate	SE	Std. Estimate	Z	p
**Variances latent variables**	Intercept cognition	~~	Intercept cognition	0.434	0.037	0.620	11.662	0.000
	Slope cognition	~~	Slope cognition	0.015	0.004	0.849	3.723	0.000
	Intercept WMH	~~	Intercept WMH	0.804	0.052	0.820	15.335	0.000
	Slope WMH	~~	Slope WMH	0.004	0.001	0.969	3.946	0.000
	Intercept brain	~~	Intercept brain	0.475	0.051	0.602	9.357	0.000
	Linear slope brain	~~	Linear slope brain	0.013	0.004	0.840	3.006	0.003
	Quadratic slope brain	~~	Quadratic slope brain	0.000	.	0.949	.	.
**Association between latent variables**	Intercept cognition	~~	Slope cognition	0.021	0.009	0.256	2.332	0.020
		~~	Intercept WMH	-0.031	0.025	-0.053	-1.276	0.202
		~~	Slope WMH	0.001	0.003	0.017	0.229	0.819
		~~	Intercept brain	0.081	0.023	0.179	3.482	0.000
		~~	Linear slope brain	0.019	0.007	0.250	2.826	0.005
		~~	Quadratic slope brain	-0.001	0.001	-0.164	-0.815	0.415
	Slope cognition	~~	Intercept WMH	-0.008	0.008	-0.069	-0.966	0.334
		~~	Slope WMH	-0.001	0.001	-0.173	-1.721	0.085
		~~	Intercept brain	0.032	0.009	0.384	3.700	0.000
		~~	Linear slope brain	0.006	0.002	0.421	3.038	0.002
		~~	Quadratic slope brain	0.000	0.000	0.213	0.832	0.405
	Intercept WMH	~~	Slope WMH	-0.005	0.006	-0.087	-0.860	0.390
		~~	Intercept brain	-0.073	0.025	-0.117	-2.852	0.004
		~~	Linear slope brain	-0.013	0.006	-0.129	-2.279	0.023
		~~	Quadratic slope brain	0.001	0.001	0.057	0.415	0.678
	Slope WMH	~~	Intercept brain	-0.004	0.003	-0.102	-1.532	0.126
		~~	Linear slope brain	0.000	0.001	-0.049	-0.614	0.539
		~~	Quadratic slope brain	0.000	0.000	-0.270	-1.484	0.138
	Intercept brain	~~	Linear slope brain	0.036	0.007	0.462	4.895	0.000
		~~	Quadratic slope brain	0.001	0.001	0.095	0.532	0.595
	Linear slope brain	~~	Quadratic slope brain	-0.001	0.000	-0.633	-1.720	0.085
**Association covariates**	Age	~~	Sex	-0.194	0.042	-0.194	-4.662	0.000
		~~	TICV	0.103	0.041	0.103	2.497	0.013
		~~	Years of Education	-0.111	0.046	-0.111	-2.430	0.015
	Sex	~~	TICV	-0.665	0.020	-0.665	-33.180	0.000
		~~	Years of Education	-0.227	0.039	-0.227	-5.791	0.000
	Years of Education	~~	TICV	0.251	0.039	0.251	6.514	0.000

R1.5: The evaluation of the LGCM model fit indices raises some concern. The manuscript indicates p = 0.001 for the chi-square test of model fit in SEM. This is unconventional; a non-significant chi-square (p > 0.05) is traditionally desired to indicate good model fit. The authors should give an interpretation.

Response: We thank the reviewer for raising this nuanced concern. Indeed, $\chi 2$-square tests in the context of SEM modelling are desired to be non-significant. At the same time, they have been reported to be e.g. sensitive to large sample sizes or model complexity among other shortcomings[18]. Therefore, it is recommended to assess model fit by a variety of model fit indices, usually including CFI, RMSEA, SRMR, and frequently TLI. We acknowledge that our reported $\chi 2$-square indicates a significant deviation from perfect fit, noting that given our large sample, even small deviations from perfect fit would be statistically significant. Therefore, we report a range of absolute and relative fit indices to provide a more holistic assessment in model fit alongside the $\chi 2$-square. Across the boards, these suggest that our chosen models fit the data quite closely.

R1.6: The methods state that brain T1 measures were obtained using FreeSurfer, and WMH volumes were derived from an automated pipeline. However, there is no mention of manual editing or quality control of these automated segmentations. This omission is a significant methodological concern, as fully automated MRI segmentation is known to produce errors – FreeSurfer may mislabel tissue or include artefacts, especially in older adults with significant atrophy or lesions, and automated WMH segmentation can yield false positives. The authors should describe any steps taken for visual inspection or manual correction, or acknowledge this limitation if no such QC was performed.

Response: We thank the reviewer for important methodological issue. We apologize for the lack of this detail in the submitted version of the methods. We addressed the issue in our revised manuscript as follows:

We included more information about the quality control procedures for both FreeSurfer as well as WMH segmentation pipelines in in the respective method's section. More specifically, we added:
"We used FreeSurfer's longitudinal pipeline for segmentation of T1-weighted MPRAGE images. Automatically generated segmentation and parcellation maps were overlaid on the corresponding MPRAGE images and visually inspected by trained raters to identify and discard gross errors or abnormalities (e.g., topological defects, mislabelled cortical regions, or failed skull stripping)."

and

"We used WMH of all participants and time points as a proxy for cerebrovascular abnormalities. The WMH quantification procedure was as follows. First, we co-registered T1w and T2w FLAIR images for each individual at each session. Second, we processed the co-registered images with the LST-AI toolbox[76]. Third, we post-processed the resulting segmentations to reduce false positive segmentation of WMH. Post-processing also involved excluding lesions that were located outside the supratentorial white matter, specifically those identified in cortical and subcortical grey matter, the brainstem, the choroid plexus, or the septum pellucidum."

This visual inspection procedure was applied consistently across sites and time points to ensure reproducibility and to detect only gross segmentation failures, without modifying segmentations directly. In total, n = 3208 scans across all DELCODE participants and longitudinal sessions were processed. Given the multicentre, longitudinal design and sample size, full manual correction for each scan was not practically feasible. Instead, we implemented visual QC as a cost-efficient and established compromise between feasibility and accuracy, an approach consistent with best-practice recommendations in large-scale neuroimaging studies such as ADNI, UK Biobank, and the Human Connectome Project, where automated segmentation combined with systematic QC is the standard procedure[19]. While automated segmentation can introduce minor inaccuracies, these are expected to be largely unsystematic across subjects and time points[20–23]and thus more likely to contribute to residual noise rather than systematic bias in the latent growth models[24].

We further mention this limitation explicitly in the revised Discussion section:
"Fourth, we note that automated segmentation approaches used here may be susceptible to segmentation inaccuracies. However, given the longitudinal multi-centre design of this study, manual correction of all segmentations was not feasible, and a visual quality control procedure was thus applied. Future studies with smaller or targeted samples may adopt alternative quality control strategies. Such approaches must carefully

balance potential gains in local anatomical precision against losses in reproducibility, statistical power, and the risk of rater-dependent bias."

R1.7: Flowchart of Subject Selection: Including a CONSORT-style flow diagram or timeline showing participant inclusion and attrition would greatly enhance clarity.

Response: We included a flowchart for transparently reporting selection of eligible subjects, alongside available data per measurement occasion in our three domains of interest (cognition, WMH, and brain morphometric data). We added this flowchart as a new figure in our supplementary material (**Supplementary Figure 1**). We thank the reviewer for the valuable suggestion.

R1.8: Supplementary Tables: The supplementary tables are not presented in the order referenced in the main text. Please revise for consistency and ease of reference.

Response: We thank the reviewer for noticing the erroneous referencing of our Supplementary material. Please note, that we added additional supplementary material in the context of our revision. We overhauled, checked, and corrected the references carefully.

Reviewer #2

R2.0: This study combines cross-sectional and longitudinal analyses to examine the co-evolution of ageing-related atrophy, white matter hyperintensities (WMH), and cognition, together with domain-specific and domain-general contributions of lifestyle and personality to brain-structure–related cognitive ageing. The key findings are:

1. WMH increased and MTLV-ratio declined over time, both indicating structural brain ageing.
2. These two brain measures were interrelated, with baseline WMH predicting greater subsequent atrophy.
3. PACC5 cognitive performance generally improved over time, most likely due to practice effects from repeated testing; however, this improvement was attenuated in individuals with more atrophy and faster WMH progression.
4. Personality and lifestyle factors — particularly neuroticism, depressive symptoms, and low cognitive engagement — were associated with poorer brain maintenance.

Response: We sincerely appreciate the reviewer's thoughtful and constructive feedback. All points raised have been carefully considered and incorporated into the revised manuscript. We believe that these changes, alongside the adjustments made in response to the other reviewers, improve the clarity and transparency of our work. In the updated version, we have added, clarifications, additional explanations, and new material where appropriate. Below, we provide a point-by-point response, indicating the corresponding sections of the revised manuscript.

R2.1: In Section 2.2.1, the authors state: "WMH volumes increased, MTLV-ratios decreased, and PACC5 performance improved throughout the study period." This pattern is unusual in ageing studies and is likely driven by practice effects, where repeated testing enhances performance and masks underlying decline. In contrast, Figure 1D appears to show a decrease in cognition (PACC5) over time. This apparent discrepancy should be clarified, ideally with a more detailed explanation of how the PACC5 trajectories were derived and interpreted in light of practice effects.

Response: We thank the reviewer for this important note and we understand that this observation seems slightly unexpected. First, we would like to emphasize that mean-level improvements in cognitive performance over repeated assessments are a well-established phenomenon in longitudinal ageing research and have been documented across multiple large cohorts and cognitive domains. Studies from ADNI and the Mayo Clinic Study of Aging show that cognitively unimpaired older adults frequently exhibit positive cognitive slopes driven by retest learning, particularly on composite and episodic memory measures[25,26]. Large lifespan studies such as Betula similarly report initial increases in episodic recall at early retest waves[27], and executive and processing-speed tasks also commonly show practice-related gains that can exceed age-related decline at short retest intervals. Longitudinal work using PACC-type composites likewise demonstrates mean-level improvement in amyloid-negative individuals[16]. These observations across cohorts suggest that the small positive PACC5 slope in our sample reflects typical practice effects in healthy ageing rather than a methodological anomaly.

Nonetheless, as the reviewer correctly pointed out, these group-level and individual rates of change over follow-ups are likely to reflect an unknown mixture with contributions from both ageing as well as practice effects. In accordance with similar issues raised by reviewers #1 and #3, we therefore addressed this issue by explicitly incorporating practice effects in our revised model. For details on this procedure, we kindly refer to the comment where this issue first addressed (**R1.2**).

The initial depiction in Figure 1 in our manuscript incorporated both intra-subject time effects (trajectories itself) and the age-related effects on baseline differences (cross-sectional). Illustration of effects of interest can be focused on each separately or even both simultaneously. The initial Figures 1B-D hence reflected complementary aspects, which we understand may have diluted our message.

In our revised manuscript, we now exchanged the figure with an alternative illustration of change over the measurement occasions as change since baseline (see **Response Figure 3)** which is now closer to the modelling approach. Thereby, the global small positive increase in cognitive performance becomes apparent (**Response Figure 3C**). Please note that in this figure we plotted change since baseline (time point 0) by

subtracting baseline estimates from all measurement time points rendering baseline estimates to be 0. We revised the figure legend accordingly:
"(B-D) Change from baseline in the three neurocognitive domains of interest, operationalized by subtracting baseline estimates from all follow-up measurements rendering baseline estimates to be zero. (B) WMH volumes increase over follow-ups. Total WMH volumes were log10-transformed and z-scored. (C) MTLV-ratio decreases over follow-ups. MTLV-ratio was Box-Cox-transformed and z-scored. (D) PACC5 performance weakly but nonsignificantly increases across all participants and follow-ups. PACC5 performance was Yeo-Johnson transformed and z-scored."

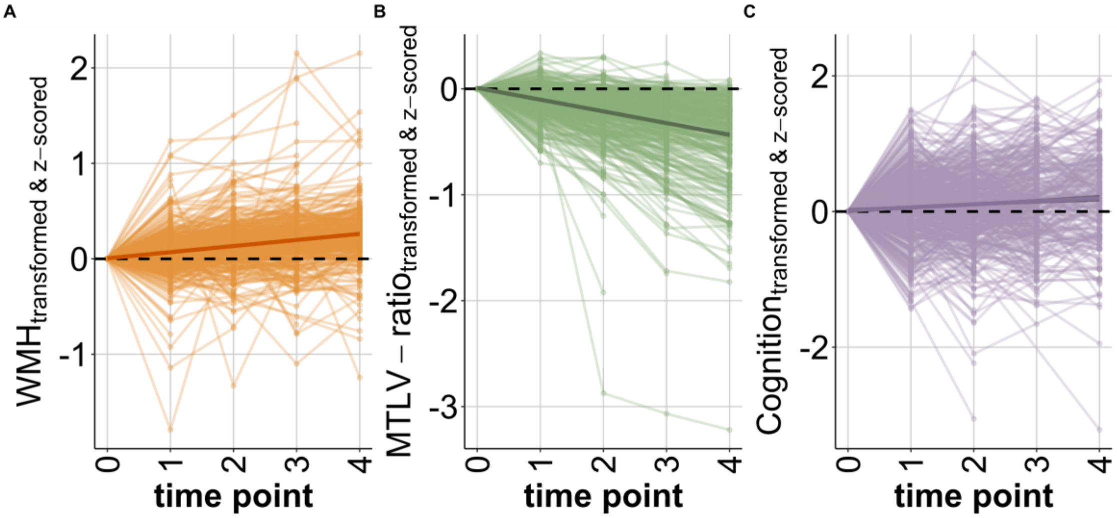

Response Figure 3. Raw change from baseline in the three neurocognitive domains of interest. (A) WMH increase over follow-ups. **(B)** MTLV-ratio decreases over follow-ups. **(C)** PACC5 performance slightly increases across all participant and over follow-ups.

R2.2: The analysis of associations between neurocognitive trajectories and demographic variables (age, sex, years of education) is valuable. In the Education section, participants with more years of education had better baseline PACC5 scores but did not show greater longitudinal improvements in PACC5. I assume these refer to changes in PACC5 over the follow-up years? It would be helpful to clarify this point. Additionally, did you examine whether longitudinal MTLV-ratio changes were associated with education? This could add an interesting dimension to the results.

Response: We thank the reviewer for these considerate questions. We confirm that the association between education and the PACC5 performance changes refers to the longitudinal changes in PACC5 performance over follow-ups represented in the latent slope. The association between education and linear MTLV-ratio decline was also accounted for in the model, but was not significant. We also note, that within our revision, we now included a quadratic effect of time (since baseline) on the MTLV-ratio longitudinally. This decision was based on the goal to explore nonlinearities of trajectories in each cognitive domain, using univariate models, in line with a comment by reviewer #3 (**R3.2**; **Response Figure 2D**).

We appreciate the questions of the reviewer, and acknowledge the lack of information in our previous manuscript. We therefore address it in the following ways.

1) At the beginning of the section, we now point readers to the supplementary table of our main model, where all associations are depicted:
"Supplementary Table 4 provides the full information on all associations regarding age, sex, and years of education and latent intercepts and slopes of the three neurocognitive domains."
2) For clarity, we report the non-significant finding regarding the association between education and linear MTLV-ratio changes. For transparency and readability, we removed the non-significant statistics for the association between education and changes in PACC5 performance over follow-ups. We rephrased the paragraph, which now reads:
"Individuals with more years of education had better initial PACC5 performance (years of education → intercept Cognition: $\beta = 0.257$, $Z = 6.337$, $p < 0.001$), and higher initial MTLV-ratios (years of education → intercept MTLV-ratio: $\beta = 0.103$, $Z = 2.720$, $p = 0.007$). However, years of education did not relate to differential PACC5 performance changes. We observed an education effect on quadratic but not linear MTLV-ratio change, indicating decelerated MTLV-ratio decline with more years of

education (years of education → quadratic MTLV-ratio slope: β = 0.272, Z = 2.291, p = 0.022). As quadratic MTLV-ratio slope variance was not significant, we however advise careful interpretation of this relation. Education did not relate to baseline levels or changes of WMH."

3) We added information on a significant association between years of education and the quadratic MTLV-ratio change, that indicated less acceleration of MTLV-ratio decline with more years of education (*years of education → quadratic slope MTLV-ratio*: β = 0.272, Z = 2.291, p = 0.022). Since quadratic slope variance was not significant (p = 0.079), we however advise careful interpretation of this relation.

4) Our findings are in line with recent reports on the influence of education of cognitive and brain ageing [28,29]. We addressed this also in the discussion. The report on association with education now reads as follows:
"Education only contributed to baseline levels of cognition and MTLV-ratio[51–53]. Although we found that education was associated with the quadratic MTLV-ratio change, indicating less acceleration of MTLV-ratio decline with more years of education, we advise careful interpretation of this relation since quadratic slope variance was not significant. Consistent with preserved differentiation[6], education might hence rather contribute to interindividual differences in baseline cognitive functioning or brain reserve, not their rates of changes"

R2.3: Figure 2 is informative. As I understand it, individuals with faster WMH progression tend to have more negative cognitive slopes (red points lower on the z-axis), and those with steeper MTLV-ratio decline also tend to have more negative cognitive slopes. Individuals who perform better cognitively over time (blue points) generally have slower WMH progression and less atrophy. It may be worth producing an alternative, simplified 2D or clearer 3D plot to make this pattern more apparent to readers.

Response: We thank the reviewer for the helpful suggestion. We now adapted the figure as suggested into two 2D-plots showing the relation between the extracted factor scores of WMH and MTLV-ratio slopes against the change rate of PACC5. We additionally included an interactive HTML-based 3D graphic that is accessible through the supplements (**Supplementary Figure 5**) to retain the common representation between the three neurocognitive domains of interest here in one space.

R2.4: For Figure 3, the current use of filled bars for positive correlations and striped bars for negative correlations could be replaced with a left–right bar format (e.g., bars to the right for positive, to the left for negative) to make the direction of correlations easier to interpret.

Response: We have adapted the figure as suggested by the reviewer and plot the correlations from left-to right (negative to positive). We note that, in accordance to reviewer #3's suggestion, we now use manifestation in lifestyle factors from baseline only (**R3.7**). We exchanged plots in the main manuscript alongside those in the supplementary material in the same style. We thank the reviewer for this constructive suggestion.

R2.5: The study includes plasma Aβ42/40 as a biomarker. Since other plasma biomarkers such as p-tau217, GFAP, and NfL are increasingly recognized for their value in detecting Alzheimer's disease pathology, could the authors clarify why only Aβ42/40 was included in the present analysis?

Response: We thank the reviewer for this important issue. Our initial goal to assess plasma Aβ42/40 was based on previous work linking WMH and atrophy in light of AD-pathological change, particularly Amyloid accumulation. Moreover, we assumed that baseline plasma levels of Aβ42/40 might influence the manifestation of these markers at baseline and possibly also affect trajectories of those, as suggested in literature. Yet, we acknowledge, that other biomarkers have recently attracted more attention and have moved the field in a new direction. The DELCODE cohort additionally includes measurements of plasma biomarkers NfL and pTau181. Recent work from other groups within the DELCODE study group showed that these biomarkers appeared eligible in preclinical stages and at risk for cognitive decline[30]. In line with the reviewer's suggestion, we added new analyses testing for effects of plasma ptau181 levels and NfL levels on intercepts and slopes of our three neurocognitive domains of our global model and whether they might affect the latent covariance structure – in the same way as we tested for plasma Aβ42/40. We included the necessary information to the revised manuscript and further methodological details and discussion in the supplementary material.

Including plasma pTau181 or NfL did not alter associations and interpretation of associations between latent variables. Interestingly, we observed that higher levels of plasma pTau181 were associated with lower

PACC5 baseline performance and faster cognitive decline, as well as with smaller baseline MTLV-ratio and MTLV-ratio decline. Higher levels of plasma NfL were related to faster PACC5 performance decline, to higher baseline volumes of total WMH, and smaller MTLV-ratio at baseline. We now report these novel findings regarding hidden pathology on neurocognitive trajectories in the revised result section of the manuscript and added full information on models as additional analyses alongside discussion in our supplements (**Supplementary Table 7-10**).

"We tested for effects of assumed shared risk factors by including cardiovascular risk, APOE-ε4 and plasma Aβ42/40, plasma pTau181, and NfL into the global model separately (Supplementary Figure 3; Supplementary Table 7-10). Cardiovascular risk was weakly related to higher baseline WMH volumes (β = 0.078, Z = 1.968, p = 0.049). While plasma Aβ42/40 did not relate to any of the three neurocognitive domains, APOE-ε4 carriership was related to stronger linear MTLV-ratio decline (β = -0.208, Z = -2.996, p = 0.003). Higher levels of plasma pTau181 and plasma NfL were related to smaller baseline MTLV-ratio (plasma pTau181: β = -0.073, Z = -2.228, p = 0.026; NfL: β = -0.123, Z = -3.00, p = 0.003). Higher plasma pTau181were also linked to stronger linear MTLV-ratio decline (β = -0.173, Z = -2.674, p = 0.007). Worse baseline PACC5 performance was related to higher plasma pTau181 levels (β = -0.092, Z = -2.603, p = 0.09). Moreover, both plasma pTau181 and plasma NfL related to stronger cognitive decline as assessed via the PACC5 (plasma pTau181: β = -0.249, Z = -3.239, p = 0.001; NfL: β = -0.269, Z = -3.055, p = 0.002). Nfl was additionally associated with baseline WMH volumes (β = 0.080, Z = 2.266, p = 0.023). Yet, accounting for these additional covariates neither significantly improved model fit nor altered the interpretation of the associations of interest regarding relations across latent variables (Supplementary Table 7-10). Therefore, we present and interpret the results of the parsimonious model, including only the covariates age, sex, years of education, and total intracranial volume (TICV)."

and

"We note, that while we observed that blood-based biomarkers pTau181 and NfL were related to smaller MTLV-ratios, steeper decline thereof, higher WMH volumes at baseline, and to more pronounced cognitive decline[39,40], they did not alter the latent associations across neurocognitive domains. Further discussion regarding hidden pathology is provided in the supplements (following Supplementary Tables 7-10)"

Additionally, we extended our discussion accordingly and in light of the more recently developed plasma biomarkers, which were unfortunately not available in DELCODE, but have proven to be sensitive to AD-pathological change:

"Fifth, while our analysis was based on a specific operationalization of the three neurocognitive domains, future research may benefit from further examining lesion load within specific hubs of cognitive networks[46], clarify the differential relevance of various CSVD markers, integrating more recently established blood biomarkers alongside biological staging of AD, and expand the cognitive domains studied[16,17,35,42]."

R2.6: Beyond total WMH volume, did you explore periventricular WMH (PVWMH) and deep WMH (DWMH) separately? These two WMH subtypes have distinct neuropathological underpinnings, risk factors, and cognitive associations, and their separate analysis might yield additional insights.

Response: We thank the reviewer for raising the important issue of potential region-specific effects on WMH. Indeed, distinctions such as PVWMH vs. DWMH, anterior–posterior gradients, or lobar WMH have been proposed, though no clear consensus currently exists on which parcellation best captures etiological or cognitive relevance in ageing[31]. Regional WMH analyses are further complicated by higher outcome dimensionality and reduced statistical power due to required multiple comparison corrections and expected increased noise levels when using smaller ROIs, particularly in healthy ageing samples with low vascular burden.

Our study focused on modelling cross-domain coupling of longitudinal change and on quantifying brain maintenance, for which a global WMH index is most robust and statistically efficient. Nonetheless, in response to the reviewer, we conducted additional regional analyses aligned with theoretically motivated distinctions observed in the literature. Prior work – including our own – suggests that posterior WMH are more closely linked to AD-pathology such as amyloid[32], whereas frontal WMH are more associated with vascular risk and executive dysfunction. Analogously, frontal and parietal cortices differ in ageing vulnerability[33,34] and cognitive relevance.

Therefore, we analysed frontal vs. posterior WMH jointly with frontal vs. parietal cortical volumes in two trivariate LGCMs using analogous regional definitions[35] and FDR correction for the 12 cross-domain parameters of interest per model. Across both regional models, WMH increased and cortical volumes declined, but no WMH–cortex associations survived FDR correction, and WMH changes did not predict cognitive change. Only weak, isolated effects appeared at the uncorrected level (e.g., baseline cognition predicting less cortical decline). These regional patterns differ from the global trivariate model, where aggregated WMH and MTLV-ratio showed robust coupling and jointly predicted cognitive change.

Taken together, these results indicate that in cognitively unimpaired individuals with modest WMH burden, regional WMH variations are too subtle to meaningfully relate to regional cortical atrophy or cognitive change. This is consistent with prior population-based studies showing that regional WMH effects become detectable primarily in more advanced CSVD or larger samples[36,37]. In contrast, the MTLV-ratio which integrates AD-sensitive structures and ventricular enlargement[38], is more responsive to early ageing- and cerebrovascular-related variations, explaining why the global model captures meaningful cross-domain coupling whereas regional analyses do not.

For transparency, we now briefly report these regional findings in the supplemental material, include a supplementary figure comparing effect sizes across the global and regional models (**Supplementary Figure 2**), and provide full model details (**Supplementary Tables 5&6**). We clarify that LGCM is currently not well suited for finer granularity regional modelling and that theoretically motivated aggregation remains a useful working assumption. More powerful explorative studies might have higher sensitivity to explore those regional differences further.

We describe the rationale of the regional analyses in the methods
(4.5.2): *"Additionally, we employed two regional-level trivariate LGCM involving frontal WMH and cortical volume, or posterior WMH and parietal cortical volume, respectively. We opted for complementary analysis of features in these analogous regions for the following reasons: First, to ensure comparability, avoiding mixture of conceptually different regions. Second, similar to potential insights into underlying pathology by the anterior-posterior WMH distribution, atrophy in both frontal and parietal regions is driven by ageing, but parietal regions are also vulnerable to e.g. AD-pathological change[14,15,85]. Third, by distinguishing anterior-posterior processes, we sought to capture a frontal more vascular dependent, and a posterior more AD-pathology dependent pathway to accelerated neurocognitive ageing. While frontal regions were linked more closely to executive functions, parietal regions appear to be more relevant for memory performance. We hence assumed that associations with PACC5 – primarily reflecting memory, but also including executive components – may be stronger for parietal cortical volume changes and posterior WMH progression than for the same processes in frontal regions. We accounted for multiple comparisons by FDR-correction for 12 cross-domain parameters of interest per model."*

and

(4.2.2): *"Moreover, we estimated frontal and parietal cortical volume, as corresponding regions to frontal and posterior WMH. Frontal and parietal cortical volumes were defined as aggerate volumes across hemispheres based on Freesurfer's automatic volumetric cortical parcellation, in accordance with the original suggestions by Desikan et al.[80] (Table 3)."*

We point the reader to these regional results adding the following statement to our results section:
"Results of regional-level analyses are reported in Supplementary Table 5&6 and Supplementary Figure 2."

We also tested for regional effects of lifestyle factors and point to the results:
"We show associations between personality and lifestyle factors and regional WMH and cortical volumes in Supplementary Figures 11 & 12."

To the discussion we added information on regional WMH progression and cortical volume decline, but emphasise the absence of cross-domain relations on a regional level:
"With regards to the temporal associations of the neurocognitive domains of interest, we note that there were no prominent regional-specific associations. Therefore, we mainly focus on the implications of the trivariate associations identified by our global model."

and

"Conversely, regional levels and progression of WMH did not relate to cognitive changes, suggesting that total volume of WMH in this sample with relatively low cardiovascular risk might better reflect the cerebrovascular as well as general pathological risk burden. With regard to MTLV-ratio, both baseline levels and decline were associated with cognitive performance changes, underlining the importance of MTL integrity in maintaining cognitive function in ageing[3]. In contrast, regional atrophy did not relate to cognitive changes, as reported elsewhere[24]."

Reviewer #3

R3.0: Brain maintenance, also called brain reserve, has been of increasing interest in the research of cognitive aging and dementia prevention. Here, Menze et al. provided new insights on the issue by investigating how ageing-related atrophy (measured as medial temporal lobe to ventricle ratio [MTLV-ratio]) and white matter hyperintensities (WMH) co-evolved with each other and with cognitive ageing over four years. Based on a large sample of cognitively unpaired older adults, they found that steeper MTLV-ratio decline related to baseline WMH volume and its progression, and changes in MTLV-ratio and WMH volume independently contributed to cognitive changes. Besides, several lifestyle and personality factors were found to be related to unfavorable trajectories and brain maintenance index. The topic of this paper is of great interest to the field, but there were several issues in this manuscript that were difficult to follow at times and required some clarifications.

Response: We thank the reviewer for the encouraging evaluation of our work and for emphasising its significance to the field. We also appreciate the thorough assessment of our manuscript and their valuable suggestions and helpful criticism of this work. We addressed the reviewer's comments and believe that – in accordance with the feedback of the other reviewers – they enhance the transparency of our manuscript. In the revised version of the manuscript, we have included additional statements, clarifications and new material. In the following, we will address each comment separately and point to the respective:

R3.1: The logic of Introduction part made me confused, brain maintenance should be highlighted at the very beginning of the manuscript, rather than ageing-related structural changes or cerebrovascular abnormalities. Furthermore, several statements should be modified to fit the purpose of the study, like Line 116-117, Line 135-136, current study neither aimed to elucidate the factors underlying the between-subject variability in contribution from structural and cerebrovascular brain changes to cognitive ageing, nor the nature of their interrelationships.

Response: We thank the reviewer for the valuable comments regarding our introduction. We agree that the initial construction might have been less focused and less stringent to follow with the purpose of the manuscript into elucidating interrelated changes of neurocognitive domains to elucidate brain maintenance.

We therefore completely rewrote large parts of the introduction. We also removed the misleading statements to focus and streamline the study goal. We believe that the overhauled introduction provides the reader with a more coherent, concise, and convincing logic that leads more understandably to the goal of our study.

Particularly, we added the following changes, first by opening the introduction by stating:
"*Although declines in cognitive abilities and brain integrity are expected with ageing[1,2], some individuals preserve brain structure relevant to cognition for years, owing to their inherent or acquired capacity to attenuate ageing- and pathology-related changes[3–7]. This phenomenon, known as brain maintenance[8,9], remains incompletely understood but represents a major research priority in neurocognitive ageing, given its implications for successful ageing and its relevance for preventive and therapeutic strategies.*
From a modelling perspective, characterising brain maintenance requires capturing the coupled changes between brain integrity and cognitive function over time, i.e. the extent to which cognition is preserved because the brain itself remains structurally and functionally intact. Meeting this theoretical requirement entails[9]: (i) identifying neural alterations that accompany ageing and contribute to cognitive decline; (ii) adopting modelling frameworks capable of capturing system-level dynamics, in which longitudinal interrelationships between cognitive and brain processes are jointly modelled rather than treated as independent trajectories; (iii) isolating the specific component of cognitive ageing attributable to preserved brain structure; (iv) and identifying factors that contribute to brain changes in order to maintain brain health and consequently cognitive outcomes. Recently those particularly entail modifiable lifestyle factors, including cognitive, social, and physical activity, cardiovascular risk factors, dietary patterns, as well as psychological risk factors like depression, which all were linked to cognitive and brain health outcomes [10–12]."

After elaborating on the relations between WMH and atrophy and their role in cognitive ageing as approaches for the first and second requirement to test brain maintenance, we more clearly emphasise the objective of our study and its novelty contributing to the operationalisation of brain maintenance:

"To our knowledge, the third requirement of the brain maintenance framework—isolating the component of cognitive ageing attributable specifically to structural preservation[9]—has not yet been explicitly addressed. We therefore set out to achieve three complementary objectives. First, we modelled coupled longitudinal changes across cerebrovascular pathology, brain atrophy, and cognition within a unified multivariate latent growth curve modelling (LGCM) framework. We thus extend prior work on pairwise associations by a parsimonious model able to validate global fit of the coupled system. Second, we derived individual cognitive trajectories as a function of joint structural change, enabling explicit quantification of the proportion of cognitive ageing explained by preserved brain integrity. This provides a principled operationalisation of brain maintenance as a longitudinal, multi-domain, system-level construct. Third, we identified modifiable lifestyle factors and personality traits that contribute to inter-individual differences in baseline levels and longitudinal change across neurocognitive domains and our proposed domain-general brain maintenance index.
We demonstrate the utility of this framework in a large cohort of cognitively unimpaired older adults followed annually over four years, yielding novel mechanistic insight into the neurobiological basis of brain maintenance and identifying potential targets for preventive strategies against cognitive decline."

R3.2: Towards the core statistical analysis, the latent growth curve modelling, did the authors have tried nonlinear modelling on latent slopes for the MTLV-ratio, WMH, and PACC5 performance?

Response: We thank the reviewer for these valuable suggestions. First, we addressed the exploration of possible nonlinear temporal dynamics in each of the three domains. In context of reviewer #1's comment (**R1.3**), we tested for potential nonlinear effects of time, specifically by including quadratic effects of time using separate models per domain. We tested the corresponding model fit and compared those against models including a linear effect of time only, vs. the subsequent model including both linear and quadratic effects of time via Satorra–Bentler scaled chi-square difference test.

Following usual conventions in LGCM, the quadratic effects of time was defined by fixed raw polynomial loadings (linear: 0,1,2,3,4; quadratic: 0,1,4,9,16), reflecting annual change and potential trajectory curvature over the five measurement occasions. This parametrization was chosen to preserve interpretability of slopes as biologically meaningful rates of change over time. The same covariates were included as in our original model (age, sex, years of education, and in case of WMH and brain morphometric measures TICV). We tested for covariance between all latent variables (intercept, linear, and quadratic slope) and observed the following: In the LGCM on cognitive performance (PACC5), including a quadratic effect of time led to model convergence issues (negative variance in the quadratic terms). Specifying uncorrelated linear and quadratic time effects resolved the issue. Yet, adding a quadratic effect to the LGCM for cognitive performance, did not significantly improve model fit ($\Delta\chi2$ =9.382, $\Delta df = 6$, $p = 0.153$). Moreover, there was no significant variability in the quadratic effects (B = 0.404, Z = 0.148, p = 0.833), while mean quadratic coefficient estimates across all individuals was significant though small (B = 0.03, Z = 2.700, p = 0.007). We therefore assume that in this sample, a linear approximation of cognitive performance changes is a reasonable assumption given our sample.

The LGCM on WMH resulted in a similar convergence issue, when fitting a model including a quadratic time effect (negative variance in the quadratic slope). When fixing the quadratic time effect's variance to 0, or a small positive number (0.0001), the model converged, however, it was not superior to the more parsimonious model including a linear slope only ($\Delta\chi2$ =0.93, $\Delta df = 7$, $p = 0.996$). We also tested possible quadratic effects of time on the additionally included frontal and posterior WMH volumes. While for frontal WMH, a model specifying uncorrelated linear and quadratic effects of time converged, it did not outperform a model including a linear slope ($\Delta\chi2$ = 13.415, $\Delta df = 7$, $p = 0.063$). As for posterior WMH, we found that a model including a quadratic effect of time did not fit the data better ($\Delta\chi2$ =7.91, $\Delta df = 8$, $p = 0.443$), Thus, we decided to accept the more parsimonious linear model.

Regarding MTLV-ratio, we found that introducing a quadratic effects of time increased model fit ($\Delta\chi2 = 27.503$, $\Delta df = 8$, $p < 0.001$; **Response Figure 2C&D**; [39], with a significant mean quadratic changes ($B = -0.439$, $Z = -3.881$, $p < 0.001$), and a trend for significant interindividual variability in the quadratic time coefficient (B = 0.909, Z = 1.752, p = 0.080). There was a significant effect of years of education on the quadratic effect of time (B = 0.277, Z = 2.338, p = 0.019), indicating a less accelerating decline – hence possibly protective effect – of education. Adding a quadratic effect of time to the model on frontal cortical volume ($\Delta\chi2 = 12.73$, $\Delta df = 8$, $p = 0.122$) or parietal cortical volume ($\Delta\chi2 = 12.367$, $\Delta df = 8$, $p = 0.136$) did not enhance model fit.

In summary, we therefore retained linear slopes only in the regional trivariate models. With regards to the global model – encompassing total WMH, MTLV-ratio, and PACC5 performance – we updated the model and included a quadratic effect of time for MTLV-ratio. Overall, we found that our covariance pattern remained stable, with cognitive changes associating with baseline levels of MTLV-ratio (B = 0.259, Z = 2.838, p = 0.005), linear MTLV-ratio decline (B = 0.354, Z = 2.439, p = 0.015), and WMH progression (B = -0.208, Z = -2.010, p = 0.044). However, direct association between WMH progression and linear MTLV-ratio decline diminished (B = -0.130, Z = -1.450, p = 0.147), while the relation between baseline WMH and baseline MTLV-ratio (B = 0.136, Z = -3.195, p = 0.001) as well as linear MTLV-ratio decline (B = -0.186, Z = -2.734, p = 0.006) remained significant.

We added methodological information before presentation of results and in our methods, stating:
Results: *"Before specifying each trivariate model, we characterized mean change processes within each domain (see methods 4.5.2 & Supplementary Table 3). While linear latent change adequately captured trajectories of total and regional WMH, frontal and parietal cortical volumes, as well as cognition, decline in MTLV-ratio required both linear and non-linear latent change components."*

and

Methods: *"Before fitting the trivariate models, we fitted separate univariate LGCM to examine the possibility of nonlinear slopes for each feature per domain. For each domain, we compared a model with a linear slope only against a model including both linear and quadratic slopes using Satorra-Bentler scaled χ^2 difference test. Linear slopes denoted rates of change per year, while quadratic slopes would indicate acceleration of changes. Only for MTLV-ratio, including a quadratic slope increased model fit significantly ($\Delta\chi 2 = 27.503$, $\Delta df= 8$, $p < 0.001$; Supplementary Table 3). In all other dimensions, linear slopes sufficed. Therefore, the global model encompassing total WMH, MTLV-ratio, and PACC5 included a quadratic slope alongside the linear slope for MTLV-ratio, while regional models were fitted with linear slopes only on all dimensions."*

We briefly discuss the new finding of no association between WMH progression and linear MTLV-ratio decline in the discussion stating:
"While we observed significant associations between baseline total WMH and MTLV-ratio, even when controlling for shared risk factors[23,25,41], their rates of change were not related, in contrast to previous reports[4,25,42–44]. Yet, the absence of such associations has also been observed in relatively healthy ageing cohorts[45], comparable to the present sample."

R3.3: … Besides, I would suggest that the authors conduct further moderation or mediation analysis between the three domains to get deeper information on their co-evolution relationship, rather than just simple correlation.

Response: We very much appreciate the reviewer's suggestion to explore the intriguing hypothesis of mediation among the three neurocognitive domains. Conceptually, statistical mediation is often motivated by the notion of a temporal or mechanistic/causal sequence between processes, while the applied LGCM captures associations of certain aspects of change trajectories across the same study time frame, with slope-covariances suggesting their co-evolution in terms of associated change parameters of two domains across subjects. Translating such slope-slope associations into mediation should therefore be conducted and interpreted with caution (abstaining from potential causal claims) since all slopes do represent a change parametrization of the entire trajectory and not sequential change from one time point to another. As discussed, other models such as dual or multivariate latent change score models are more suitable for testing such lead-lag associations in the SEM framework [40–42]. In a statistical sense mediation is often conceptualized cross-sectionally in terms of a specific hypothesis of shared individual differences of a candidate mediator with a predictor and an outcome (typically cognition) and their respective direct and indirect regression paths. Although many variations of this exists, also for longitudinal mediation models, we here focussed our new analyses to the following form.

In response to the reviewer's comment, we specified a mediation analysis, building on the theoretical assumption that WMH progression contributes to accelerated atrophy, ultimately leading to faster cognitive decline, as stated in our discussion[43,44]. To facilitate interpretation, we implemented the mediation model using the extracted factor scores of the LGCM. In detail, we aimed for a mediation model (**Response Figure 4**) implemented in lavaan, using bias corrected bootstrapping (5000 iterations). The model suggested stronger

cognitive decline with increased WMH progression (path c) and MTLV-ratio decline (path b), however, there was no evidence for a mediation effect of WMH via MTLV-ratio decline (path a*b).

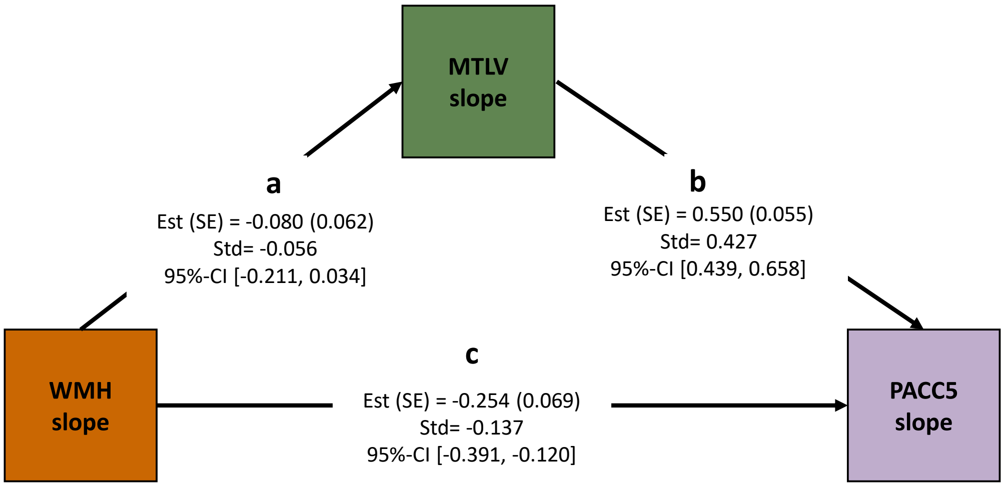

effects	Est	SE	Std. Est	95%-CI
a*b	-0.044	0.035	-0.024	-0.117, 0.018
**total**	**-0.298**	**0.072**	**-0.161**	**-0.449, -0.163**

Response Figure 4. Mediation of WMH on ageing-related atrophy, driving cognitive changes.

Overall, the mediation model – while formally relevant – did not add substantively new insights beyond the co-developmental associations already captured by the LGCM. There, we also observed that changes in WMH and MTLV-ratio covaried with PACC5, yet these two domains did not co-evolve over time. Rather, higher intercepts of WMH volumes were linked to faster MTLV-ratio decline. We therefore opted not to include this additional mediation analysis in the manuscript, particularly given the interpretational limit of mediation among slopes estimated over the same time frame, as well as for reasons of space limitations.

Finally, we note that moderation in the context of this hypothesis was tested by the interaction effect in our multiple linear regression model i.e. predicting cognitive slopes using brain slopes and their respective interaction. This interaction did however not reach significance in our sample (see Results section 2.2.4 in our manuscript). A significant interaction effect between MTLV-ratio change and WMH change on cognitive performance changes would have indicated, that the effect of atrophy on cognition would depend on the manifestation of change in WMH (or the effect of WMH on cognition would depend on manifestation of ageing-related atrophy), ultimately pointing to synergistic effects of both processes. Yet, our analysis revealed that additive effects of WMH changes and ageing-related atrophy are more likely in our sample.

We emphasize exploration of possible mediation and conceivable moderation effects for future studies more clearly in the limitations part of our discussion, by including the following statements:

"Exploring the consequences and underlying causes of interindividual variability in trajectories of WMH and ageing-related atrophy—and scenarios in which cognitive function remains stable despite structural brain changes—will ultimately inform our understanding of cognitive reserve and brain maintenance[9]. They might require more sophisticated longitudinal analysis approaches in large samples in a lead-lag fashion to explore possible mediation effects."

and

"Several limitations of this study warrant consideration. First, although LGCMs can determine the co-evolution of constructs, they cannot assess the delayed effect of change in one construct on change in another at a later time point. While mediation and moderation hypotheses regarding the temporal sequencing of the

three neurocognitive domains are compelling, their evaluation requires longitudinal frameworks that explicitly model lead-lag relationships. Other frameworks, such as latent change score models[66–68], could enable more nuanced insights into such causal sequences"

R3.3: More explanations on operational definitions of brain-related domains are needed. The study focused on cognitively unimpaired older adults and the PACC5 was a general examination on cognitive function, why were only MTL regions chose to calculate the MTLV-ratio? How about the frontal and parietal regions? Similar issue existed for the WHM. although the authors discussed that 'our focus on global WMH precludes addressing potential regional associations with ageing-related atrophy', an inclusion of regions WMH would be able to establish a dedicated, more specific relationship between brain atrophy (MTLV-ratio or other) and WMH.

Response: This important comment was also raised by reviewers #1 and #2. We understand that the global approach leaves certain questions to the reader that are intriguing in the context of ageing. We therefore implemented additional regional analyses, including frontal and parietal lobes, alongside frontal and posterior WMH. For details on our argumentation and the procedure, we kindly refer to the comment where this issue is first addressed (**R2.6**). Moreover, we more clearly highlight our choice of using the PACC5, while also transparently discussing alternatives, in line with a comment by reviewer #1's (**R1.2**).

R3.4: The potential practice effects of PACC5 performance (Line 198-200) could be separated from ageing-related changes by statistical modelling, referred to studies like Rabbitt et al., 2001 and Myrskylä et al., 2024. *Reference: Rabbitt, P., Diggle, P., Smith, D., Holland, F., & Mc Innes, L. (2001). Identifying and separating the effects of practice and of cognitive ageing during a large longitudinal study of elderly community residents. Neuropsychologia, 39(5), 532-543.*

Myrskylä, M., Hale, J. M., Schneider, D. C., & Mehta, N. K. (2024). Trends in Memory Function and Memory Impairment Among Older Adults in the United States and Europe, 1996–2018. The Journals of Gerontology, Series A: Biological Sciences and Medical Sciences, 79(Supplement_1), S11-S21.

Response: We thank the reviewer for this thoughtful comment and their recommendations on addressing practice effects. We approached this issue by implementing a model-based statistical solution inspired by Ferrer et al. [11] and Hoffman et al [12]. The approach separates retest-related practice effects from ageing-related change within the structural equation modelling framework. For details on our approach, we kindly refer to the previous comment where this issue was first addressed (**R1.2**).

R3.5: Please provide the distribution plots of the original data of WMH volumes, MTLV-ratio, and PACC5 performance.

Response: We now provide ridge plots of the original distributions across all assessment time points of the three neurocognitive domains in our supplementary material (**Supplementary Figure 13**), which we refer to in the methods section. We thank the reviewer for this helpful suggestion.

R3.6: How many participants have converted to MCI or dementia during the follow-ups? The number was not consistent in manuscript (n=93, Line 625) compared to the one in supplementary file (n = 69).

Response: Thank you for noticing this erroneous inconsistency. We corrected the number. In total 93 individuals converted to MCI or dementia, while 413 remained cognitively stable. The assessment of conversion status for the remaining 37 was not available or out of the time range after baseline assessment.

R3.7: for the modifiable lifestyle factors and personality traits, I prefer not using the means of all available measurements as the manifestations of the lifestyle factors, since changes on those factors might related to MCI/dementia conversion or other health conditions.

Response: We acknowledge the important concern raised by the reviewer. Our initial motivation was to account for random fluctuations across measurements, since the assessment period was relatively short (4 years). However, indeed subtle changes in lifestyle factors may relate to conversion, or other emerging health conditions over time. As such, we adapted these analyses accordingly and included only baseline measurements of personality and lifestyle factors. The interpretation will hence be clearly focused on the

question which influence the manifestation in personality and lifestyle factors at a specific time point will have on changes in the neurocognitive domains.

Overall, the associations between neurocognitive changes and most lifestyle or personality factors did not alter substantially. We still observed significant effects of GDS, neuroticism, and LEQ unspecific scores in young adulthood and middle-age. However, associations with the LEQ specific scores in higher age were not significant anymore. These associations diminished to weaker trends, which did not survive FDR-correction ($r_{slope\ cognition \sim LEQh\ specifc}$ = 0.09, p = 0.051 p_{FDR} = 0.250; $r_{slope\ cognition \sim LEQh\ specifc}$ = 0.07, p = 0.101 p_{FDR} = 0.406). We believe that this might be due to loss in power, since the aggregate scores of LEQ_h across time was available for 534 individuals, while only 414 individuals had available data for $LEQ_{h\ specific}$ and 467 for $LEQ_{h\ unspecific}$ at baseline.

Please note, that we also tested and added associations between lifestyle and personality factors and regional WMH and cortical volumes in our supplementary material (**Supplementary Figures 11 & 12**).

R3.8: Typos? at Line 253, covstandaridzed = -0.181, where number in the supplementary table 3 was -0.281.

Response: Thank you for noticing and pointing out this error. We appreciate the thorough assessment of our manuscript and accompanying supplementary material. Indeed, this was a typo mistake. Please note that with our overhauled the analysis, the results have now changed.

References

1. Fornell, C. & Yi, Y. Assumptions of the Two-Step Approach to Latent Variable Modeling. *Sociol. Methods Res.* **20**, 291–320 (1992).
2. Muthén, B. O. & Curran, P. J. General longitudinal modeling of individual differences in experimental designs: A latent variable framework for analysis and power estimation. *Psychol. Methods* **2**, 371–402 (1997).
3. Burt, R. S. Interpretational Confounding of Unobserved Variables in Structural Equation Models. *Sociol. Methods Res.* **5**, 3–52 (1976).
4. Stern, Y. *et al.* A framework for concepts of reserve and resilience in aging. *Neurobiol. Aging* **124**, 100–103 (2023).
5. Cole, J. H. *et al.* Brain age predicts mortality. *Mol. Psychiatry* **23**, 1385–1392 (2018).
6. Cahill, S., Hager, R. & Chandola, T. The validity of the residuals approach to measuring resilience to adverse childhood experiences. *Child Adolesc. Psychiatry Ment. Health* **16**, 18 (2022).
7. Song, S., Stern, Y. & Gu, Y. Modifiable lifestyle factors and cognitive reserve: A systematic review of current evidence. *Ageing Res. Rev.* **74**, 101551 (2022).
8. Vidal-Pineiro, D. *et al.* Individual variations in 'brain age' relate to early-life factors more than to longitudinal brain change. *Elife* **10**, (2021).
9. Belsky, D. W. *et al.* DunedinPACE, a DNA methylation biomarker of the pace of aging. *Elife* **11**, 1–26 (2022).
10. MacAulay, R. K. *et al.* Understanding heterogeneity in older adults: Latent growth curve modeling of cognitive functioning. *J. Clin. Exp. Neuropsychol.* **40**, 292–302 (2018).
11. Ferrer, E., Salthouse, T. A., McArdle, J. J., Stewart, W. F. & Schwartz, B. S. Multivariate Modeling of Age and Retest in Longitudinal Studies of Cognitive Abilities. *Psychol. Aging* **20**, 412–422 (2005).
12. Hoffman, L., Hofer, S. M. & Sliwinski, M. J. On the confounds among retest gains and age-cohort differences in the estimation of within-person change in longitudinal studies: A simulation study. *Psychol. Aging* **26**, 778–791 (2011).
13. Lövdén, M., Bergman, L., Adolfsson, R., Lindenberger, U. & Nilsson, L.-G. Studying Individual Aging in an Interindividual Context: Typical Paths of Age-Related, Dementia-Related, and Mortality-Related Cognitive Development in Old Age. *Psychol. Aging* **20**, 303–316 (2005).
14. Jonaitis, E. M. *et al.* Measuring longitudinal cognition: Individual tests versus composites. *Alzheimer's & Dementia: Diagnosis, Assessment & Disease Monitoring* **11**, 74–84 (2019).
15. Langbaum, J. B. *et al.* An empirically derived composite cognitive test score with improved power to track and evaluate treatments for preclinical Alzheimer's disease. *Alzheimer's & Dementia* **10**, 666–674 (2014).
16. Mormino, E. C. *et al.* Early and late change on the preclinical Alzheimer's cognitive composite in clinically normal older individuals with elevated amyloid β. *Alzheimer's and Dementia* **13**, 1004–1012 (2017).
17. Nichols, T. E. *et al.* Best practices in data analysis and sharing in neuroimaging using MRI. *Nat. Neurosci.* **20**, 299–303 (2017).
18. Schermelleh-Engel, K., Moosbrugger, H. & Müller, H. Evaluating the fit of structural equation models: Tests of significance and descriptive goodness-of-fit measures. *MPR-online* **8**, 23–74 (2003).
19. Marcus, D. S. *et al.* Human Connectome Project informatics: Quality control, database services, and data visualization. *Neuroimage* **80**, 202–219 (2013).
20. Han, X. *et al.* Reliability of MRI-derived measurements of human cerebral cortical thickness: The effects of field strength, scanner upgrade and manufacturer. *Neuroimage* **32**, 180–194 (2006).
21. Wonderlick, J. S. *et al.* Reliability of MRI-derived cortical and subcortical morphometric measures: Effects of pulse sequence, voxel geometry, and parallel imaging. *Neuroimage* **44**, 1324–1333 (2009).
22. Alfaro-Almagro, F. *et al.* Image processing and Quality Control for the first 10,000 brain imaging datasets from UK Biobank. *Neuroimage* **166**, 400–424 (2018).
23. Reuter, M., Schmansky, N. J., Rosas, H. D. & Fischl, B. Within-subject template estimation for unbiased longitudinal image analysis. *Neuroimage* **61**, 1402–1418 (2012).
24. Jovicich, J. *et al.* Brain morphometry reproducibility in multi-center 3T MRI studies: A comparison of cross-sectional and longitudinal segmentations. *Neuroimage* **83**, 472–484 (2013).
25. Hassenstab, J. *et al.* Absence of practice effects in preclinical Alzheimer's disease. *Neuropsychology* **29**, 940–948 (2015).

26. Machulda, M. M. *et al.* Practice effects and longitudinal cognitive change in normal aging vs. Incident mild cognitive impairment and dementia in the mayo clinic study of aging. *Clinical Neuropsychologist* **27**, 1247–1264 (2013).
27. Rönnlund, M., Nyberg, L., Bäckman, L. & Nilsson, L.-G. Stability, Growth, and Decline in Adult Life Span Development of Declarative Memory: Cross-Sectional and Longitudinal Data From a Population-Based Study. *Psychol. Aging* **20**, 3–18 (2005).
28. Judd, N. & Kievit, R. No effect of additional education on long-term brain structure, a preregistered natural experiment in thousands of individuals. *Elife* **13**, (2025).
29. Nyberg, L. *et al.* Educational attainment does not influence brain aging. *Proceedings of the National Academy of Sciences* **118**, 1–3 (2021).
30. Mengel, D. *et al.* Blood biomarkers confirm subjective cognitive decline (SCD) as a distinct molecular and clinical stage within the NIA-AA framework of Alzheimer´s disease. *Mol. Psychiatry* **30**, 3150–3159 (2025).
31. Jiménez-Balado, J., Corlier, F., Habeck, C., Stern, Y. & Eich, T. Effects of white matter hyperintensities distribution and clustering on late-life cognitive impairment. *Sci. Rep.* **12**, 1955 (2022).
32. Bernal, J. *et al.* Arterial hypertension and β-amyloid accumulation have spatially overlapping effects on posterior white matter hyperintensity volume: a cross-sectional study. *Alzheimers Res. Ther.* **15**, 97 (2023).
33. Pfefferbaum, A. *et al.* Variation in longitudinal trajectories of regional brain volumes of healthy men and women (ages 10 to 85years) measured with atlas-based parcellation of MRI. *Neuroimage* **65**, 176–193 (2013).
34. Fujita, S. *et al.* Characterization of Brain Volume Changes in Aging Individuals With Normal Cognition Using Serial Magnetic Resonance Imaging. *JAMA Netw. Open* **6**, E2318153 (2023).
35. Desikan, R. S. *et al.* An automated labeling system for subdividing the human cerebral cortex on MRI scans into gyral based regions of interest. *Neuroimage* **31**, 968–980 (2006).
36. Habes, M. *et al.* White matter hyperintensities and imaging patterns of brain ageing in the general population. *Brain* **139**, 1164–1179 (2016).
37. Membreno, R. *et al.* Regional White Matter Hyperintensities Relate to Specific Cognitive Abilities in Older Adults Without Dementia. *Alzheimer Dis. Assoc. Disord.* **37**, 303–309 (2023).
38. Jochems, A. C. C. *et al.* Contribution of white matter hyperintensities to ventricular enlargement in older adults. *Neuroimage Clin.* **34**, 103019 (2022).
39. Ziegler, G. *et al.* Brain structural trajectories over the adult lifespan. *Hum. Brain Mapp.* **33**, 2377–2389 (2012).
40. Kievit, R. A. *et al.* Developmental cognitive neuroscience using latent change score models: A tutorial and applications. *Dev. Cogn. Neurosci.* **33**, 99–117 (2018).
41. Matusik, J. G., Hollenbeck, J. R. & Mitchell, R. L. Latent Change Score Models for the Study of Development and Dynamics in Organizational Research. *Organ. Res. Methods* **24**, 772–801 (2021).
42. McArdle, J. J. Latent Variable Modeling of Differences and Changes with Longitudinal Data. *Annu. Rev. Psychol.* **60**, 577–605 (2009).
43. Li, T.-R. *et al.* Association of white matter hyperintensities with cognitive decline and neurodegeneration. *Front. Aging Neurosci.* **16**, 1–16 (2024).
44. Kim, S. J. *et al.* The Effects of Longitudinal White Matter Hyperintensity Change on Cognitive Decline and Cortical Thinning over Three Years. *J. Clin. Med.* **9**, 2663 (2020).

We thank the reviewers and the editor for their constructive feedback, which has collectively strengthened our manuscript. We sincerely appreciate the opportunity to be considered for publication in *Nature Communications.* Below, we provide point-by-point responses to the reviewers' comments. Additionally, we have revised the manuscript in accordance with the editorial requests, for which we provide the separate response document.

Reviewer #1

R1.0: The authors have responded thoroughly to my comments and have made substantial efforts to improve the manuscript. In particular, they conducted additional analyses and provided clearer explanations of their methodological choices. Regarding the concern about the PACC5 score and potential practice effects, the issue is not fully resolved, and the inherent limitations of the measure remain. However, the authors have acknowledged this limitation and provided a reasonable justification for retaining PACC5 as the cognitive outcome. They also implemented additional modelling strategies and expanded the discussion to address this concern more explicitly. I appreciate the authors' efforts to carefully consider the reviewer feedback and to strengthen the methodological transparency of the study. Importantly, the remaining limitations related to the PACC5 score do not appear to materially affect the main findings or the overall interpretation of the results. Overall, I believe the authors have responded constructively and the manuscript has been significantly improved as a result of the revision.

Response: We thank the reviewer for their thoughtful evaluation of our revisions. We particularly appreciate their recognition of our efforts to balance a transparent discussion of the study's limitations with a clear justification of our methodological choices. The reviewer's constructive feedback has been invaluable in strengthening both the rigor and the clarity of our manuscript.

Reviewer #2

R2.0: The authors have carefully addressed the concerns raised in the previous round of review. The inclusion of new findings on plasma pTau181 and NfL is interesting. The revisions have improved the robustness of the manuscript, and I have no further major comments.

Response: We thank the reviewer for their positive evaluation of our work. We are pleased that the inclusion of the additional biomarker analyses was found to be of interest and that our revisions enhanced the robustness of the study.

Reviewer #3

R3.0: The authors have conducted a great deal of supplementary analysis work and the revised and newly reported results have addressed my previous concerns. Good job. After reading the remarkably revised manuscript, a new idea comes to my mind that although the joint modelling of brain structure and cognition gave a principled operationalization of brain maintenance, it also posed a big obstacle for other researchers or clinics to use the newly defined brain maintenance to evaluate the status of brain health, due to the need for sufficient data

Response: We thank the reviewer for their encouraging feedback and for raising the question of practical applicability. We agree that the proposed operationalisation of brain maintenance, like the related cognitive-reserve construct, depends on multimodal longitudinal data and is therefore not immediately deployable in every research or clinical setting. This reflects a structural feature of estimation within the reserve and resilience framework, where identifiability and stability of latent change parameters scale with the number of indicators, time points, and sample size.

To partially mitigate this barrier, we now provide the analysis pipeline — trivariate latent growth curve model (lavaan syntax), regression-based factor-score extraction, and the robust regression yielding the brain-maintenance index — as an open GitHub repository, accompanied by a synthetic example dataset that reproduces the workflow end-to-end (see Code Availability). This allows other groups to apply, audit, or extend the framework on alternative cohorts without requiring access to the DELCODE raw data. Two further

points alleviate the data-demand concern at the conceptual level: (i) because the brain-maintenance index is a linear projection of the latent slope estimates, the regression coefficients are in principle transferable across cohorts — analogous to brain-age scoring schemes (e.g. Cole & Franke, 2017 https://doi.org/10.1016/j.tins.2017.10.001), in which weights estimated in a reference sample are applied to score independent samples; (ii) the multivariate LGCM is identifiable under standard assumptions with three indicators per domain and $T \geq 3$ waves for a linear slope ($T \geq 4$ if a quadratic slope is included, as for MTLV-ratio in our model), which is within reach of many existing longitudinal cohorts. We emphasise, however, that quantitative validation of such cross-cohort transfer is a separate empirical task that we recommend as a follow-up direction.

We have addressed this concern in the limitations section of our discussion by stating:
"*Importantly, the proposed operationalization of brain maintenance requires sufficiently rich multimodal longitudinal data, which may limit its immediate applicability in some research and clinical settings. Large-scale longitudinal studies are needed to operationalize the construct in the first place, and to enable its replication and further refinement. While incorporating additional dimensions may allow for more comprehensive modelling approaches, such efforts also increase data demands for achieving robust and generalizable estimates of neurocognitive health outcomes.*"
